# Novel coumarins active against *Trypanosoma cruzi* and toxicity assessment using the animal model *Caenorhabditis elegans*

**DOI:** 10.1186/s40360-019-0357-z

**Published:** 2019-12-19

**Authors:** Fabiana Gomes Nascimento Soares, Gabriela Göethel, Luciano Porto Kagami, Gustavo Machado das Neves, Elisa Sauer, Estefania Birriel, Javier Varela, Itamar Luís Gonçalves, Gilsane Von Poser, Mercedes González, Daniel Fábio Kawano, Fávero Reisdorfer Paula, Eduardo Borges de Melo, Solange Cristina Garcia, Hugo Cerecetto, Vera Lucia Eifler-Lima

**Affiliations:** 1 Laboratório de Síntese Orgânica Medicinal/LaSOM, Programa de Pós-Graduação em Ciências Farmacêuticas, Universidade Federal do Rio Grande do Sul, Porto Alegre, RS Brazil; 2 Laboratório Toxicologia/LATOX, Programa de Pós-Graduação em Ciências Farmacêuticas, Universidade Federal do Rio Grande do Sul, Porto Alegre, RS Brazil; 3 Facultad de Ciencias-Facultad de Química, Universidad de la República, Montevideo, Uruguay; 4 Faculdade de Ciências Farmacêuticas, Universidade Estadual de Campinas, Campinas, SP Brazil; 5 Departamento de Química Orgânica, Instituto de Química, Universidade Estadual de Campinas, Campinas, SP Brazil; 6 Universidade Estadual do Oeste do Paraná, Centro de Ciências Médicas e Farmacêuticas, Cascavel, PR Brazil; 7 Centro de Ciências Médicas e Farmacêuticas, Universidade Estadual do Oeste do Paraná, Cascavel, PR Brazil

**Keywords:** *Caenorhabditis elegans*, *Trypanosoma cruzi*, Coumarins, Chagas disease, Structure-activity relationship

## Abstract

**Background:**

Chagas disease (CD) is a tropical parasitic disease. Although the number of people infected is very high, the only drugs available to treat CD, nifurtimox (Nfx) and benznidazole, are highly toxic, particularly in the chronic stage of the disease. Coumarins are a large class of compounds that display a wide range of interesting biological properties, such as antiparasitic. Hence, the aim of this work is to find a good antitrypanosomal drug with less toxicity. The use of simple organism models has become increasingly attractive for planning and simplifying efficient drug discovery. Within these models, *Caenorhabditis elegans* has emerged as a convenient and versatile tool with significant advantages for the toxicological potential identification for new compounds.

**Methods:**

Trypanocidal activity:

Forty-two 4-methylamino-coumarins were assayed against the epimastigote form of *Trypanosoma cruzi* (Tulahuen 2 strain) by inhibitory concentration 50% (IC_50_).

Toxicity assays:

Lethal dose 50% (LD_50_) and Body Area were determined by *Caenorhabditis elegans* N2 strain (wild type) after acute exposure.

Structure-activity relationship:

A classificatory model was built using 3D descriptors.

**Results:**

Two of these coumarins demonstrated near equipotency to Nifurtimox (IC_50_ = 5.0 ± 1 μM), with values of: 11 h (LaSOM 266), (IC_50_ = 6.4 ± 1 μM) and 11 g (LaSOM 231), (IC_50_ = 8.2 ± 2.3 μM). In *C. elegans* it was possible to observe that Nfx showed greater toxicity in both the LD_50_ assay and the evaluation of the development of worms. It is possible to observe that the efficacy between Nfx and the synthesized compounds (11 h and 11 g) are similar. On the other hand, the toxicity of Nfx is approximately three times higher than that of the compounds. Results from the QSAR-3D study indicate that the volume and hydrophobicity of the substituents have a significant impact on the trypanocidal activities for derivatives that cause more than 50% of inhibition. These results show that the *C. elegans* model is efficient for screening potentially toxic compounds.

**Conclusion:**

Two coumarins (11 h and 11 g) showed activity against *T. cruzi* epimastigote similar to Nifurtimox, however with lower toxicity in both LD_50_ and development of *C. elegans* assays. These two compounds may be a feasible starting point for the development of new trypanocidal drugs.

## Background

Chagas disease (CD), or American trypanosomiasis, is a tropical parasitic disease that affects approximately 6-7 million people worldwide, predominantly in America [1]. CD is caused by the flagellated protozoan *Trypanosoma cruzi*, often transmitted to humans and to other mammals by blood-sucking insects, triatomines [2]. Although the number of people infected is very high and most cases occur in rural areas in Latin American countries [3], the only drugs available to treat CD are Nifurtimox (Nfx) and Benznidazole, which are highly toxic and often ineffective, particularly in the chronic stage of the disease [4]. At the same time, no drug has emerged as an effective candidate for clinical trials in the last 30 years [5]. Therefore, the identification of lead compounds that can be easily taken forward into lead optimization to yield drug candidates to be tested in human clinical trials is an essential move for the development of safer and more effective drugs for the treatment of CD.

Coumarins are a large class of compounds that display a wide range of interesting biological properties such as anticoagulant [6], antimicrobial activities [7], antioxidant [8], and anticancer [9]. They are considered good examples of “privileged structures”, usually rigid, polycyclic heteroatomic systems that are capable of binding to multiple pharmacological targets, thus providing a viable starting point in the search for new drugs [10, 11]. Considering the use of “privileged structures” as a feasible strategy to design new antitrypanosomal drug candidates with favourable pharmacokinetic/toxicity profiles [12], the 2H-chromen-2-one nucleus of coumarins was elected as the main structural feature for the development of new lead compounds with potential trypanocidal activity. This planar ring system is composed of one aromatic ring, capable of establishing hydrophobic, π-π, CH-π and cation-π interactions, and one lactone ring, which contains two oxygen atoms that may interact via hydrogen bonding with a series of amino acid residues, such as serine, threonine, cysteine, asparagine, glutamine and tyrosine. The C-C double bond in the 2-pyrone ring is also essential for conferring planarity to the 2H-chromen-2-one core, allowing for charge delocalization between the carbonyl group and the aromatic ring [13].

Although several studies report the therapeutic potential of coumarins in parasitic diseases such as leishmaniasis, malaria and amebiasis [14], there are few papers describing bioactive coumarins in *Trypanosoma cruzi* [15–19]. Chalepin, a coumarin isolated from *Ruta Angustifolia* L. Pers, was shown to be active against *T. cruzi* with an IC_50_ = 64 μM. The authors propose as an action mechanism, the glycolytic enzyme glyceraldehyde 3-phosphate dehydrogenase inhibition [15]. The synthetic coumarin described by Oliaro-Bosso *et al.*, proposes the oxidosqualene cyclases as the anti-trypanosome target, with an IC_50_ = 0.36 μM [19]. Another natural coumarin, Soulamarin, showed an IC_50_ = 210.1 μM. The authors proposed that the mitochondrial dysfunction and the modification of the plasma membrane permeability as the mechanisms of action [18].

**Fig. 1:**
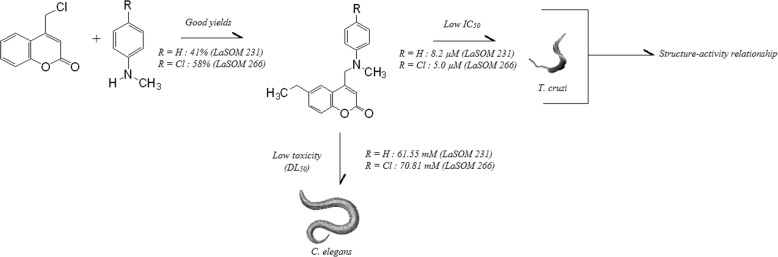
Workflow of new compounds development with anti-trypanosome activity

This article describes a complete workflow with new compound synthesis using an *in vitro* essay against *T. cruzi* and an *in vivo* toxicological test using the *C. elegans* model (Fig. 1). Highlight is made of the synthesis of a library of forty-two 4-methylamino-coumarins containing aromatic and aliphatic rings attached to the amino group, which had their trypanocidal activity assayed *in vitro* on *T. cruzi* epimastigotes. The *in vivo* toxicity profile was assessed for some of the most promising coumarins using the nematode *C. elegans* as a model for acute toxicity. Finally, a three-dimensional structure-activity relationship (SAR) study was carried out using Molecular Interaction Fields (MIF) by the GRid Independent Descriptors (GRIND) approach. GRIND program was used to determine groups that increase trypanocidal activity.

## Methods

### Chemistry

All chemicals were purchased as reagent grade and used without further purification. Solvents were distilled and/or dried in accordance with standard methods [20]. Column chromatography was performed on silica gel 60 (0.040-0.063 mm) using a hexane/ethyl acetate gradient. Melting points were determined on a Fisatom 431 apparatus and are uncorrected. Infrared spectra were recorded on Perkin Elmer model FT-Spectrum BRXII/Miracle ATR spectrometer. MS spectra were recorded on Q-TOF micro Waters high-resolution mass spectrometer, operating in electrospray ionization mode. Nuclear magnetic resonance spectra were recorded on Varian Inova (300 MHz for ^1^H nuclei), on Bruker Ascend (400 MHz for ^1^H nuclei) and on Anasazi (60 MHz for ^1^H nuclei) spectrometers. Chemical shifts (δ) are given in parts per million downfield from tetramethylsilane.

#### 6-substituted-4-chloromethylcoumarins (5, 6 and 7)

To a mixture of ethyl 4-chloroacetoacetate (1 mmol) and the corresponding phenol (1 mmol), H_2_SO_4_ (1 mL, 98%) was added dropwise. The reaction mixture was stirred for 24 hours at room temperature, and poured onto ice. The resulting precipitate was filtered off and recrystallized from ethanol-dioxane (9:1 v/v) [21].

#### General procedure for the preparation of compounds 9a-n, 10a-n and 11a-n

In a round bottom flask, a solution of 1 mmol of the 6-substituted-4-chloro methylcoumarin (5, 6 or 7) in THF (2 mL) was stirred at room temperature for 5 minutes for complete solubilization of the solute. Following this, 2 mmol of the corresponding amine (8a-n), 1.5 mmol of potassium iodide and a few droplets of water were added (the latter to achieve the complete solubilization of KI). The reaction mixture was heated to reflux for 1.5 hours and partitioned between a 10% NaOH solution and ethyl acetate (1:1 v/v). The organic phase was dried over MgSO4, filtered and concentrated, and the residue purified by column chromatography with a hexane/ethyl acetate gradient [22, 23].

### Anti-Trypanosoma cruzi activity

*Trypanosoma cruzi* epimastigotes (Tulahuen 2 strain) were grown at 28 °C in an axenic milieu (BHI-Tryptose) supplemented with 5% fetal bovine serum (FBS), as previously described [6, 24]. Cells from a 10-day-old culture (stationary phase) were inoculated into 50 mL of fresh culture milieu to give an initial concentration of 1 × 10^6^ cells per mL. Cell growth was followed by measuring the absorbance of the culture at 600 nm every day. Before inoculation, the milieu was supplemented with a given amount of the coumarin from a freshly prepared stock solution in DMSO (25 mM). The final concentration of DMSO in the culture medium never exceeded 0.4%, and the control was run in the presence of 0.4% DMSO and in the absence of drugs. No effect on epimastigote growth was observed due to the presence of up to 1% DMSO in the culture milieu. Nifurtimox (Nfx) was used as the reference trypanocidal drug. The percentage of growth inhibition (PGI) was calculated as follows: PGI (%) = {1 − [(Ap − A0p) / (Ac − A0c)]} × 100, where Ap = A600nm of the culture containing the studied compound at day 5; A0p = A600nm of the culture containing the studied compound just after addition of the inocula (day 0); Ac = A600nm of the culture in the absence of the studied compound (control) at day 5; A0c = A600nm in the absence of the studied compound at day 0. To determine the IC_50_ values (50% inhibitory concentrations), parasite growth was followed in the absence (control) and presence of increasing concentrations of the corresponding compound. At day 5, the absorbance of the culture was measured and related to the control. The IC_50_ value was taken as the concentration of the compound under study necessary to reduce the absorbance ratio to 50%.

### Toxicity assays on *Caenorhabditis elegans*

The nematode strain used was N2 (wild type), originally obtained from the *Caenorhabditis* Genetics Center (University of Minnesota, Twin Cities, MN, USA), which was maintained on nematode growth medium (NGM) plates seeded with *Escherichia coli* OP50 at 20°C. Synchronization of *C. elegans* cultures at the first larval stage (L1) was achieved by washing off the gravid nematodes from the plates into the centrifuge tubes, which were lysed with a bleaching mixture (1% NaOCl; 0.25 M NaOH), followed by flotation on a sucrose solution 30% (m/v) to separate the eggs from the dissolved worms and bacterial debris. The eggs were washed with M9 buffer (0.02 M KH_2_PO_4_, 0.04 M Na_2_HPO_4_, 0.08 M NaCl, and 0.001 M MgSO_4_) and allowed to hatch overnight (16 hours) in NGM agar plates without bacteria. After 16 hours it was obtained worms at L1 stage. Then, 1.500 L1 worms were exposed for 30 minutes at 20°C, by constant agitation in a rotator, at crescent doses ranging from 25 to 100 mM of coumarins in a 0.5% NaCl liquid media. Stock solutions of coumarins were made in DMSO, therefore worms treated with 0.5% NaCl and 0.5% DMSO were used as controls. After exposure, worms were washed 3 times with 0.5% NaCl to remove the treatments. Finally, worms were transferred to NGM recovery plates inoculated with *Escherichia coli* (OP50) for posterior assays.

#### Lethal dose 50% (LD_50_) evaluation

The LD_50_ of coumarins was determined in *C. elegans* after exposure. The worms were washed three times with NaCl buffer and placed on OP50-seeded NGM plates. The number of surviving worms on each plate was verified 24 hours after exposure. The lethality data was normalized with the controls and presented as a percentage. All the coumarins were tested in three independent experiments with each concentration tested in two replicates within each experiment.

#### Body area

For the evaluation of body area, 48 hours after exposure the adult worms (20 nematodes per treatment) were photographed and their body contour was measured. For this, the NGM plates were washed with distilled water and the resulting solution was transferred to a centrifuge tube, where the worm was allowed to settle, separating it from the bacteria in suspension. The process was repeated until the solution became clear. After this procedure, 15 μL of the solution with the worms was deposited on a blade covered by agarose and 15 μL of 2.25% levamisole was added. The pictures were acquired from 20 worms per treatment, which were manually contoured and measured with AxioVision software LE version 4.8.2.0 for Windows.

### Structure-activity relationship

Pentacle (Molecular Discovery Ltd) use the GRIND approach, which calculates 3D descriptors in an alignment-independent way [25]. Considering the results obtained in the evaluation of biological activity, it was decided to carry out a binary QSAR study based on the approach of [26] compounds with PGI less than 50%, which was set as inactive (1) and with PGI greater or equal to 50%, which was set as active (2). For each compound, 710 GRIND descriptors were used, based on combinations of the following molecular probes: DRY (hydrophobic interactions), O (hydrogen bond acceptor groups), N1 (hydrogen bond donor groups), and TIP (shape descriptor).

The initial set of MIFs was reduced to 60, using the variable selection method available in the Pentacle, Fractional Factorial Design (FDD). This process is performed by constructing models using Partial Least Squares (PLS), where a reduction of data occurs dimensionally. Most of the variations in the dataset are retained, and new variables (latent variables, LV), mutually orthogonal, are built [27]. In this step and in the subsequent ones, the descriptors had to be autoscaled, the basic process of data pre-processing used in QSAR studies [27]. In order to obtain models with simplified interpretation, the generated set went through a new stage of reduction of variables, where descriptors with absolute Pearson’s correlation coefficient (|r|) values with the binary activities greater than 0.2 were maintained, aiming to maintain only the MIF that presented the maximum amount of information relevant for the classification of the compounds. In the sequence, the Ordered Predictors Selection (OPS) variable selection method, available in the QSAR Modeling program, was used [27, 28]. After the variable selection, which also uses the PLS regression method, the model was constructed and refined using the PLS-DA [29, 30]. The aim of this last stage was to maximize the capacity of classification of the model. The necessary threshold adopted was 0.5, a value generally employed with PLS-DA, where compounds below this value were classified as active, and compounds above this were inactive. One of the advantages of using PLS-DA compared to other classification methods is that validation tools, such as calibration models, can be used. Thus, the quality of the data adjustment was assessed based on its coefficient of determination (R^2^) and the root mean square error of calibration (RMSEC). The results obtained were from cross-validation (using the values of Q2LOO and RMSECV), and by the visual inspection of the separation of the compounds in the two analyzed classes [31–33].

## Results

### Chemistry

**Fig. 2:**
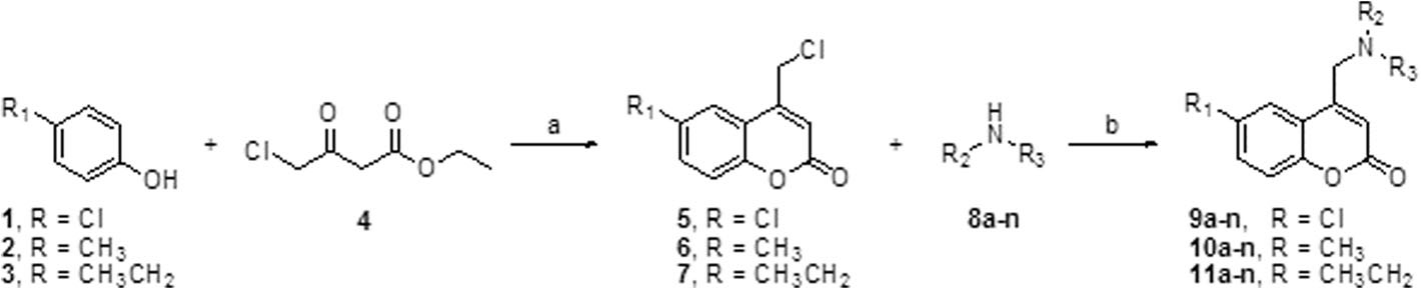
Reagents and conditions: (a) H2SO4, rt., overnight; (b) KI, THF-Water, 50 °C, 1.5 h

The original and green synthetic route for the 4-methylamino-coumarins is outlined in Fig. 2. Those of the 6-substituted-4-methylchlorochromen-2-ones (compounds 5, 6, and 7) were synthesized using the Pechmann reaction [21, 34], where the *p*-substituted phenols 1-3, undergo electrophilic aromatic substitution under acid catalysis with an ethyl 4-chloroacetoacetate 4, to give an intermediate that cyclizes by a transesterification reaction to produce the coumarin intermediates 5, 6 and 7 with good yields (71-89%). The N-alkylation reaction of primary and secondary amines (8a-n) was then achieved employing coumarins 5-7 and stoichiometric amounts of potassium iodide in THF/water [22, 23].

For the reactions, 8 aromatic amines with different substituents and 6 different cyclic aliphatic amines were used in order to structure-activity relationship studies. Additional file 1: Figures S1, S2 and S3, show the formed compounds and their yields.

### Biological studies

#### Anti-Trypanosoma cruzi activity

**Fig. 3:**
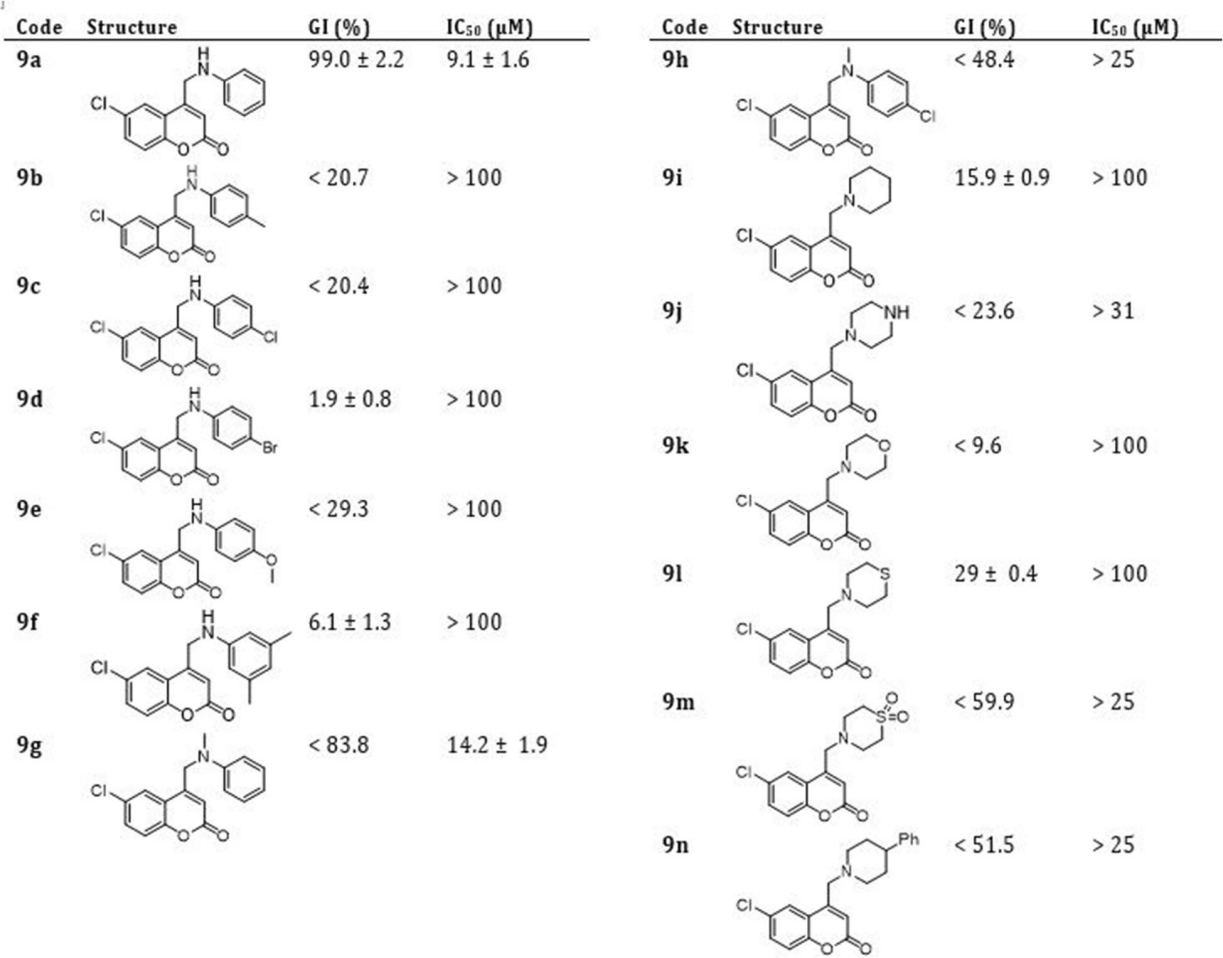
Effect of 4-methylamino-coumarins (9 h series) on the growth of *Tripanosoma cruzi* epimastigotes

**Fig. 4:**
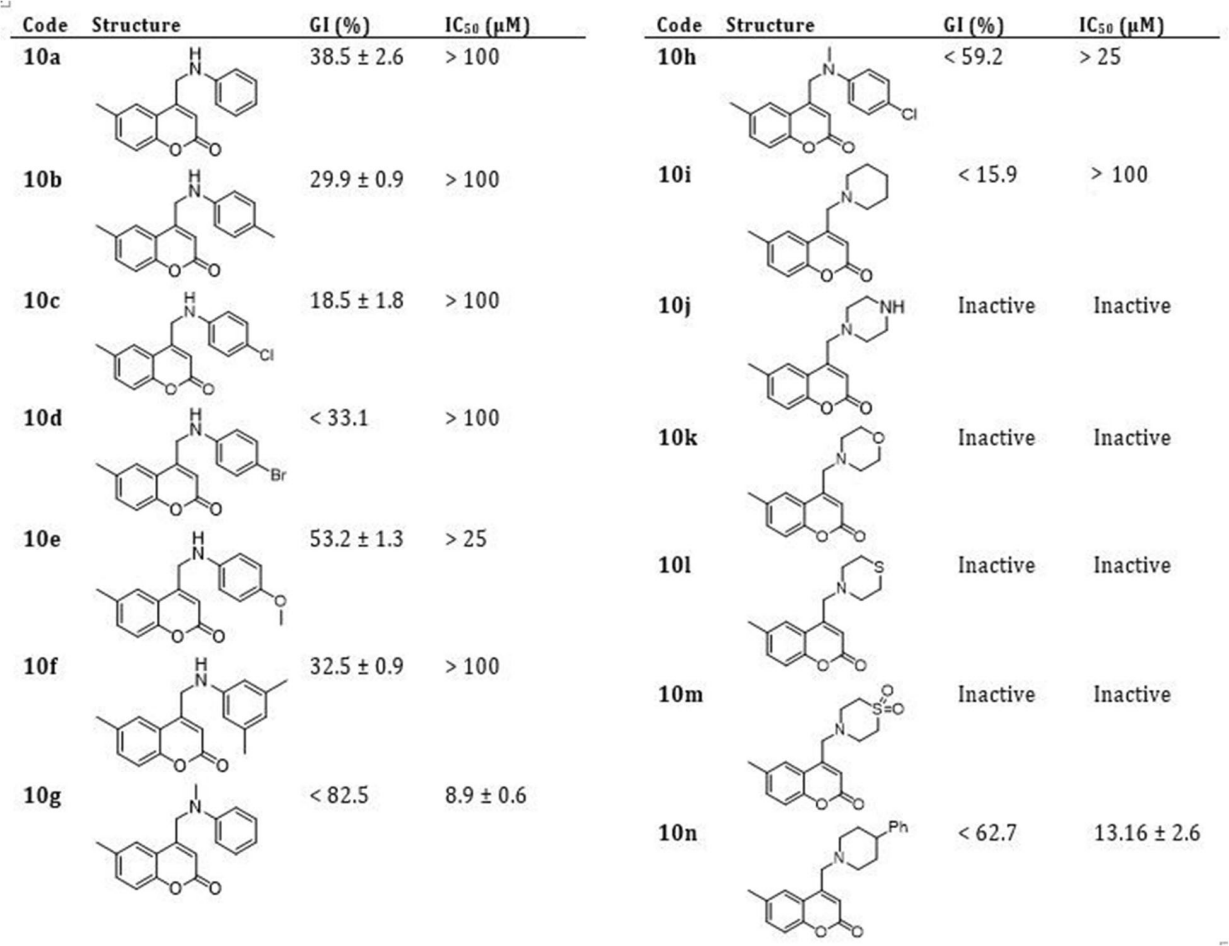
Effect of 4-methylamino-coumarins (10 h series) on the growth of *Tripanosoma cruzi* epimastigotes

**Fig. 5:**
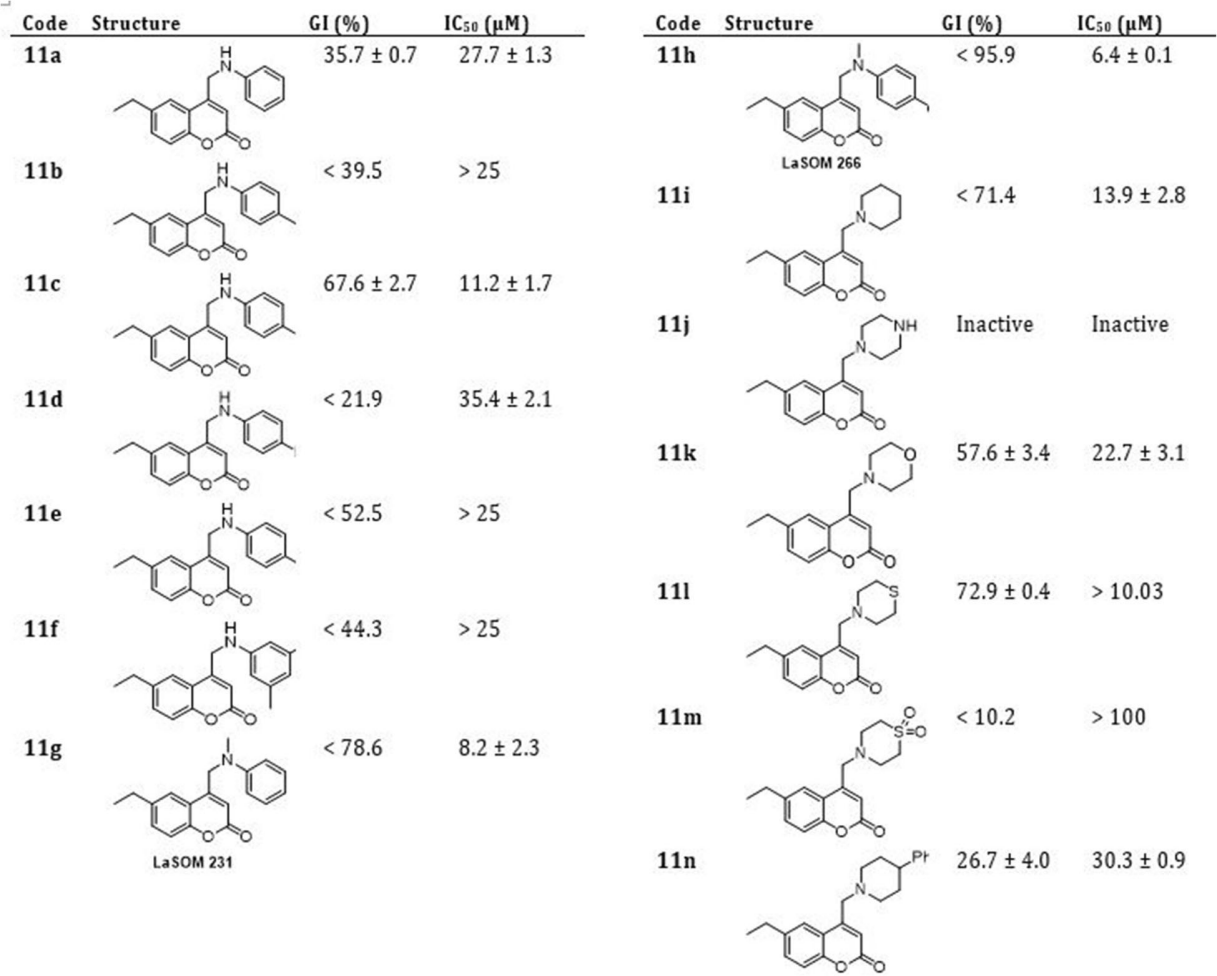
Effect of 4-methylamino-coumarins (11 h series) on the growth of *Tripanosoma cruzi* epimastigotes

Considering the potential existence of an intracellular epimastigote stage of *T. cruzi* as an intermediate between amastigote and trypomastigote forms [35, 36], as well as the readiness of experimentation, the forty-two 4-methylamino-coumarins were assayed against the epimastigote form of *T. cruzi*, Tulahuen 2 strain. In accordance with (Figs. 3, 4 and 5), in general, from the forty-two coumarins tested, fifteen showed a PGI higher than 50% against *T. cruzi* epimastigotes. The IC_50_ values of these 15 molecules ranged from 6.4 to > 25 μM, whereas Nifurtimox showed a IC_50_ equals to 5.0 ± 1.0 μM.

#### Toxicity assays on *Caenorhabditis elegans*

**Fig. 6:**
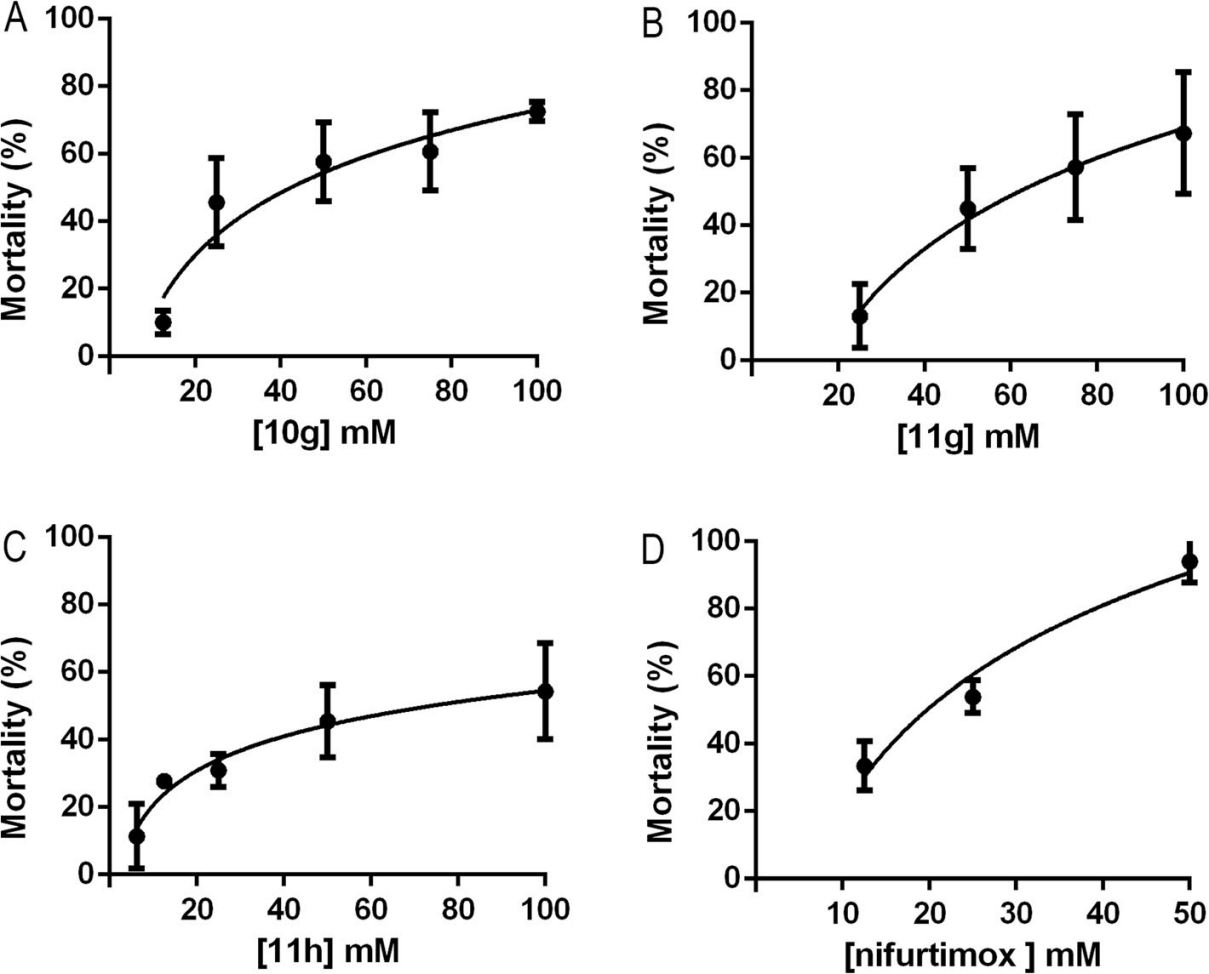
Lethality of 10 g (**a**), 11 g (**b**), 11 h (**c**) and Nifurtimox (**d**) after acute exposure of *C. elegans*. Data were obtained from at least 3 independent experiments performed in duplicate

The acute toxicity test was conducted using six different concentrations of coumarins and Nifurtimox. The results are represented by the concentration-response curves for each molecule, and are described in Fig. 6. These LD_50_ values show that the 4-methylcoumarins 11 h (LD_50_ = 73.4 mM), 11 g (LD_50_ = 61.7 mM) and 10 g (LD_50_ = 42.3 mM) present less toxicity that the Nifurtimox (LD_50_ = 19.50 mM).

**Fig. 7:**
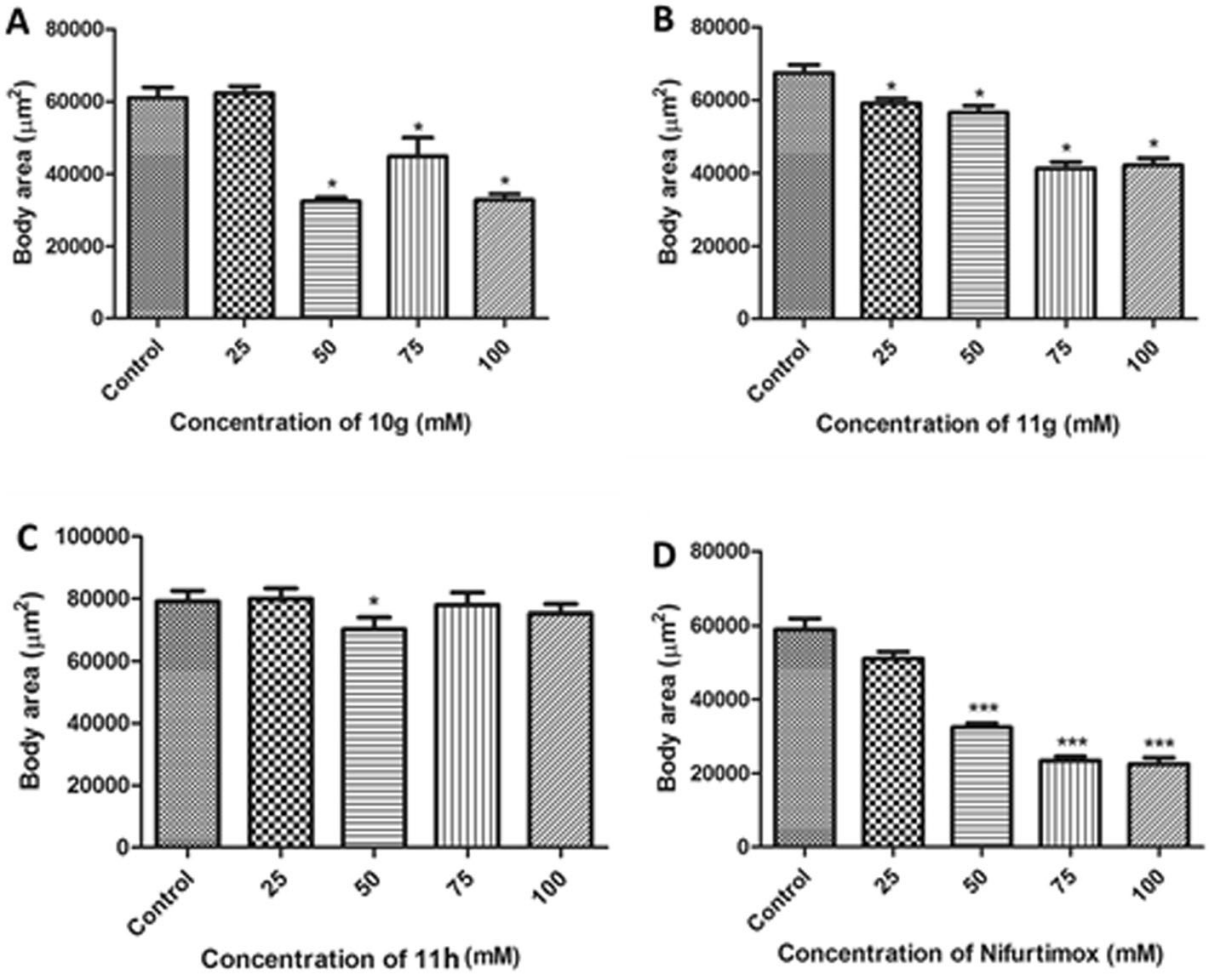
Body areas of *C. elegans* after acute treatment (30 min) with 10 g, (**a**) 11 g (**b**), 11 h (**c**) and Nifurtimox (**d**). Results are expressed as mean ± SEM (*n* = 3 independent experiments performed in duplicate). *p < 0.05, **p < 0.01, ***p < 0.001 compared to the control group. Statistical comparisons were made using one way ANOVA/Bonferroni post-hoc test

Another toxicity endpoint used to verify potential toxic effects of the new coumarins was the evaluation of the worm development, which was monitored by measuring body area. In this study, the normal development of *C. elegans* was affected by acute exposure to Nifurtimox, 11 h, 11 g, and 10 g (Fig. 7). Compared with the control, Nifurtimox (Fig. 7d), a significant reduction of body area was observed at 50, 75 and 100 mM concentrations *(p* < 0.01, ANOVA/Bonferroni). 11 h (Fig. 7c) presented a significant reduction in the body area at 50 mM concentrations (*p* < 0.05, ANOVA/Bonferroni). The worms treated with 11 g (Fig. 7b) showed a significant *(p* < 0.05, ANOVA/Bonferroni) reduction in body area in the concentrations of 25, 50, 75 and 100 mM. For 10 g (Fig. 7a), a significant (*p* < 0.05, ANOVA/Bonferroni) reduction of body area was observed for the concentrations of 50, 75 and 100 mM.

#### Structure-activity relationship

Using the FDD, a 3D model with 60 MIF descriptors was obtained. Following this and after the exclusion of the descriptors with |r| < 0.2 with the binary vector y, the OPS variable selection method was used, generating a PLS model formed by only 14 descriptors. In the last step, the model was refined in the Pirouette program from the analysis of the regression vector, correlogram and loadings plot obtained using the PLS-DA method. Finally, a model was formed consisting of only 11 MIF descriptors (equation) and four latent variables that encode 60.522% of information (LV1: 16.548%; LV2: 15.633%; LV3: 8.868%; LV4: 19.473%).

$$ \mathbf{Class}=0.405+1.182\ast \left(8\_\mathrm{DRY}-\mathrm{DRY}\right)+1.691\ast \left(20\_\mathrm{DRY}-\mathrm{DRY}\right)+2.574\ast \left(151\_\mathrm{TIP}-\mathrm{TIP}\right)+0.966\ast \left(163\_\mathrm{TIP}-\mathrm{TIP}\right)-0.638\ast \left(169\_\mathrm{TIP}-\mathrm{TIP}\right)-1.098\ast \left(170\_\mathrm{TIP}-\mathrm{TIP}\right)-0.756\ast \left(193\_\mathrm{DRY}-\mathrm{O}\right)-1.086\ast \left(261\_\mathrm{DRY}-\mathrm{N}1\right)+1.047\ast \left(285\_\mathrm{DRY}-\mathrm{TIP}\right)+0.716\ast \left(455\_\mathrm{N}1-\mathrm{TIP}\right)+0.640\ast \left(456\_\mathrm{N}1-\mathrm{TIP}\right)\ {\mathbf{R}}^{\mathbf{2}}=0.737;\mathbf{RMSEC}=0.242;\mathbf{Q}\mathbf{2}\mathbf{LOO}=0.613;\mathbf{RMSEC}\mathbf{V}=0.293. $$

Table 1 shows the degrees of importance of each descriptor. It is possible to note that four descriptors are related to probe TIP-TIP, two to N1-TIP, one to DRY-TIP, two to DRY-DRY, one to DRY-O, and one to DRY-N1.

**Table 1:** Importance of each GRIND descriptor of the Model

Autoscaled coefficient	Descriptor	Field	Distance range
−0.427	193	DRY-O	3.6–4.0
−0.363	261	DRY-N1	12.4–12.8
0.325	151	TIP-TIP	5.2–5.6
−0.319	170	TIP-TIP	12.8–13.2
0.313	20	DRY-DRY	8.0–8.4
0.307	163	TIP-TIP	10.0–10.4
0.305	455	N1-TIP	16.4–16.8
0.271	456	N1-TIP	16.8–17.2
0.224	8	DRY-DRY	3.2–3.6
−0.195	169	TIP-TIP	12.4–12.8
0.148	285	DRY-TIP	3.6–4.0

## Discussion

The experimental design was performed in order to demonstrate the in vivo alternative model *C. elegans* applied to a drug discovery context. In this paper, it was not only possible to conceive the *C. elegans* model, but also it was possible to highlight new compounds with anti-*Trypanosoma cruzi* activity.

Our workflow started with the synthesis of a focused library from the reaction of 3 coumarins × 14 amines resulting in 42 compounds (9a-9n; 10a-10n; 11a-11n), with good molecular diversity. The choices were: 1. To link non-aromatic (primary and secondary amines) and aromatic (several patterns of substitution) amines to 4-methyl position; 2. To check the importance or not of a tertiary amine in this position; for this purpose, we have compounds with an additional methyl group attached to nitrogen; and 3. Substitution at the C-6 position of coumarin nucleus with groups with different electronegativity or volume (ethyl, methyl or Cl).

In the next step, we subjected the compounds to *in vitro* inhibition assays using the epimastigote form. The compounds with the highest inhibition percentage (>82%) have aromatic amines bearing the coumarin core and, among them, three compounds with no substitution at the benzene ring (9a, 9g, and 10g) and one with 4-Cl substitution (11h). On the other hand, four coumarins presented IC_50_ < 10 μM (9a, 10g, 11g (LaSOM 231) and 11h (LaSOM 266). One of these compounds, coumarin 11h, presented an IC_50_ value (6.4 ± 1 μM) very close to Nifurtimox. Although only Nifurtimox was used as the positive control in this study, it was previously determined that Nifurtimox and Benznidazole have very similar IC_50_ values when tested *in vitro* against the epimastigote form of *T. cruzi*, Tulahuen 2 strain [37]. Indeed, it can be observed that the three most active 11h (IC_50_ = 6.4 ± 1 μM), 11g (IC_50_ = 8.2 ± 2.3 μM) and 10g (IC_50_ = 8.9 μM) have the methyl group attached to nitrogen. With regard to the modifications at position 6 of the coumarin core, compounds with the ethyl group were among the most active, while Cl at this position furnished the less active compounds.

A recent study evaluated 19 tricyclic coumarins through a modeling study and *in vitro* study similar to our work. The study demonstrated for most coumarins evaluated promising activity against the intracellular forms of *T. cruzi*, with ten compounds showing IC_50_ values lower than Benznidazole [38].

Following the current trends in medicinal chemistry for using the alternative *C. elegans* model in the early stages of the discovery of new drugs [39, 40], our research group recently published the use of this method to evaluate the safety of new potentially antitumor compounds [41].

In this study, the evaluation was made of the toxicity of new compounds with potential activity against Chagas disease, using this alternative method. In order to then check the *in vivo* toxicity of the three hits 11h, 11g and 10g, selected above, the LD_50_ investigation of these compounds was performed as studies have shown that *C. elegans* have a good correlation with rodent oral LD_50_ ranking. To the best of our knowledge this is the first report of the LD_50_ of Nifurtimox (Nfx) performed in *C. elegans*. With this propose in mind, the three new coumarins 11h, 11g, 10g and the standard drug Nifurtimox, were evaluated in *C. elegans*. According to the literature, Nifurtimox has presented several toxic effects, such as: neurotoxicity, testicular damage, ovarian toxicity and deleterious effects in adrenal, colon, oesophageal and mammary tissue [42]. It appeared to be important to know if the two more active coumarins had the same toxicity of Nifurtimox; therefore, the coumarins and the standard drug were evaluated in order to compare the toxicities among them. Of likely importance for the future is the use of *C. elegans* as a model system for pre-screening drug discovery. It is to be hoped that this will provide new anthelmintics that less toxic [43, 44]. *C. elegans*, which is about 1 mm in length as an adult, can be cultured in high-throughput format for multiple generations, allowing the identification of molecules that perturb the worm at any point during its life cycle [45, 46].

A study published by a group that used *C. elegans* as a model for the discovery of new anthelmintic revealed that by first screening in *C. elegans*, they may have lost molecules that would be effective in killing parasites but are ineffective in model *C. elegans*. However, they concluded that the speed and ease with which molecules can be traced using *C. elegans* can overcome the disadvantages it carries as a primary screening system and that it can be used to evaluate hundreds of thousands of molecules at multiple concentrations at a fraction of the cost and time reduced [47].

A study using albendazole in *C. elegans* demonstrated the EC_50_ of 18.43 μM for the worm. This concentration showed damage to the body wall of adults and larvae. It was observed intense desquamation of the cuticle of the larvae and the surface of the eggs, preventing their hatching and development. This study reaffirmed the use of *C. elegans* as a screening system for compounds with potential anthelmintic activity and showed the effects of albendazole on the different life stages of these worms [48].Considering that the acute toxicity tests were performed with a concentration about 1000 times above those used in the efficacy trials (IC_50_), these three new coumarins 11 g, 11 h and 10 g can be considered safe in comparison to Nifurtimox. Furthermore, the evaluation of the worm development, expressed by the body area measurement, is a good parameter to evaluate toxic effects in *C. elegans*, considering that the growth of *C. elegans* is determined by a conservative genetic regulatory pathway [49].

In order to complement the workflow, we added a QSAR study that, through a mathematical model, emphasizes the importance of certain chemical groups substitutions. The results obtained for the fit (R2) and internal prediction ability (Q2LOO) indicate that the model explains and predicts information at the levels recommended by the literature [31–33].

The descriptor analysis emphasizes that the importance of steric characteristics related to TIP descriptors predominates in the model. Despite this, the two most important descriptors (193_DRY-O e 261_DRY-N1) indicate that the presence of hydrophobic groups and groups capable of forming hydrogen bonds at the indicated distances tend to lead to molecules with a degree of inhibition below 50%.

**Fig. 8:**
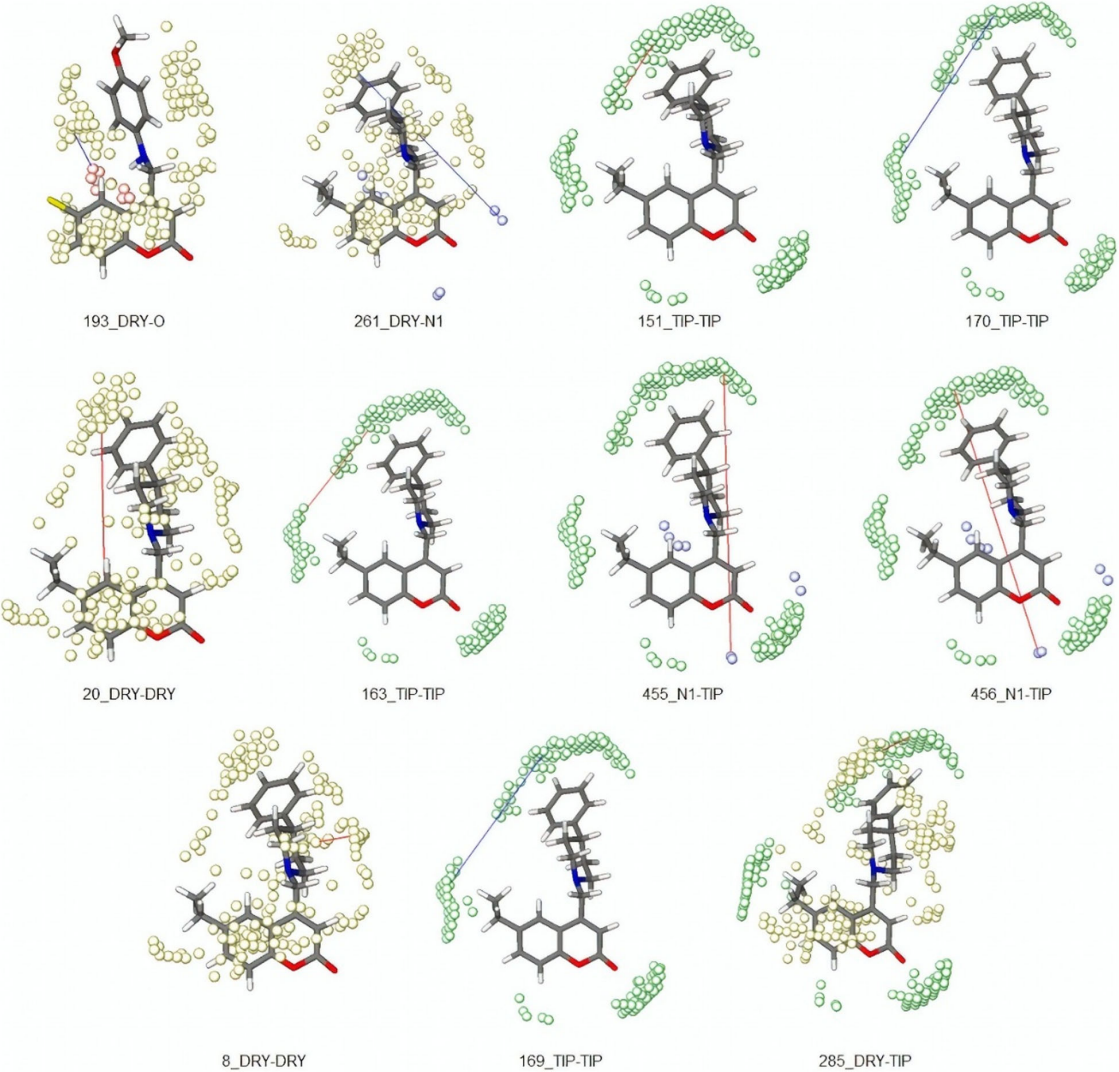
GRIND selected descriptors of Model 1 associated with active compounds

**Fig. 9:**
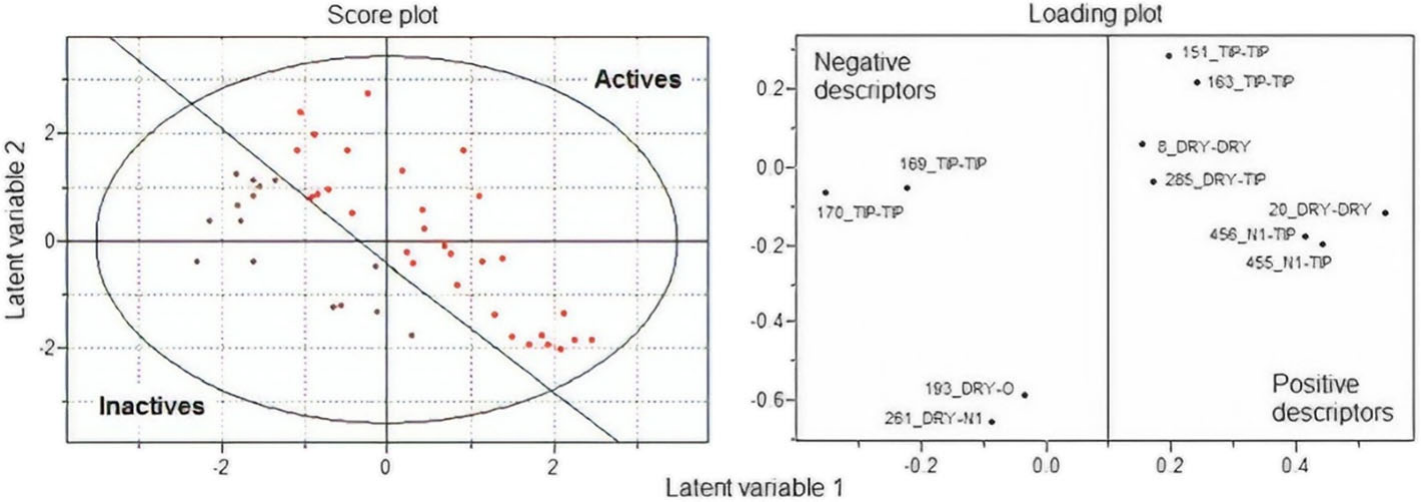
Plot of the loading and score vectors

It can be seen that for both descriptors this may mean that the pharmacophoric point formed by hydrophobic groups (R1 position) and electronegative atoms (R2 position) is detrimental to activity as observed in compounds **9c**, **9d**, **10c**, **10d** and **11d**. On the other hand, volume at the R2 position leads to the most important steric descriptor of the model (151_TIP-TIP), which has a positive influence on the activity, indicating that the substituents present in this position may be filling a bulky cavity at the binding site as can be noticed in compounds 11 g and 11 h. The descriptors 169_TIP-TIP and 170_TIP-TIP show that the positioning of bulky groups at a distance of 12.4 to 13.2 (distances formed by the distances between groups R1 and R2) are unfavourable to the activity. The ideal distance between these two groups is given by the descriptor 163_TIP-TIP (10.0–10.4 Å). The descriptors 455_N1-TIP and 456_N1-TIP show that a relation between the bulk group used in position R2 and the lactonic oxygen in the distance of 16.4 to 17.2 Å is important for the activity. In addition, it is possible to observe in the selected inactive molecule that this pharmacophoric characteristic is absent as it can be seen in compounds 9e, 9i, 9j, 10i and 10j. The selected DRY-DRY descriptors, although encoding different pharmacophoric points, are both related to the hydrophobicity of the groups used in the R2 position. Finally, similar interpretation can be made for the 285_DRY-TIP descriptor, the less important of the models. This shows that the hydrophobicity and volume used in the R1 and R2 position should also be favoured, and for both they seem to indicate that lower volume hydrophobic substituents can be detrimental to the activity (Fig. 8). Each one of the descriptors of model 1 is presented in Fig. 9. In Fig. 9, the graphs of weights and scores obtained by PLS-DA are presented, showing the classification of the data set obtained through latent variables 1 and 2, and the distribution of the descriptors in relation to each of these variables. This result indicates that the obtained model is capable of discriminating with good reliability those compounds having a degree of inhibition below or above 50%, and thus the results obtained herein may be useful to direct the synthesis of new derivatives with an acceptable degree of inhibition.

*C. elegans* has proved to be an extremely useful model organism for toxicity studies for pre-screening new drugs. We can observe as limitations of the model used in our study: *C. elegans* do not possess some mammalian organs, such as lungs, heart, kidneys and liver. In addition, changes in temperature and humidity may alter test results. Another limitation of the study was to respect the solubility limit of the molecules we evaluated, since if we increase the concentrations we could have problems of solubility. Therefore, higher concentrations than those described in the study were not evaluated.

Although it was constructed from coumarin-like structures, the QSAR model was based on molecular interaction fields, which have established the best distances and interaction groups related to the activity. These observations may guide the synthesis of new compounds with better profile against *T. cruzi*. Nevertheless, the constructed model is limited by the PGI values and by the cutoff point settled to obtain the classificatory model, which is helpful to select features correlated to the activity. However, it lacks information regarding quantitative values, thus turning the data extrapolation difficult to be predicted.

## Conclusions

The synthesis of a library of forty two 4-methylamino-coumarins with aromatic and aliphatic rings attached to the amino groups was accomplished using green chemistry conditions. The library activity was assayed in vitro against *T. cruzi* epimastigotes and two of these coumarins demonstrated to be nearly equipotent to Nifurtimox (IC_50_ = 5.0 ± 1 μM), 11 h (IC_50_ = 6.4 μM) and 11 g (IC_50_ = 8.2 μM). Also, toxicity assay performed on *C. elegans* showed that these two compounds 11 h (DL_50_ = 70.81 mM) and 11 g (DL_50_ = 61.50 mM) were clearly less toxic than Nifurtimox (DL_50_ = 19.50 mM). In addition, the structure-activity relationship study showed that hydrophobic groups in R1 position and electronegative atoms in R2 position are detrimental to activity. In conclusion, 11 h and 11 g may be a feasible starting point for the development of new trypanocidal compounds. Further studies will be made in order to determine experimentally the mechanism of action of the coumarins using labeled strains of *C. elegans.*

## Supplementary information

**Additional file 1: **Novel coumarins active against Trypanosoma cruzi and toxicity assessment using the animal model *Caenorhabditis elegans.*

## Data Availability

Not applicable.
