# Supplementary material for: Novel coumarins active against Trypanosoma cruzi and toxicity assessment using the animal model Caenorhabditis elegans
Source: BMC Pharmacol Toxicol. 2019 Dec 19;20(Suppl 1):76. doi: 10.1186/s40360-019-0357-z (PMC6921407; doi:10.1186/s40360-019-0357-z)
Supplement: Supplementary file 1 — Additional file 1: Novel coumarins active against Trypanosoma cruzi and toxicity assessment using the animal model Caenorhabditis elegans. [file 40360_2019_357_MOESM1_ESM.docx]

**ADDITIONAL FILE 1**

**Novel coumarins active against Trypanosoma cruzi and toxicity assessment using the animal model Caenorhabditis elegans.**

Fabiana Gomes Nascimento Soares^1†^, Gabriela Göethel^2†^, Luciano Porto Kagami^1^, Gustavo Machado das Neves^1^, Elisa Sauer^1^, Estefania Birriel^3^, Javier Varela^3^, Itamar Luís Gonçalves^1^, Gilsane Von Poser^1^, Mercedes González^3^, Daniel Fábio Kawano^4,5^, Fávero Reisdorfer Paula^6^, Eduardo Borges de Melo^7^, Solange Cristina Garcia^2^, Hugo Cerecetto^3^, Vera Lucia Eifler-Lima^1*^

**

**

**Figure S1.** Synthesis of compounds 9a-n.

**

**

**Figure S2**. Synthesis of compounds 10a-n.

**

**

**Figure S3.** Synthesis of compounds 11a-n**.**

**NMR Chemical Shifts of Compounds (5-7), (9a-n), (10a-n)**

6-chloro-4-(chloromethyl)-2H-chromen-2-one (5). Yield 71%; mp 130-133 °C; HRMS 30eV (Q-TOF) (m/z): calcd. for C_10_H_6_Cl_2_O_2_ [M+H]+: 228.9778, found 228.9825. ^1^H NMR (300 MHz, CDCl_3_) δ (ppm): 4.63 (d, J = 0.8 Hz, 2H), 6.60 (s, 1H), 7.31 (d, J = 8.8 Hz, 1H), 7.51 (dd, J = 8.8, 2.4 Hz, 1H), 7.63 (d, J = 2,4 Hz, 1H). ^13^C NMR (75 MHz, CDCl_3_) δ (ppm): 40.94, 116.92, 118.38, 118.85, 123.82, 130.01, 132.24, 148.47, 152.23, 159.49.

4-(chloromethyl)-6-methyl-2H-chromen-2-one (6). Yield 89%; mp 150-152 °C; HRMS 30eV (Q-TOF) (m/z): calcd. for C_11_H_9_ClO_2_ [M+H]+: 209.0325, found 209.0368. ^1^H NMR (300 MHz, CDCl_3_) δ (ppm): 0.93 (s, 3H), 4.34 (d, J = 0.6 Hz, 2H), 6.20 (s, 1H), 6.92 (d, J = 8.5 Hz, 1H), 7.05 (dd, J = 8.5, 1.7 Hz, 1H), 7.09 (d, J = 1.5 Hz, 1H). ^13^C NMR (75 MHz, CDCl_3_) δ (ppm): 22.35, 35.29, 109.58, 110.96, 111.18, 116.76, 126.14, 134.64, 143.53, 146.00, 154.49.

4-(chloromethyl)-6-ethyl-2H-chromen-2-one (7). Yield 82%; mp 62-64 °C; HRMS 30eV (Q-TOF) (m/z): calcd. for C_12_H_11_ClO_2_ [M+H]+: 223.0481, found 223.0526. ^1^H NMR (300 MHz, CDCl_3_) δ (ppm): 1.28 (t, J = 7.6 Hz, 3H), 2.73 (q, J = 7.6 Hz, 2H), 4.70 (s, 2H), 6.55 (s, 1H), 7.27 (d, J = 8.3 Hz, 1H), 7.40 (dd, J = 8.5, 1.7Hz, 1H), 7.44 (d, J = 1.4 Hz, 1H). ^13^C NMR (75 MHz, CDCl_3_) δ (ppm): 15.70, 28.40, 41.34, 115.63, 117.01, 117.23, 122.80, 132.19, 140.69, 149.58, 152.02, 160.54.

6-chloro-4-[(phenylamino)methyl]- 2H-chromen-2-one (9a). Yield 51%; mp 187-188 °C. ^1^H NMR (300 MHz, CDCl_3_) δ (ppm): 4.42 (s, 2H), 6.59 (s, 1H), 6.62 (d, J = 7.6 Hz, 1H), 7.22 (t, J = 7.9 Hz, 1H), 7.28 (s, 1H), 7.35 (dd, J = 11.7, 6.0 Hz, 2H), 7.54 (dd, J = 8.8, 2.3 Hz, 1H), 7.68 (s, 1H).

6-chloro-4-{[(4-methylphenyl)amino]methyl}-2H-chromen-2-one (9b). Yield 47%; mp 201 °C. ^1^H NMR (400 MHz, CDCl_3_) δ (ppm): 2.24 (s, 3H), 4.46 (s, 2H), 6.51 (d, J = 8.3 Hz, 2H), 6.58 (s, 1H), 7.00 (d, J = 8.2 Hz, 2H), 7.31 (d, J = 8.8 Hz, 1H), 7.50 (dd, J = 8.8, J = 2.1 Hz, 1H), 7.62 (d, J = 2.2 Hz, 1H). ^13^C NMR (100 MHz, CDCl_3_) δ (ppm): 20.38, 44.65, 112.99, 114.33, 118.74, 119.34, 123.17, 128.07, 129.78, 129.99, 131.76, 144.27, 151.58, 152.18, 160.19.

6-chloro-4-{[(4-chlorophenyl)amino]methyl}-2H-chromen-2-one (9c). Yield 52%; mp 205-207 °C. ^1^H NMR (400 MHz, DMSO-d_6_) δ (ppm): 4.55 (d, J = 4.5 Hz, 2H), 6.32 (s, 1H); 6.60 (d, J = 8.8 Hz, 1H); 7.21 (d, J = 8.8 Hz, 1H); 7.45 (d, J = 8.9 Hz, 1H); 7.67 (dd, J = 8.9, 2.4 Hz, 1H); 7.96 (d, J = 2.4 Hz, 1H). ^13^C NMR (100 MHz, DMSO-d_6_) δ (ppm): 43.01; 107.43; 112.85; 114.47; 115.97; 118.63, 119.47; 124.46, 128.80, 131.42, 131.66, 131.85, 147.20, 151.87, 152.98, 159.62.

4-{[(4-bromophenyl)amino]methyl}-6-chloro-2H-chromen-2-one (9d). Yield 31%; mp 231-232 °C. ^1^H NMR (60 MHz, DMSO-d_6_) δ (ppm): 4.28 (d, J = 6.0 Hz, 2H), 6.05 (s, 1H), 6.18-6.49 (m, 3H), 6.71-7.09 (m, 2H), 7.16-7.41 (m, 1H), 7.70 (d, J = 2.2 Hz, 1H).

6-chloro-4-{[(4-methoxyphenyl)amino]methyl}-2H-chromen-2-one (9e). Yield 69%; mp 192-194 °C. ^1^H NMR (400 MHz, CDCl_3_) δ (ppm): 3,74 (s, 3H), 4.44 (d, J = 1.5 Hz, 2H), 6.52-6.60 (m, 3H), 6.75-6.81 (m, 2H), 7.31 (d, J = 8.8, 1H), 7.50 (dd, J = 8.8, J = 2.4 Hz, 1H), 7.63 (d, J = 2.4 Hz, 1H). ^13^C NMR (100 MHz, CDCl_3_) δ (ppm): 45.18, 55.78, 114.13, 114.31, 115.08, 118.02, 119.34, 123.20, 129.77, 131.76, 140.68, 151.73, 152.14, 152.88, 160.24.

6-chloro-4-{[(3,5-dimethylphenyl)amino]methyl}-2H-chromen-2-one (9f). Yield 56%; mp 192-194 °C. ^1^H NMR (400 MHz, CDCl_3_) δ (ppm): 2.22 (s, 6H), 4.47 (s, 2H), 6.22 (s, 2H), 6.44 (s, 1H), 6.57 (s, 1H), 7.32 (d, J =8,8 Hz, 1H), 7.51 (dd, J = 8.8, 2.3 Hz, 1H), 7.63 (d, J = 2.3 Hz, 1H). ^13^C NMR (100 MHz, CDCl_3_) δ (ppm): 21.47, 44.35, 110.74, 114.19, 118.70, 119.37, 120.66, 123.21, 129.78, 131.74, 139.21, 146.82, 151.82, 152.14, 160.27.

6-chloro-4-{[methyl(phenyl)amino]methyl}-2H-chromen-2-one (9g). Yield 38%; mp 200-201 °C. ^1^H NMR (300 MHz, CDCl_3_) δ (ppm): 3.13 (s, 3H), 4.64 (d, J = 1.4 Hz, 2H), 6.39 (s, 1H), 6.69 (d, J = 8.1 Hz, 2H), 6.82 (t, J = 7.3 Hz, 1H), 7.23-7.30 (m, 2H), 7.36 (d, J = 8.8 Hz, 1H), 7.54 (dd, J = 8.8, 2.4 Hz, 1H), 7.60 (d, J = 2.3 Hz, 1H). ^13^C NMR (75 MHz, CDCl_3_) δ (ppm): 39.05, 53.66, 112.26, 113.82, 117.88, 118.79, 119.27, 123.00, 129.46, 129.78, 131.82, 148.46, 150.47, 152.26, 160.13.

6-chloro-4-{[(4-chlorophenyl)(methyl)amino]methyl}-2H-chromen-2-one (9h). Yield 61%; mp 185-186 °C. ^1^H NMR (300 MHz, CDCl_3_) δ (ppm): 3.12 (s, 3H), 4.62 (d, J = 1.3 Hz, 2H), 6.33 (s, 1H), 6.59 (d, J = 9.1 Hz, 2H), 7.19 (d, J = 9.1 Hz, 2H), 7.36 (d, J = 8.7 Hz, 1H), 7.51-7.60 (m, 2H). ^13^C NMR (75 MHz, CDCl_3_) δ (ppm): 39.43, 53.84, 113.55, 113.94, 119.03, 119.28, 123.00, 123.11, 129.45, 130.04, 132.13, 147.20, 150.12, 152.44, 160.17.

6-chloro-4-(piperidin-1-ylmethyl)-2H-chromen-2-one (9i). Yield 44%; mp 181-182 °C. ^1^H NMR (300 MHz, CDCl_3_) δ (ppm): 1.50-1,53 (m, 6H), 2.45-2,47 (m, 4H), 4.64 (d, J = 1.4 Hz, 2H), 6.39 (s, 1H), 6.69 (d, J = 8.1 Hz, 2H), 6.82 (t, J = 7.3 Hz, 1H), 7.23-7.30 (m, 2H), 7.36 (d, J = 8.8 Hz, 1H), 7.54 (dd, J = 8.8, 2.4 Hz, 1H), 7.60 (d, J = 2.3 Hz, 1H).

6-chloro-4-(piperazin-1-ylmethyl)-2H-chromen-2-one (9j). Yield 26%; mp > 300 °C; HRMS 30eV (Q-TOF) (m/z): calcd. for C_14_H_15_ClN_2_O_2_ [M+H]^+^: 279.0856, found 279.0828.

6-chloro-4-(morpholin-4-ylmethyl)-2H-chromen-2-one (9k). Yield 48%; mp 177-178 °C. ^1^H NMR (300 MHz, CDCl_3_) δ (ppm): 2.53-2.62 (m, 4H), 3.62 (m, 2H), 3.73-3.80 (m, 4H), 6.59 (s, 1H), 7.29 (d, J = 8.1 Hz, 1H), 7.48 (dd, J = 8.8, 2.4 Hz, 1H), 7.83 (d, J = 2.4 Hz, 1H). ^13^C NMR (75 MHz, CDCl_3_) δ (ppm): 53.98, 59.43, 67.06, 116.25, 118.67, 120.08, 124.64, 129.57, 131.90, 150.66, 152.42, 160.38.

6-chloro-4-(thiomorpholin-4-ylmethyl)-2H-chromen-2-one (9l). Yield 46%; mp 152-153 °C. ^1^H NMR (400 MHz, CDCl_3_) δ (ppm): 2.68 – 2.72 (m, 4H), 2.79-2.86 (m, 4H), 3.62 (d, J = 1.2 Hz, 2H), 6.56 (s, 1H), 7.29 (d, J = 8.9 Hz, 1H), 7.48 (dd, J = 8.8, J = 2.4 Hz, 1H), 7.79 (d, J = 2.4 Hz, 1H). ^13^C NMR (100 MHz, CDCl_3_) δ (ppm): 28.19, 55.49, 59.66, 116.09, 118.66, 120.04, 124.53, 129.71, 131.86, 150.93, 152.38, 160.37.

6-chloro-4-[(1,1-dioxidothiomorpholin-4-yl)methyl]-2H-chromen-2-one (9m). Yield 33%; mp 162-164 °C. ^1^H NMR (60 MHz, DMSO-d_6_) δ (ppm) δ (ppm): 3.14 (s, 8H), 3.81 (d, J = 1.2 Hz, 2H), 6.56 (s, 1H), 7.16-7.38 (m, 1H), 7.46 (d, J = 2.2 Hz, 1H), 7.68 (dd, J = 4.2, 2.2 Hz, 1H). ^13^C NMR (100 MHz, CDCl_3_) δ (ppm): 51.34, 51.37, 57.45, 118.77, 118.88, 119.36, 123.99, 129.81, 132.11, 149.62, 152.27, 159.86.

6-chloro-4-[(4-phenylpiperidin-1-yl)methyl]-2H-chromen-2-one (9n). Yield 57%; mp 163-164 °C. ^1^H NMR (300 MHz, CDCl_3_) δ (ppm) δ (ppm) δ (ppm): 1.78 (dd, J = 10.1, 3.6 Hz, 4H), 2.20 (td, J = 11.1, 3.5 Hz, 2H), 2.48 (tt, J = 10.1, 5.0 Hz, 1H), 2.99 (d, J = 11.5 Hz, 2H), 3.61 (d, J = 1.2 Hz, 2H), 6.53 (s, 1H), 7.11-7.16 (m, 2H), 7.16-7.21 (m, 2H), 7.22-7.27 (m, 2H), 7.29 (dd, J = 8.5, 2.0 Hz, 1H), 7.56 (d, J = 1.9 Hz, 1H). ^13^C NMR (75 MHz, CDCl_3_) δ (ppm): 35.12, 43.91, 56.39, 60.59, 115.93, 118.44, 120.29, 124.80, 127.80, 128.39, 130.03, 133.06, 141.61, 147.63, 153.60, 154.10, 163.04.

6-methyl-4-[(phenylamino)methyl]-2H-chromen-2-one (10a). Yield 61%; mp 164-165 °C; HRMS 30eV (Q-TOF) (m/z): calcd. for C_17_H_15_NO_2_ [M+H]+: 266.1136, found 266.1180. ^1^H NMR (300 MHz, CDCl_3_) δ (ppm): 2.46 (s, 3H), 4.55 (d, J = 1.2 Hz, 2H), 6.55 (s, 1H), 6.63 (d, J = 7.7 Hz, 2H), 6.80 (t, J = 7.4 Hz, 1H), 7.21 (t, J = 7.9 Hz, 2H), 7.27-7.33 (m, 1H), 7.40 (d, J = 8.4 Hz, 1H), 7.45 (s, 1H). ^13^C NMR (75 MHz, CDCl_3_) δ (ppm): 21.07, 44.38, 112.83, 113.20, 117.13, 117.88, 118.57, 123.21, 129.48, 132.85, 134.03, 146.80, 151.89, 152.18, 161.09.

6-methyl- 4-{[(4-methylphenyl)amino]methyl}-2H-chromen-2-one (10b). Yield 43%; mp 188-189 °C; HRMS 30eV (Q-TOF) (m/z): calcd. for C_18_H_17_NO_2_ [M+H]+: 280.1293, found 280.1338. ^1^H NMR (400 MHz, DMSO-d_6_) δ (ppm): 2.27 (s, 3H), 2.46 (s, 3H), 4.51 (s, 2H), 6.56 – 6.52 (m, 3H), 7.01 (d, J = 8.5 Hz, 2H), 7.28 (s, 1H), 7.38 (dd, J = 8.5, J =1.7 Hz, 1H), 7.45 (s, 1H). ^13^C NMR (100 MHz, CDCl_3_) δ (ppm): 20.37, 21.14, 44.61, 112.91, 113.08, 117.02, 117.88, 123.26, 127.66, 129.91, 132.77, 133.99, 144.56, 151.82, 152.55, 161.17.

4-{[(4-chlorophenyl)amino]methyl}-6-methyl-2H-chromen-2-one (10c). Yield 41%; mp 180-181 °C; HRMS 30eV (Q-TOF) (m/z): calcd. for C_17_H_14_ClNO_2_ [M+H]+: 300.0747, found 300.0798. ^1^H NMR (300 MHz, CDCl_3_) δ (ppm): 0.89 (s, 3H), 4.13 (s, 2H), 6.07 (s, 1H), 6.12 (d, J = 8.4 Hz, 2H), 6.72 (d, J = 8.4 Hz, 2H), 6.89 (d, J = 9.1 Hz, 1H), 6.99 (d, J = 7.7 Hz, 1H), 7.02 (s, 1H). ^13^C NMR (75 MHz, CDCl_3_) δ (ppm): 27.88, 43.83, 112.37, 113.35, 116.67, 117.17, 121.42, 122.55, 128.72, 131.29, 139.98, 144.87, 151.42, 160.51.

4-{[(4-bromophenyl)amino]methyl}-6-methyl-2H-chromen-2-one (10d). Yield 37%; mp 178-179 °C; HRMS 30eV (Q-TOF) (m/z): calcd. for C_17_H_14_BrNO_2_ [M+H]+: 344.0241, found 344.0276. ^1^H NMR (300 MHz, CDCl_3_) δ (ppm): 1.11 (s, 3H), 4.35 (s, 2H), 6.31 (m, 3H), 7.05-7.14 (m, 3H), 7.23 (dd, J = 4.4, 2.5 Hz, 2H). ^13^C NMR (75 MHz, CDCl_3_) δ (ppm): 27.02, 42.83, 108.68, 111.45, 112.92, 115.80, 116.25, 120.51, 130.42, 130.71, 139.09, 144.36, 150.41, 150.51, 159.62.

4-{[(4-methoxyphenyl)amino]methyl}-6-methyl-2H-chromen-2-one (10e). Yield 69%; mp 169-170 °C. HRMS 30eV (Q-TOF) (m/z): calcd. for C_18_H_17_NO_3_ [M+H]+: 296.1242, found 296.1294. ^1^H NMR (300 MHz, CDCl_3_) δ (ppm): 2.45 (s, 3H), 3.76 (s, 3H), 4.50 (s, 2H), 6.55 (s, 1H), 6.57 (d, J = 8.9 Hz, 2H), 6.80 (d, J = 8.9, 2H), 7.29 (m, 1H), 7.38 (dd, J = 8.5, 1.4 Hz, 1H), 7.44 (s, 1H). ^13^C NMR (75 MHz, CDCl_3_) δ (ppm): 21.08, 45.19, 55.84, 113.17, 114.08, 115.05, 117.05, 117.90, 123.27, 132.80, 133.99, 140.97, 151.85, 152.57, 152.73, 161.15.

4-{[(3,5-dimethylphenyl)amino]methyl}-6-methyl-2H-chromen-2-one (10f). Yield 59%; mp 179-180 °C; HRMS 30eV (Q-TOF) (m/z): calcd. for C_19_H_19_NO_2_ [M+H]+: 294.1449, found 294.1500. ^1^H NMR (300 MHz, CDCl_3_) δ (ppm): 2.22 (s, 6H), 2.44 (s, 3H), 4.49 (s, 2H), 6.23 (s, 2H), 6.43 (s, 1H), 6.51 (s, 1H), 7.26 (d, J = 7.7 Hz, 1H), 7.33-7.38 (m, 1H), 7.42 (s, 1H). ^13^C NMR (75 MHz, CDCl_3_) δ (ppm): 21.05, 21.48, 44.42, 110.73, 113.13, 117.05, 117.89, 120.54, 123.28, 133.77, 139.18, 146.97, 151.85, 152.51, 161.14.

6-methyl-4-{[methyl(phenyl)amino]methyl}-2H-chromen-2-one (10g). Yield 67%; mp 162-164 °C. HRMS 30eV (Q-TOF) (m/z): calcd. for C_18_H_17_NO_2_ [M+H]+: 280.1293, found 280.1333. ^1^H NMR (300 MHz, CDCl_3_) δ (ppm): 2.43 (s, 3H), 3.10 (s, 3H), 4.65 (d, J = 1.4 Hz, 2H), 6.31 (s, 1H), 6.69 (d, J = 8.0 Hz, 2H), 6.80 (t, J = 7.3 Hz, 1H), 7.21-7.32 (m, 3H), 7.40 (m, 2H). ^13^C NMR (75 MHz, CDCl_3_) δ (ppm): 21.06, 38.97, 53.70, 112.14, 112.65, 117.13, 117.56, 117.84, 123.07, 129.39, 132.84, 133.99, 148.62, 151.32, 151.96, 161.09.

4-{[(4-chlorophenyl)(methyl)amino]methyl}-6-methyl-2H-chromen-2-one (10h). Yield 49%; mp 173-174 °C. HRMS 30eV (Q-TOF) (m/z): calcd. for C_18_H_16_ClNO_2_ [M+H]+: 314.0903, found 314.0953. ^1^H NMR (300 MHz, CDCl_3_) δ (ppm): 2.43 (s, 3H), 3.09 (s, 3H), 4.63 (d, J = 1.0 Hz, 1H), 6.24 (s, 1H), 6.57 (d, J = 9.0 Hz, 2H), 7.16 (d, J = 9.0 Hz, 2H), 7.28 (d, J = 8.4 Hz, 1H), 7.36-7.43 (m, 2H). ^13^C NMR (75 MHz, CDCl_3_) δ (ppm): 21.05, 39.17, 53.69, 112.58, 113.23, 117.19, 117.67, 122.47, 122.99, 129.18, 132.97, 134.07, 147.17, 150.77, 151.96, 160.95.

6-methyl-4-(piperidin-1-ylmethyl)-2H-chromen-2-one (10i). Yield 55%; mp 161-163 °C. HRMS 30eV (Q-TOF) (m/z): calcd. for C_16_H_19_NO_2_ [M+H]+: 258.1449, found 258.1499. ^1^H NMR (400 MHz, CDCl_3_) δ (ppm): 1.48 (m, 2H), 1.62 (m, 4H), 2.41 (s, 3H), 2,49 (m, 4H), 3.58 (d, J =1.3 Hz, 2H), 6.56 (s, 1H), 7.22 (d, J = 8.4 Hz, 1H), 7.32 (dd, J = 8.4 Hz, J =1.8 Hz, 1H), 7.56 (d, J = 1.2 Hz, 1H). ^13^C NMR (100 MHz, CDCl_3_) δ (ppm): 21.23, 24.27, 26.19, 55.25, 59.47, 114.51, 116.91, 118.88, 124.49, 132.85, 133.78, 152.03, 152.69, 161.65.

6-methyl-4-(piperazin-1-ylmethyl)-2H-chromen-2-one (10j). Yield 39%; mp > 300 °C. ^1^H NMR (300 MHz, CDCl_3_) δ (ppm): 2.43 (s, 3H), 2.62-2.53 (m, 4H), 3.63 (s, 2H), 3.80-3.72 (m, 4H), 6.56 (s, 1H), 7.25 (t, J = 8.5 Hz, 1H), 7.34 (d, J = 7.0 Hz, 1H), 7.54 (s, 1H). ^13^C NMR (75 MHz, CDCl_3_) δ (ppm): 21.07, 53.91, 58.96, 66.95, 114.75, 116.86, 118.50, 124.25, 132.71, 133.71, 151.37, 151.93, 161.20.

6-methyl-4-(morpholin-4-ylmethyl)-2H-chromen-2-one (10k). Yield 53%; mp 99-100 °C. HRMS 30eV (Q-TOF) (m/z): calcd. for C_15_H_17_NO_3_ [M+H]+: 260.1242, found 260.1298. ^1^H NMR (300 MHz, CDCl_3_) δ (ppm): 2.44 (s, 3H), 2.55-2.62 (m, 4H), 3.65 (s, 2H), 3.74-3.79 (m, 4H), 6.58 (s, 1H), 7.25 (d, J = 8.4 Hz, 1H), 7.34 (d, J = 8.3 Hz, 1H), 7.56 (s, 1H). ^13^C NMR (75 MHz, CDCl_3_) δ (ppm): 21.08, 53.92, 58.96, 66.95, 114.70, 116.86, 118.46, 124.26, 132.72, 133.74, 151.41, 151.93, 161.20.

6-methyl-4-(thiomorpholin-4-ylmethyl)-2H-chromen-2-one (10l). Yield 49%; mp 121-122 °C. HRMS 30eV (Q-TOF) (m/z): calcd. for C_15_H_17_NO_2_S [M+H]+: 276.1014, found 276.1066. ^1^H NMR (300 MHz, CDCl_3_) δ (ppm): 2.42 (s, 3H), 2.69-2.76 (m, 4H), 2.79-2.86 (m, 4H), 3.64 (d, J = 1.2 Hz, 2H), 6.54 (s, 1H), 7.25 (t, J = 8.7 Hz, 1H), 7.34 (dd, J = 8.4, 1.5 Hz, 1H), 7.51 (s, 1H). ^13^C NMR (75 MHz, CDCl_3_) δ (ppm): 21.08, 28.09, 55.43, 59.25, 114.49, 116.87, 118.46, 124.15, 132.70, 133.72, 151.74, 151.91, 161.23.

4-[(1,1-dioxidothiomorpholin-4-yl)methyl]-6-methyl-2H-chromen-2-one (10m). Yield 43%; mp 170-172 °C. HRMS 30eV (Q-TOF) (m/z): calcd. for C_15_H_17_NO_4_S [M+H]+: 308.0912, found 308.0875. ^1^H NMR (60 MHz, DMSO-d_6_) δ (ppm): 2.44 (s, 3H), 3.16 (s, 8H), 3.89 (d, J = 1.3 Hz, 2H), 6.54 (s, 1H), 7.30 (d, J = 1.9, 2H), 7.53 (s, 1H). ^13^C NMR (15 MHz, DMSO-d_6_) δ (ppm): 20.11-21.16, 44.80, 51.24, 53.33, 56.57-56.68, 114.35-114.52, 116.68-116.91, 117.61-117.72, 124.20-124.38, 132.93-133.23, 133.82-134.00, 146.93-147.63, 150.98-151.48.

6-methyl-4-[(4-phenylpiperidin-1-yl)methyl]-2H-chromen-2-one (10n). Yield 65%; mp 106-107 °C. HRMS 30eV (Q-TOF) (m/z): calcd. for C_22_H_23_NO_2_ [M+H]+: 334.1762, found 334.1816. ^1^H NMR (300 MHz, CDCl_3_) δ (ppm): 2.15-2.33 (m, 4H), 2.46 (s, 3H), 2.50-2.69 (m, 2H), 2.78 (t, J = 11.4 Hz, 1H), 3.08 (d, J = 11.3 Hz, 2H), 3.69 (s, 2H), 6.63 (s, 1H), 7.29 (m, 7H), 7.60 (s, 1H). ^13^C NMR (75 MHz, CDCl_3_) δ (ppm): 21.11, 33.53, 34.38, 42.38, 43.02, 47.08, 54.89, 58.98, 114.47, 116.83, 118.70, 124.33, 126.14, 126.25, 126.86, 128.49, 132.57, 133.66, 146.07,151.93, 152.40, 161.46.

6-ethyl-4-[(phenylamino)methyl]-2H-chromen-2-one (11a). Yield 59%; mp 91-93 °C; HRMS 30eV (Q-TOF) (m/z): calcd. for C_18_H_17_NO_2_ [M+H]+: 280.1293, found 280.1338. ^1^H NMR (300 MHz, CDCl_3_) δ (ppm): 1.27 (t, J = 7.6 Hz, 3H), 2.71 (q, J = 7.6 Hz, 2H), 4.48 (d, J = 1.3 Hz, 2H), 6.48 (s, 1H), 6.57 (d, J = 7.7 Hz, 2H), 6.66 (dd, J = 8.4, 1.0Hz, 1H), 6.73 (t, J = 7.3 Hz, 1H), 7.19 – 7.15 (m, 1H), 7.24 (d, J = 8.5 Hz, 1H), 7.36 (dd, J = 8.5, 1.9Hz, 1H), 7.40 (d, J = 1.7 Hz, 1H). ^13^C NMR (75 MHz, CDCl_3_) δ (ppm): 15.88, 28.51, 44.34, 112.84, 115.20, 117.15, 117.95, 118.35, 118.54, 122.20, 129.33, 129.47, 131.78, 140.54, 146.47, 147.03, 151.97, 152.78, 161.37.

6-ethyl-4-{[(4-methylphenyl)amino]methyl}-2H-chromen-2-one (11b). Yield 55%; mp 171-172 °C. HRMS 30eV (Q-TOF) (m/z): calcd. for C_19_H_19_NO_2_ [M+H]+: 294.1416, found 294.1407. ^1^H NMR (400 MHz, DMSO-d_6_) δ (ppm): 1.31 (t, J = 7.8, 3H), 2.24 (s, 3H), 2.77 (q, J = 7.5 Hz, 2H), 4.51 (d, J = 1.3 Hz, 2H), 6.54 – 6,48 (m, 3H), 6.99 (d, J = 8.1 Hz, 2H), 7.28 (d, J = 8.5 Hz, 1H), 7.39 (dd, J = 8.5, 1.9 Hz, 1H), 7.43 (d, J = 1.5 Hz, 1H). ^13^C NMR (100 MHz, CDCl_3_) δ (ppm): 15.79, 20.37, 28.45, 44.67, 112.93, 113.02, 117.16, 117.93, 122.10, 127.68, 129.91, 131.67, 140.41, 144.58, 151.97, 152.65, 161.20.

4-{[(4-chlorophenyl)amino]methyl}-6-ethyl-2H-chromen-2-one (11c). Yield 62%; mp 171-173 °C. HRMS 30eV (Q-TOF) (m/z): calcd. for C_18_H_16_ClNO_2_ [M+H]+: 314.0903, found 314.0965. ^1^H NMR (300 MHz, CDCl_3_) δ (ppm): 1.28 (t, J = 7,7 Hz, 3H), 2.73 (q, J = 7,6 Hz, 2H), 4.52 (s, 2H), 6.46 (s, 1H), 6.52 (d, J = 8,4 Hz, 2H), 7.11 (d, J = 8.4 Hz, 2H), 7.28 (d, J = 9.0 Hz, 2H), 7.40 (d, J = 6.9 Hz, 2H). ^13^C NMR (75 MHz, CDCl_3_) δ (ppm): 15.86, 28.63, 44.58, 113.12, 114.10, 117.36, 117.92, 122.17, 123.29, 129.47, 132.04, 140.73, 145.62, 151.17, 161.26.

4-{[(4-bromophenyl)amino]methyl}-6-ethyl-2H-chromen-2-one (11d). Yield 51%; mp 184-186 °C. HRMS 30eV (Q-TOF) (m/z): calcd. for C_18_H_16_BrNO_2_ [M+H]+: 358.0398, found 358.0439. ^1^H NMR (300 MHz, CDCl_3_) δ (ppm): 1.28 (t, J = 7.7 Hz, 3H), 2.73 (q, J = 7.6 Hz, 2H), 4.52 (s, 2H), 6.46 (s, 1H), 6.52 (d, J = 8.4 Hz, 2H), 7.11 (d, J = 8.4 Hz, 2H), 7.28 (m, 1H), 7.45 – 7.36 (m, 2H). ^13^C NMR (75 MHz, CDCl_3_) δ (ppm): 15.92, 28.63, 44.58, 113.12, 114.10, 117.42, 117.92, 122.17, 123.29, 129.47, 132.04, 140.73, 145.62, 152.17, 161.26.

6-ethyl-4-{[(4-methoxyphenyl)amino]methyl}-2H-chromen-2-one (11e). Yield 67%; mp 151-152 °C. HRMS 30eV (Q-TOF) (m/z): calcd. for C_19_H_19_NO_3_ [M+H]+: 310.1398, found 310,1365. ^1^H NMR (400 MHz, CDCl_3_) δ (ppm): 1.27 (t, J = 7.6 Hz, 3H), 2.72 (q, J = 7.6 Hz, 2H), 3.74 (s, 3H), 4.49 (d, J = 1.4 Hz, 2H), 6.52 (s, 1H), 6.54 – 6.58 (m, 2H), 6.75 – 6.81 (m, 2H), 7.28 (d, J = 8.5 Hz, 1H), 7.39 (dd, J = 8.5, J = 1.9 Hz, 1H), 7.43 (d, J = 1.8 Hz, 1H). ^13^C NMR (100 MHz, CDCl_3_) δ (ppm): 15.94, 28.61, 45.39, 55.97, 113.24, 114.26, 115.23, 117.23, 118.11, 122.29, 131.84, 140.58, 141.19, 152.15, 152.89, 161.36.

4-{[(3,5-dimethylphenyl)amino]methyl}-6-ethyl-2H-chromen-2-one (11f). Yield 64%; mp 176-178 °C. HRMS 30eV (Q-TOF) (m/z): calcd. for C_20_H_21_NO_2_ [M+H]+: 308.1606, found 308.1659. ^1^H NMR (300 MHz, CDCl_3_) δ (ppm): 1.33 (t, J = 7.6 Hz, 3H), 2.26 (s, 6H), 2.77 (q, J = 7.6 Hz, 2H), 4.53 (d, J = 1.3 Hz, 2H), 6.27 (s, 2H), 6.46 (s, 1H), 6.54 (s, 1H), 7.30 (d, J = 8.3 Hz, 1H), 7.42 (dd, J= 8.5, 1.9 Hz, 1H), 7.47 (d, J = 1.7 Hz, 1H). ^13^C NMR (75 MHz, CDCl_3_) δ (ppm): 15.89, 21.58, 28.54, 44.47, 110.80, 112.97, 117.16, 118.04, 120.47, 122.26, 131.71, 139.18, 140.50, 147.19, 151.01, 152.95, 161.32.

4-{[(4-chlorophenyl)(methyl)amino]methyl}-6-ethyl-2H-chromen-2-one (11h). Yield 58%; mp 158-160 °C. HRMS 30eV (Q-TOF) (m/z): calcd. for C_19_H_18_ClNO_2_ [M+H]+: 328.1060, found 313.0870. ^1^H NMR (400 MHz, DMSO-d_6_) δ (ppm): 1.28 (t, J = 7.56 Hz, 3H), 2.73 (q, J = 7.5 Hz, 2H), 3.09 (s, 3H), 4.65 (d, J = 1.6 Hz, 2H), 6.24 (t, J = 1.6 Hz, 1H), 6.59 – 6.54 (m, 2H), 7.18 – 7.12 (m, 2H), 7.30 (d, J = 8.5 Hz, 1H); 7.36 (d, J = 1.9 Hz, 1H), 7.41 (dd, J = 8.5, 2.0 Hz, 1H). ^13^C NMR (100 MHz, CDCl_3_) δ (ppm): 15.89, 28.61, 39.30, 53.88, 112.68, 113.42, 117.45, 117.88, 121.99, 122.60, 129.33, 132.06, 140.66, 147.37, 151.05, 152.25, 161.13.

6-ethyl-4-{[methyl(phenyl)amino]methyl}-2H-chromen-2-one (11g). Yield 41%; mp 141-147 °C. HRMS 30eV (Q-TOF) (m/z): calcd. for C_19_H_19_NO_2_ [M+H]+: 294.1449, found 294.1497. ^1^H NMR (300 MHz, CDCl_3_) δ (ppm): 1.28 (t, J = 7.6 Hz, 3H), 2.72 (q, J = 7.6 Hz, 2H), 3.09 (s, 3H), 4.66 (d, J = 1.5 Hz, 2H), 6.29 (s, 1H), 6.66 (d, J = 8.0 Hz, 2H), 6.75 (t, J = 7.3 Hz, 1H), 7.19-7.24 (m, 2H), 7.26-7.30 (m, 1H), 7.43 – 7.36 (m, 2H). ^13^C NMR (75 MHz, CDCl_3_) δ (ppm): 15.85, 28.53, 39.00, 53.75, 112.20, 112.57, 117.25, 117.56, 117.95, 122.04, 129.44, 131.84, 140.50, 148.70, 151.59, 152.13, 161.18.

6-ethyl-4-(piperidin-1-ylmethyl)-2H-chromen-2-one (11i). Yield 59%; mp 96-98 °C; HRMS 30eV (Q-TOF) (m/z): calcd. for C_17_H_21_NO_2_ [M+H]+: 272.1606, found 272.1638. ^1^H NMR (60 MHz, DMSO-d_6_) δ (ppm): 1.18 (t, J = 7,6 Hz, 3H), 1.33-1.44 (m, 2H), 1.53 (dt, J = 10.9, 5.6 Hz, 4H), 2.40 (m, 4H), 2.63 (q, J = 7.6 Hz, 2H), 3.51 (d, J = 1.3 Hz, 2H), 6.47 (s, 1H), 7.16 (d, J = 8,5Hz, 1H), 7.27 (dd, J = 8.5, 2.0Hz, 1H), 7.53 (d, J =1.9 Hz, 1H); ^13^C NMR (75 MHz, CDCl_3_) δ (ppm): 15.84, 24.24, 26.20, 28.54, 55.15, 59.55, 114.32, 116.90, 118.88, 123.38, 131.53, 140.12, 152.09, 152.90, 161.66.

6-ethyl-4-(piperazin-1-ylmethyl)-2H-chromen-2-one (11j). Yield 31%; mp > 300 °C. HRMS 30eV (Q-TOF) (m/z): calcd. for C_16_H_20_N_2_O_2_ [M+H]+: 273.1558, found 273.1525.

6-ethyl-4-(morpholin-4-ylmethyl)-2H-chromen-2-one (11k). Yield 54%; mp 110-113 °C. HRMS 30eV (Q-TOF) (m/z): calcd. for C_16_H_19_NO_3_ [M+H]+: 274.1398, found 274.1433. ^1^H NMR (300 MHz, CDCl_3_) δ (ppm): 1.28 (t, J = 7,6 Hz, 3H), 2.57-2.62 (m, 4H), 2.72 (q, J = 7.6 Hz, 2H), 3.65 (s, 2H), 3.72-3.77 (m, 4H), 6.55 (s, 1H), 7.24 (d, J = 8.5 Hz, 1H), 7.37 (d, J = 8.4 Hz, 1H), 7.60 (s, 1H). ^13^C NMR (75 MHz, CDCl_3_) δ (ppm): 15.74, 28.43, 53.89, 59.03, 66.99, 114.62, 116.86, 118.53, 123.26, 131.63, 140.15, 151.61, 152.06, 161.14.

6-ethyl-4-(thiomorpholin-4-ylmethyl)-2H-chromen-2-one (11l). Yield 66%; mp 134-135 °C. HRMS 30eV (Q-TOF) (m/z): calcd. for C_16_H_19_NO_2_S [M+H]+: 290.1170, found 290.3959. ^1^H NMR (400 MHz, CDCl_3_) δ (ppm): 1.28 (t, J = 7.6 Hz, 3H), 2.77 – 2.68 (m, 4H); 2.85 – 2.78 (m, 4H), 3.66 (d, J = 1.1 Hz, 2H); 6.54 (s, 1H), 7.26 (d, J = 8.7 Hz, 1H), 7.37 (dd, J = 8.5, 1.9 Hz, 1H), 7.56 (d, J = 1.6 Hz, 1H).  ^13^C NMR (100 MHz, CDCl_3_) δ (ppm): 15.90, 28.30, 28.59, 55.56, 59.53, 114.64, 117.11, 118.66, 123.23, 131.80, 140.27, 152.03, 152.20, 161.41.

4-[(1,1-dioxidothiomorpholin-4-yl)methyl]-6-ethyl-2H-chromen-2-one (11m). Yield 38%; mp 175-177 °C. ^1^H NMR (400 MHz, CDCl_3_) δ (ppm): 1.19 (t, J = 7.6 Hz, 3H), 2.49 (m, 2H), 2.63 (q, J = 7.6 Hz, 2H), 3.57 (s, 2H), 3.62-3.68 (m, 4H), 6.55 (s, 1H), 7.24 (d, J =8.4 Hz, 1H), 7.38 (d, J =8.4 Hz, 1H), 7.60 (s, 1H). ^13^C NMR (100 MHz, CDCl_3_) δ (ppm): 15.78, 28.43, 53.89, 59.03, 66.99, 114.62, 116.86, 118.53, 123.26, 131.63, 140.15, 151.61, 152.16, 161.14.

6-ethyl-4-[(4-phenylpiperidin-1-yl)methyl]-2H-chromen-2-one (11n). Yield 53%; mp 118-120 °C. HRMS 30eV (Q-TOF) (m/z): calcd. for C_23_H_25_NO_2_ [M+H]+: 348.1919, found 348.1962. ^1^H NMR (300 MHz, CDCl_3_) δ (ppm): 1.29 (t, J = 7,6 Hz, 3H), 1.78-1.88 (m, 4H), 2.27 (td, J = 11.1, 3.5 Hz, 2H), 2.54 (tt, J = 10.1, 5.0 Hz, 1H), 2.73 (q, J = 7.6 Hz, 2H), 3.05 (d, J = 11.5 Hz, 2H), 3.67 (d, J = 1.2 Hz, 2H), 6.60 (s, 1H), 7.25 (qdd, J = 8.5, 5.1, 1.4 Hz, 6H), 7.36 (dd, J = 8.5, 2.0 Hz, 1H), 7.63 (d, J = 1.9 Hz, 1H). ^13^C NMR (75 MHz, CDCl_3_) δ (ppm): 15.78, 28.47, 32.57, 42.37, 54.84, 59.05, 114.39, 116.90, 118.74, 123.25, 126.26, 126.85, 128.49, 131.52, 140.06, 146.08, 152.05, 152.56, 161.49.


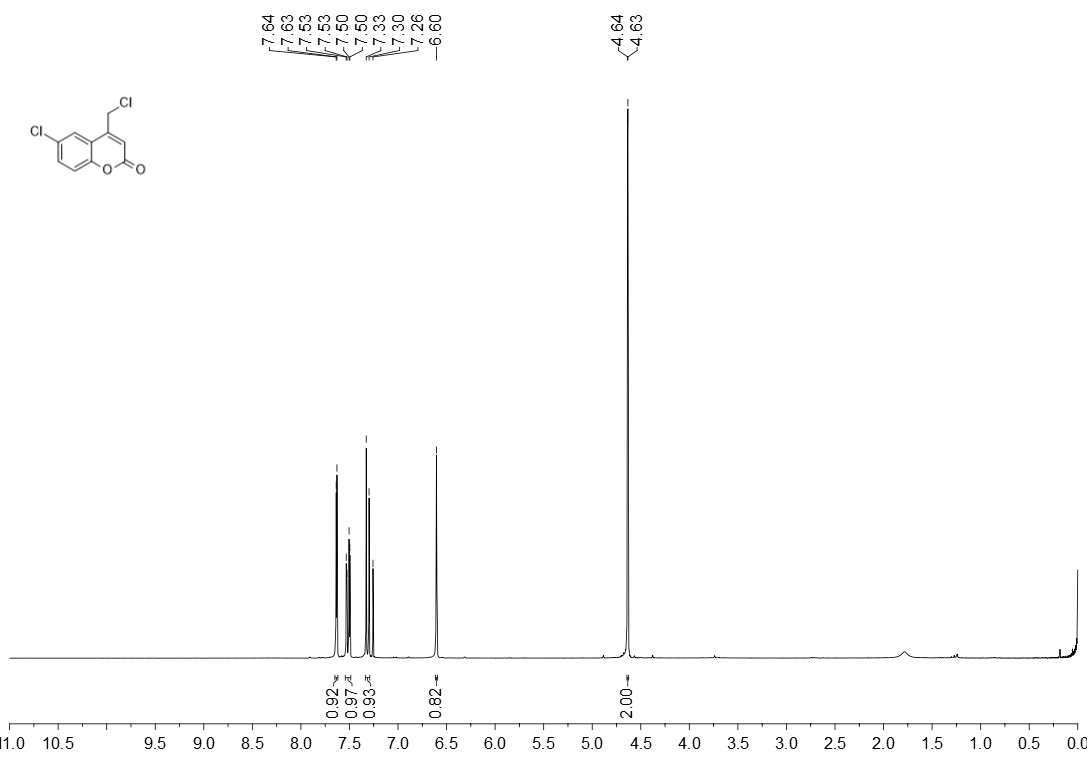


# **Figure S4**. ^1^H NMR spectrum (300 MHz, CDCl_3_) of compound **5.**


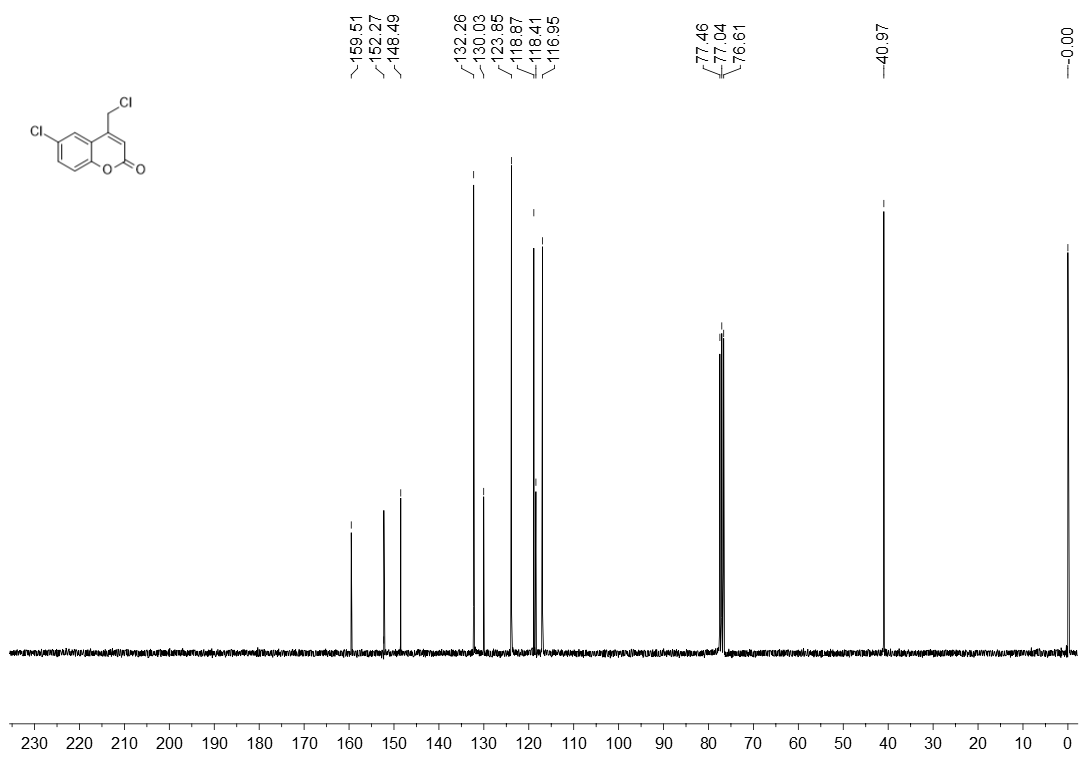


# **Figure S5.** ^13^C NMR spectrum (75 MHz, CDCl_3_) of compound **5**


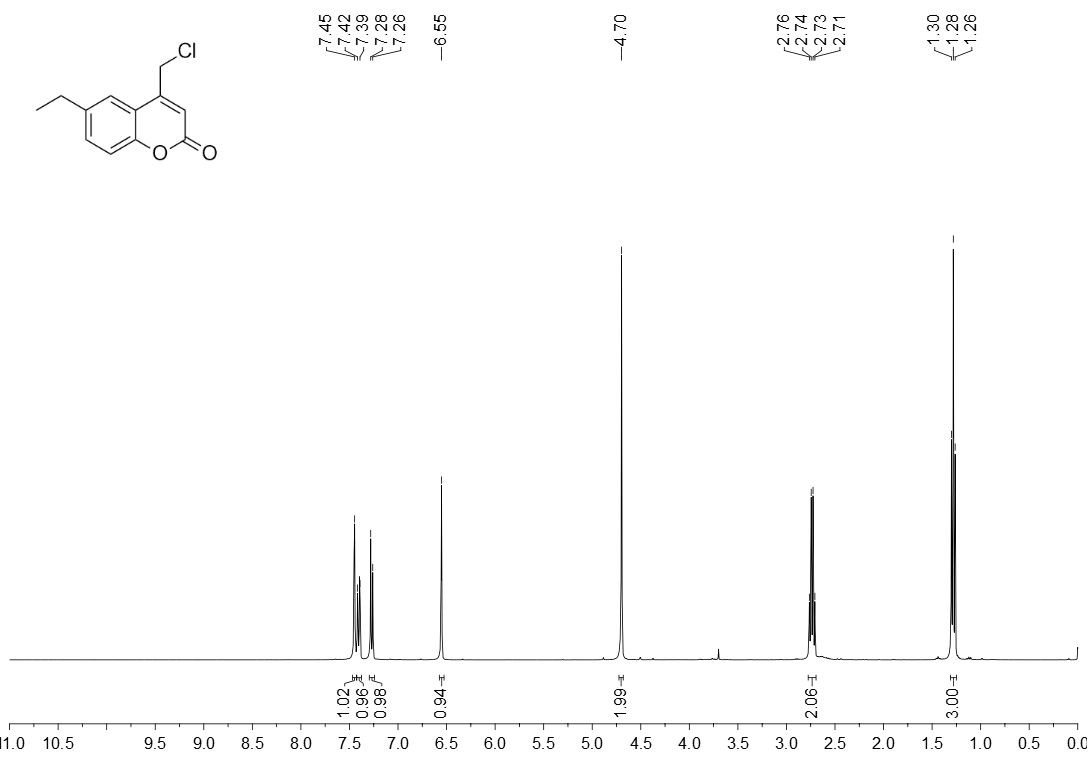


# **Figure S6**. ^1^H NMR spectrum (300 MHz, CDCl_3_) of compound **7.**


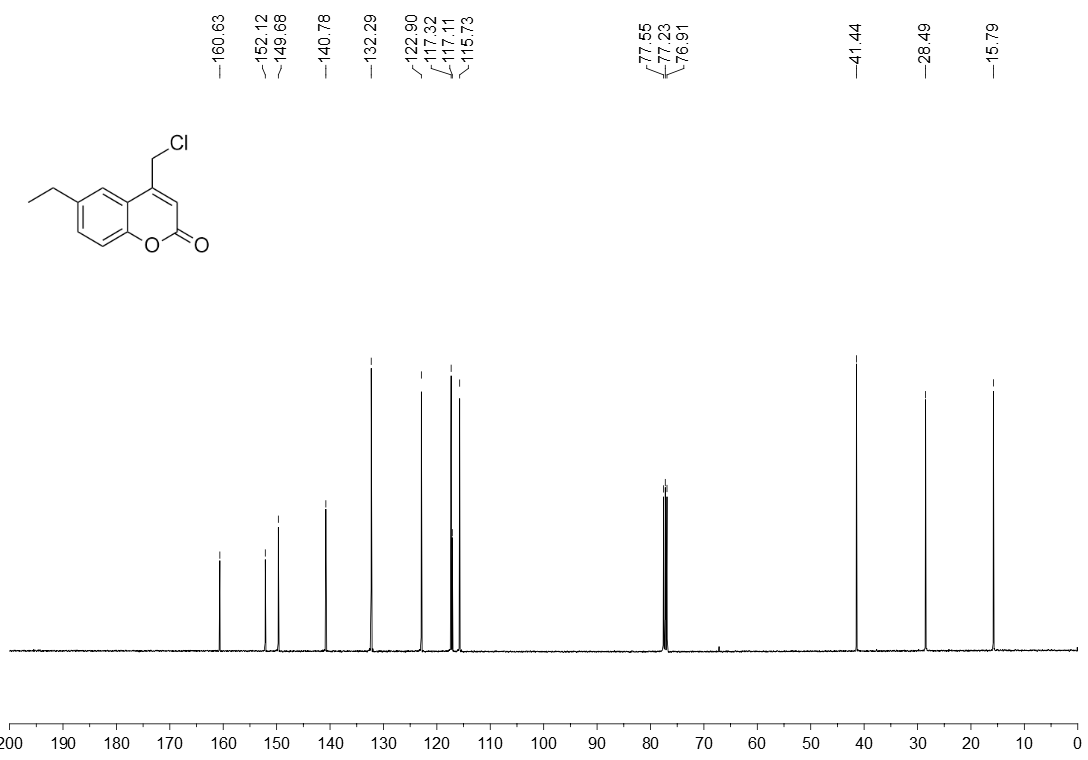


# **Figure S7.** ^13^C NMR spectrum (75 MHz, CDCl_3_) of compound **7**


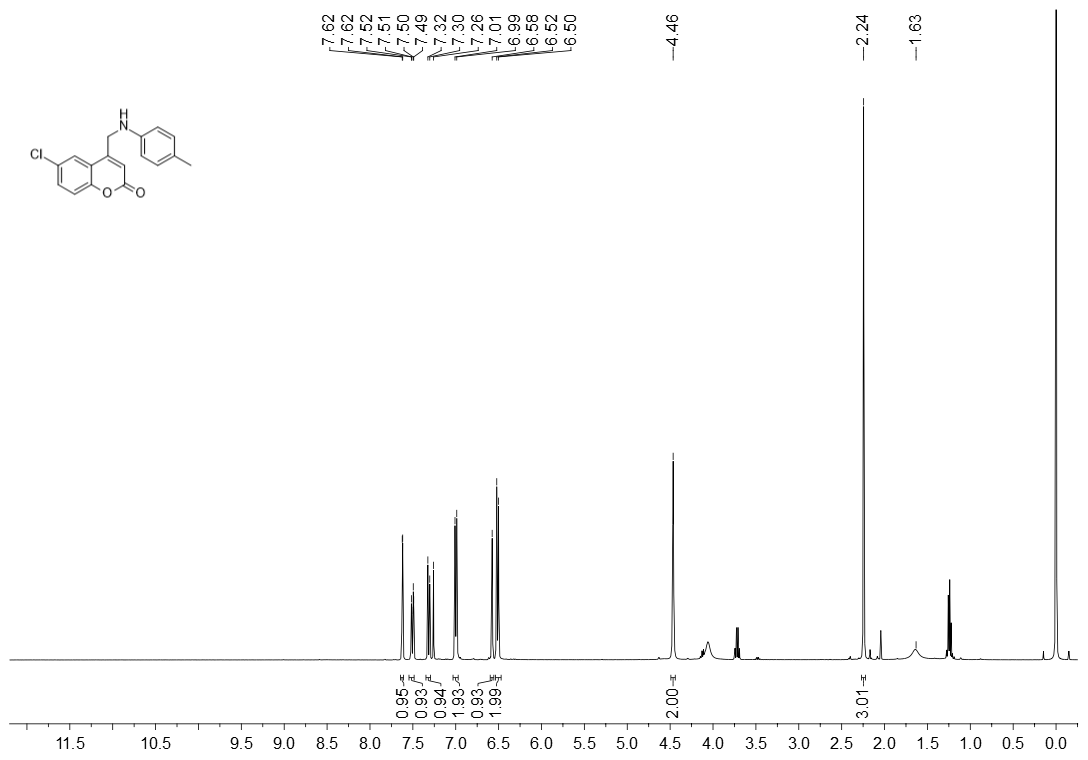


# **Figure S8**. ^1^H NMR spectrum (400 MHz, CDCl_3_) of compound **9b.**


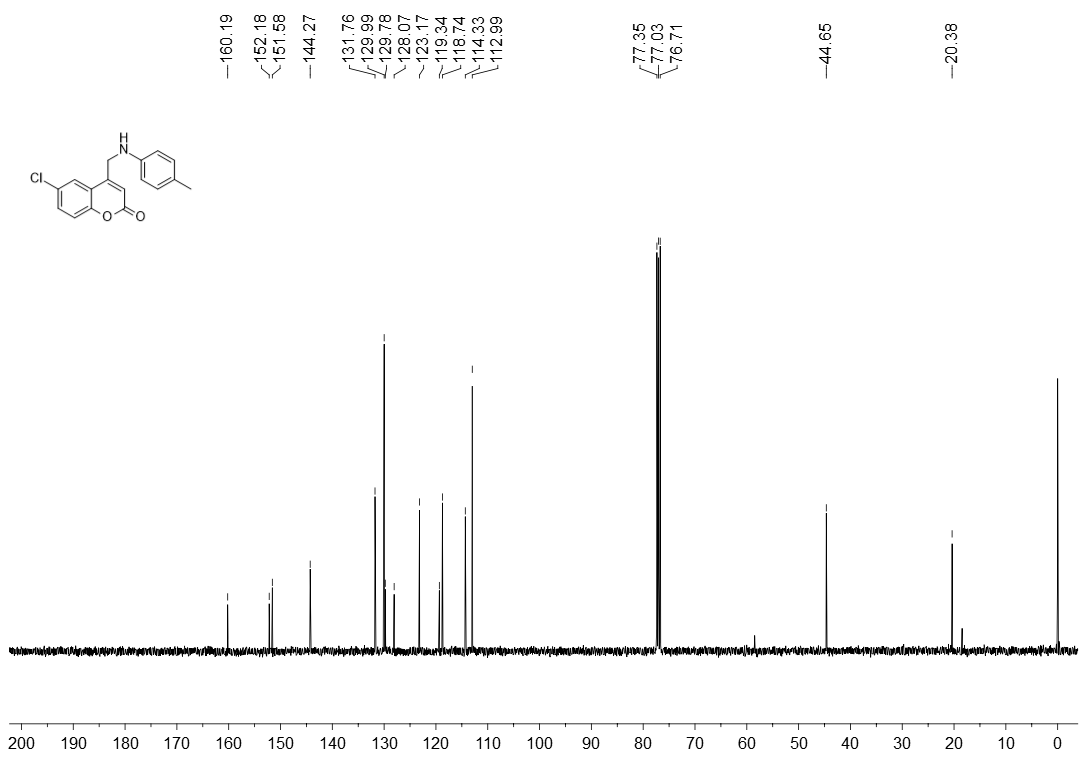


# **Figure S9.** ^13^C NMR spectrum (100 MHz, CDCl_3_) of compound **9b.**


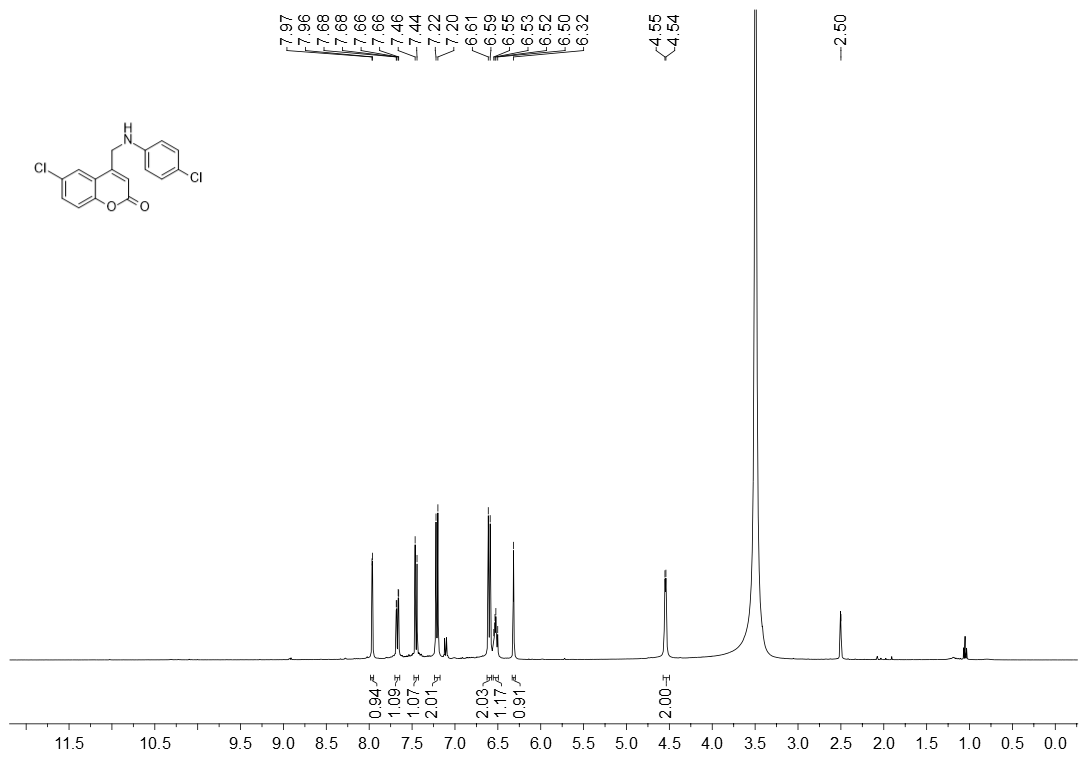


**Figure S10**. ^1^H NMR spectrum (400 MHz, DMSO-d_6_) of compound **9c.**


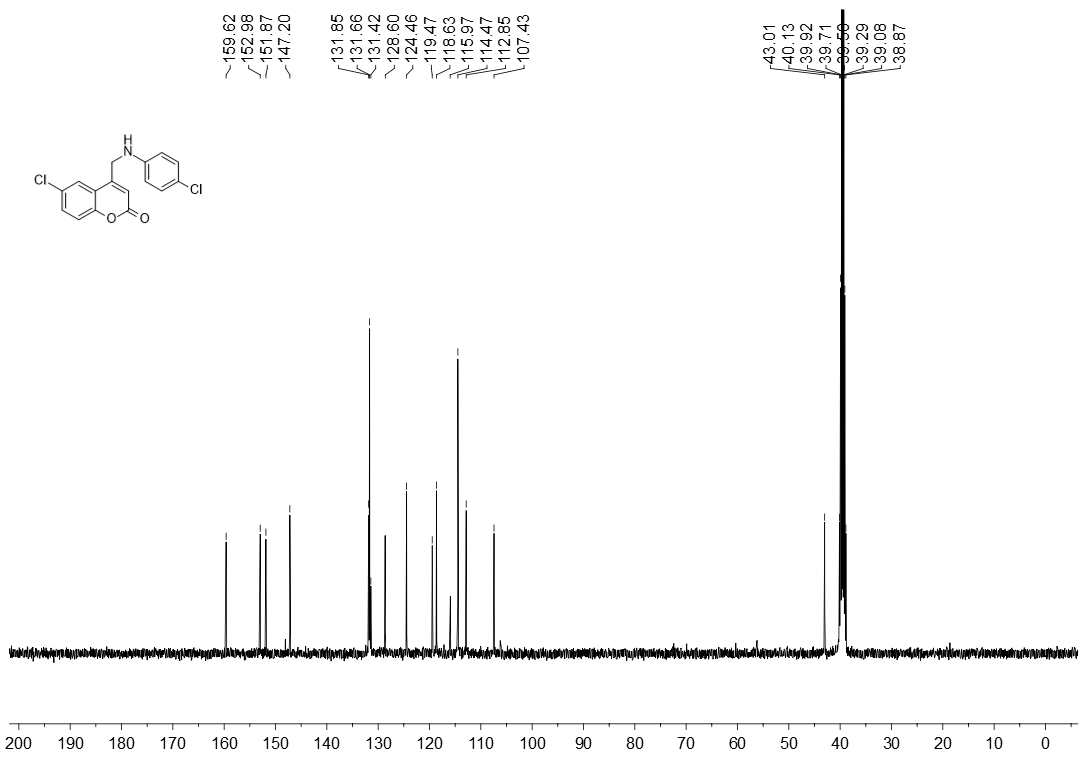


# **Figure S11.** ^13^C NMR spectrum (100 MHz, DMSO-d_6_) of compound **9c.**


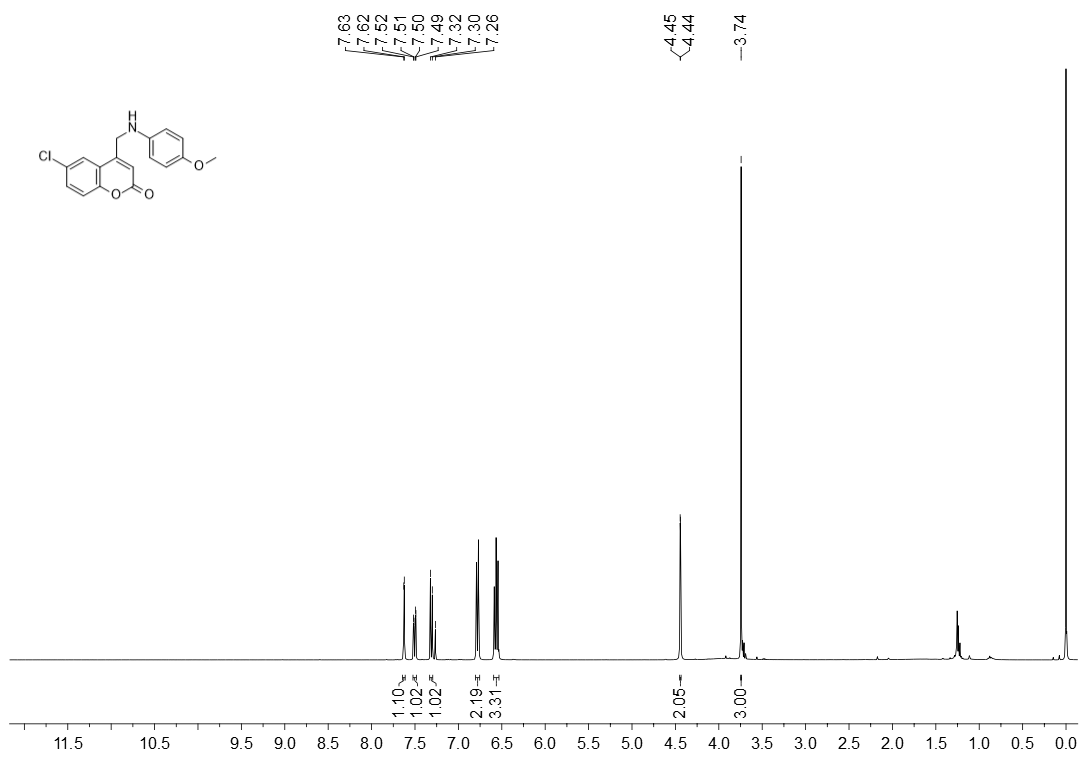


# **Figure S12**. ^1^H NMR spectrum (400 MHz, CDCl_3_) of compound **9e.**


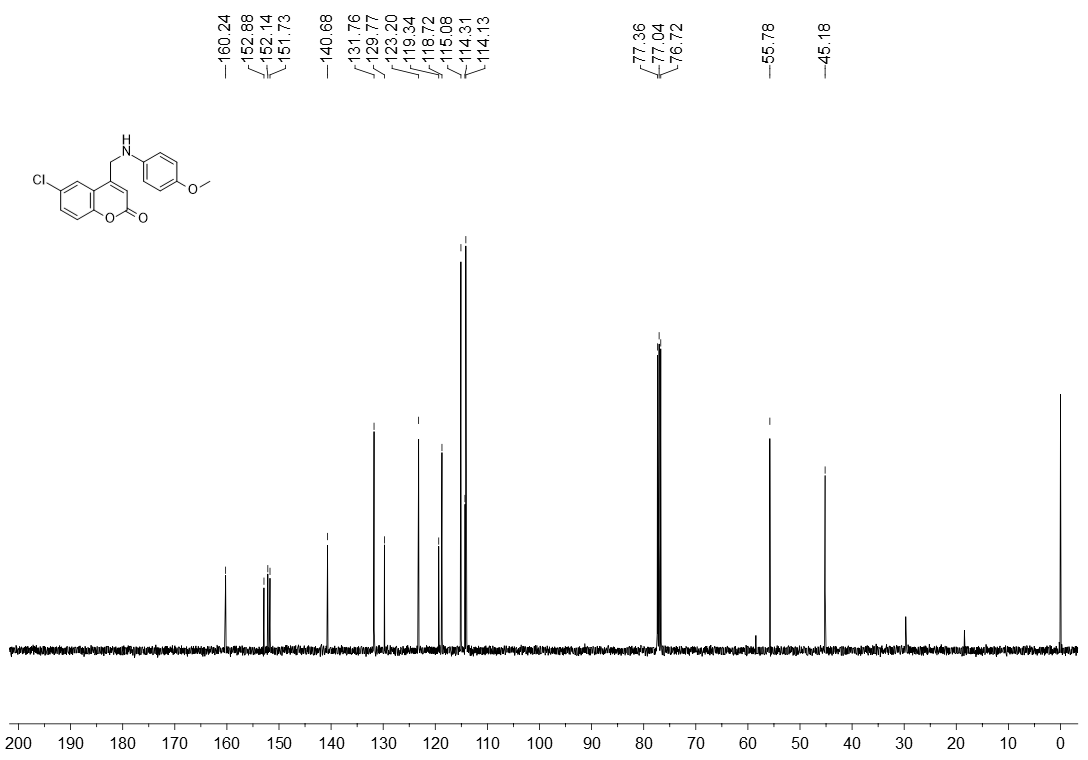


# **Figure S13**. ^13^C NMR spectrum (100 MHz, CDCl_3_) of compound **9e.**


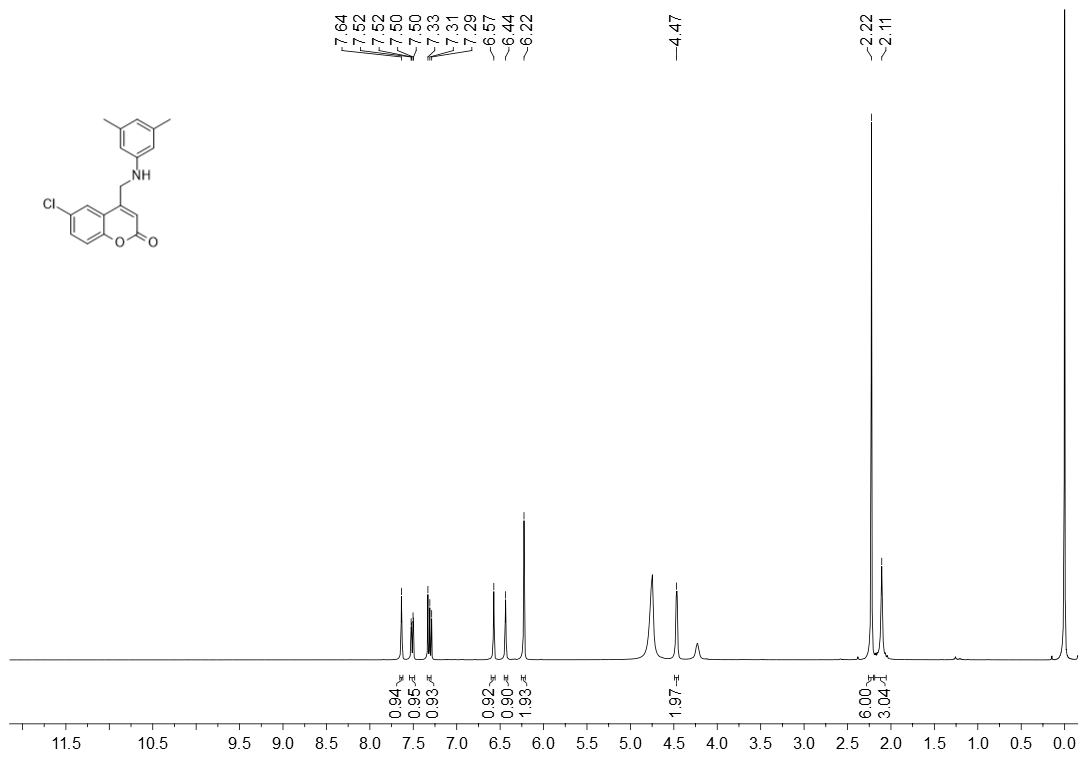


# **Figure S14**. ^1^H NMR spectrum (400 MHz, CDCl_3_) of compound **9f.**


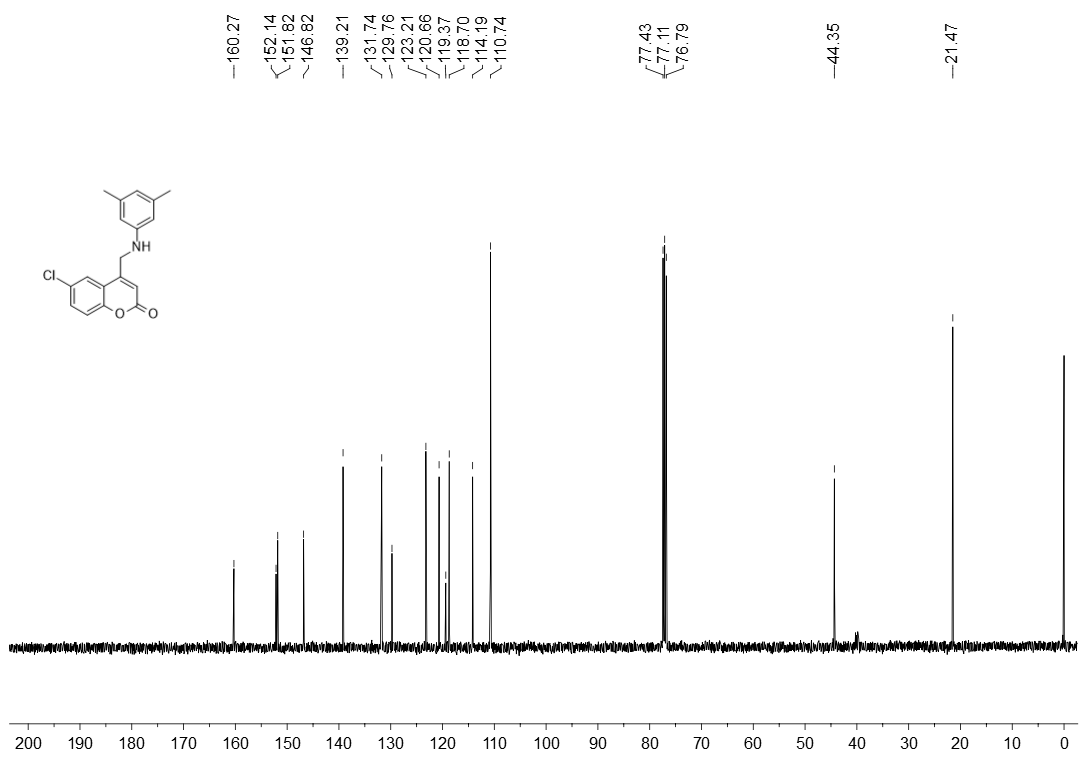


# **Figure S15**. ^13^C NMR spectrum (100 MHz, CDCl_3_) of compound **9f.**


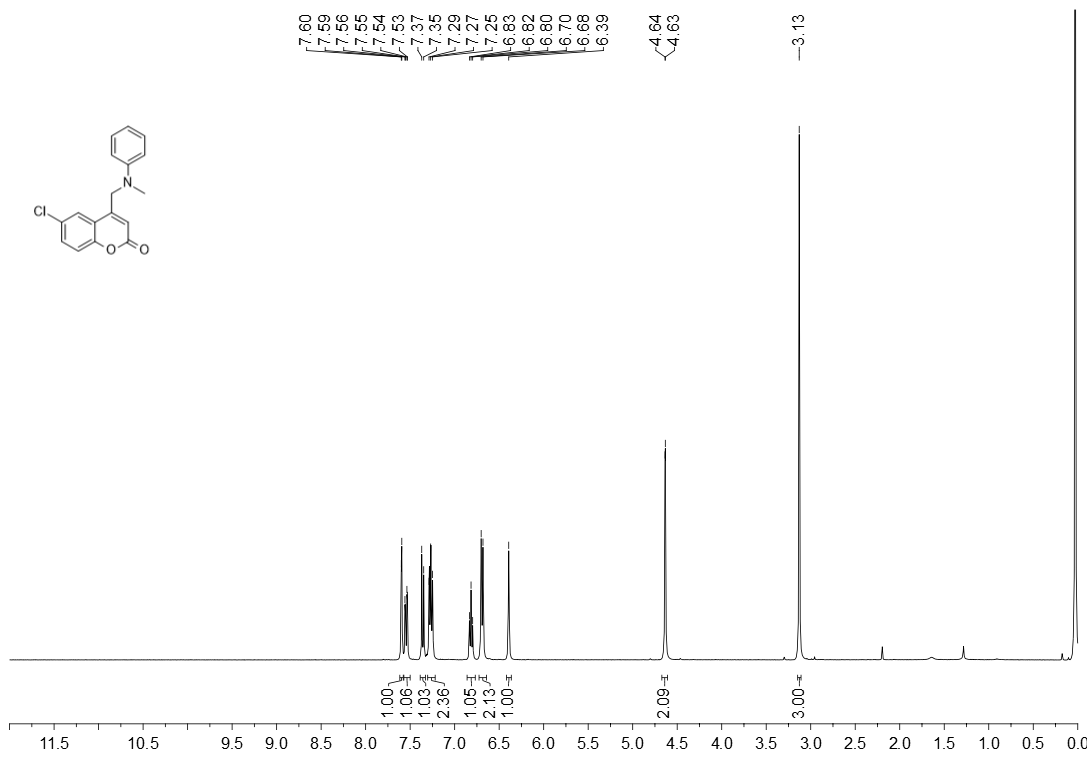


# **Figure S16**. ^1^H NMR spectrum (300 MHz, CDCl_3_) of compound **9g.**


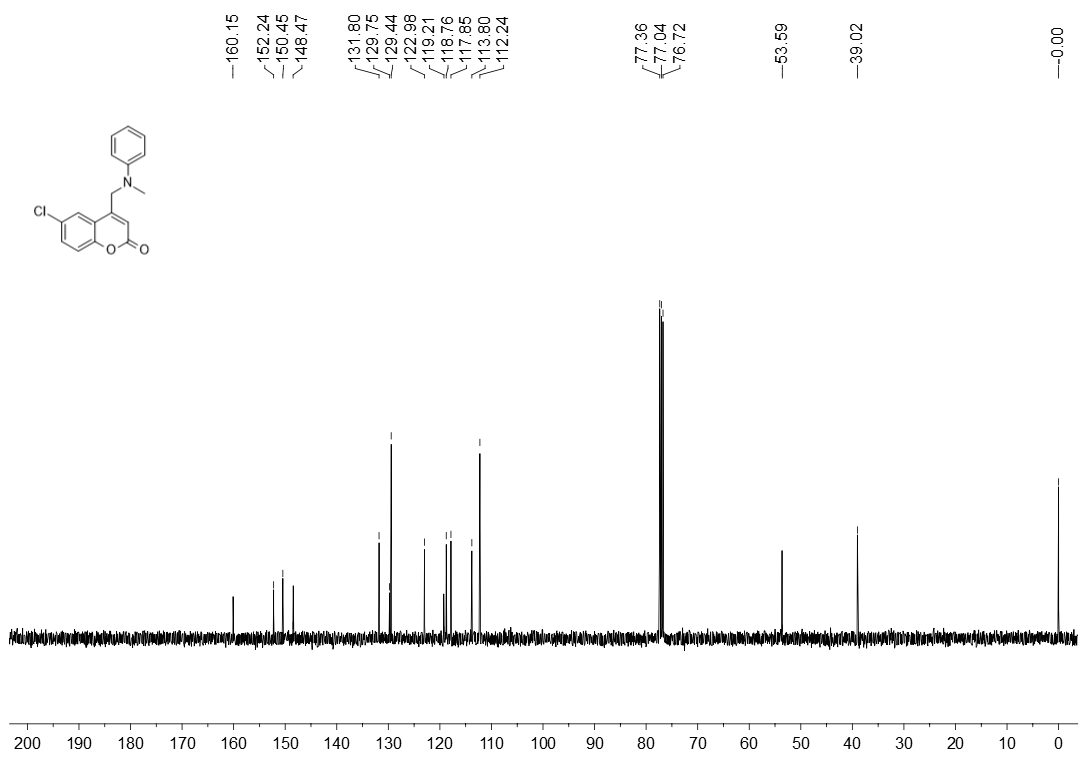


# **Figure S17**. ^13^C NMR spectrum (75 MHz, CDCl_3_) of compound **9g.**

#
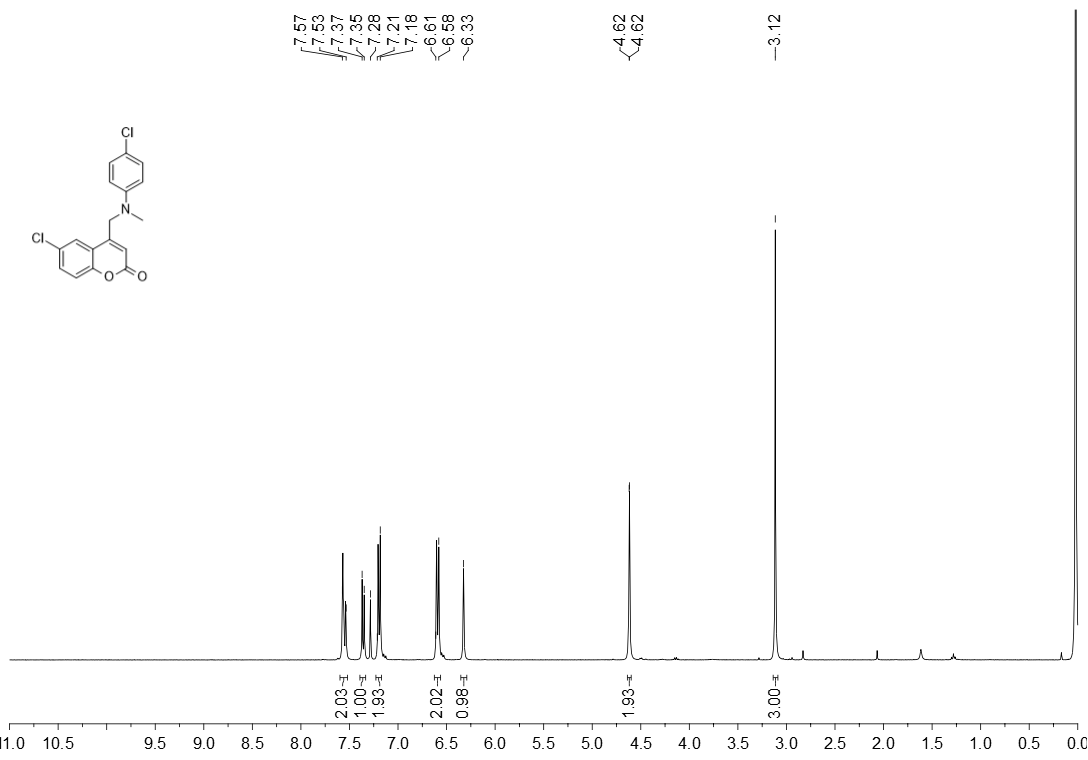


# **Figure S18**. ^1^H NMR spectrum (300 MHz, CDCl_3_) of compound **9h.**


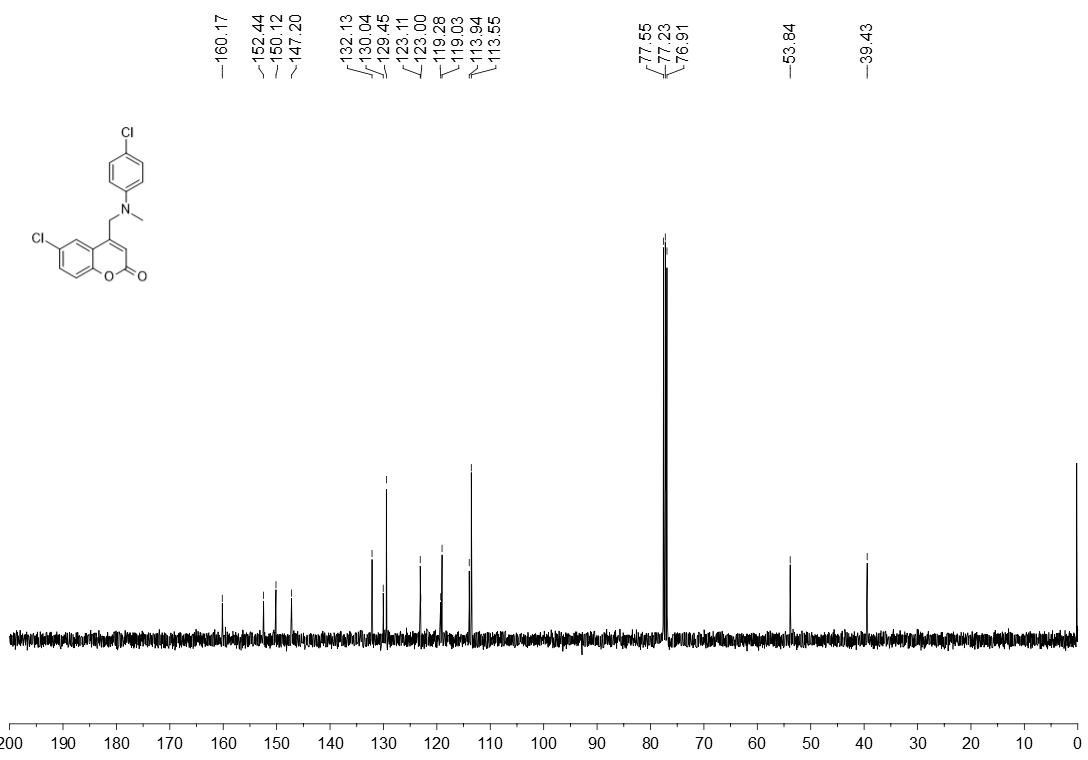


# **Figure S19**. ^13^C NMR spectrum (75 MHz, CDCl_3_) of compound **9h.**

#
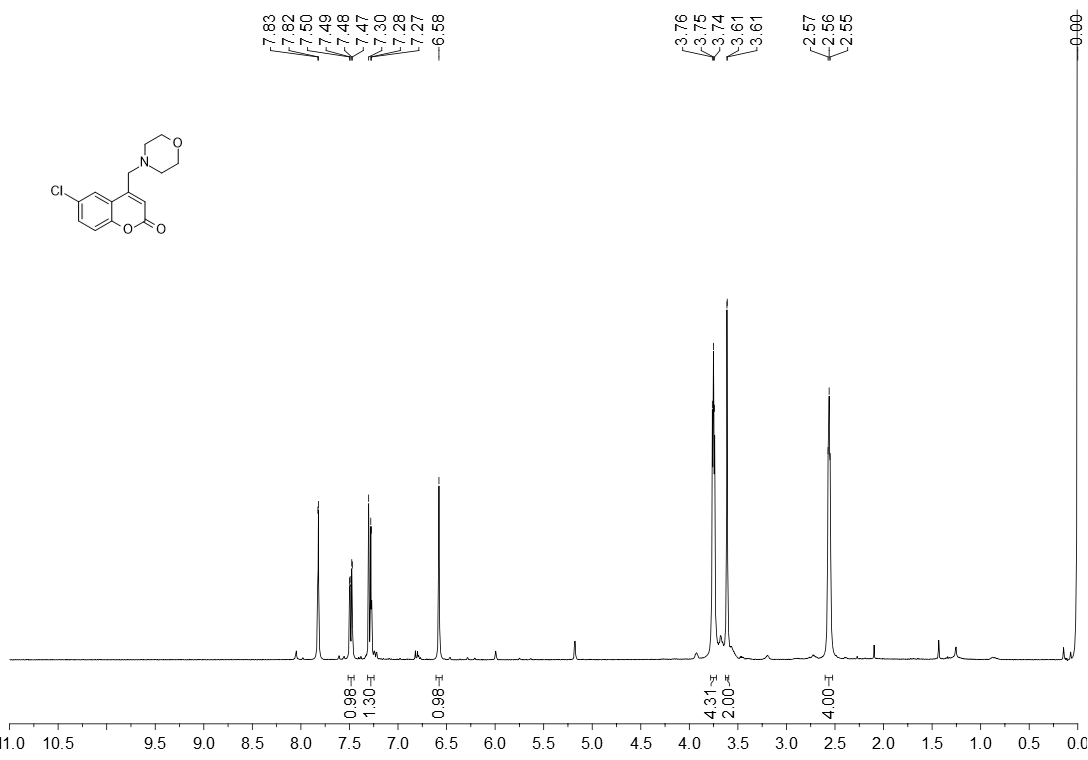


# **Figure S20**. ^1^H NMR spectrum (300 MHz, CDCl_3_) of compound **9k.**


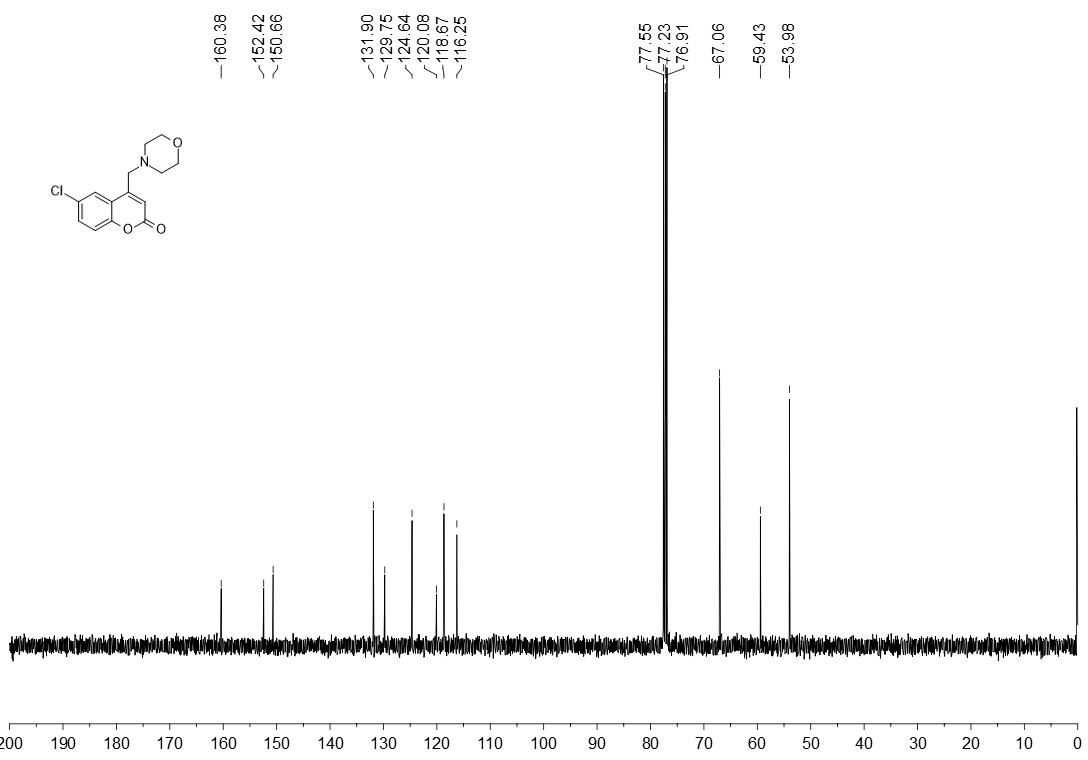


# **Figure S21**. ^13^C NMR spectrum (75 MHz, CDCl_3_) of compound **9k.**


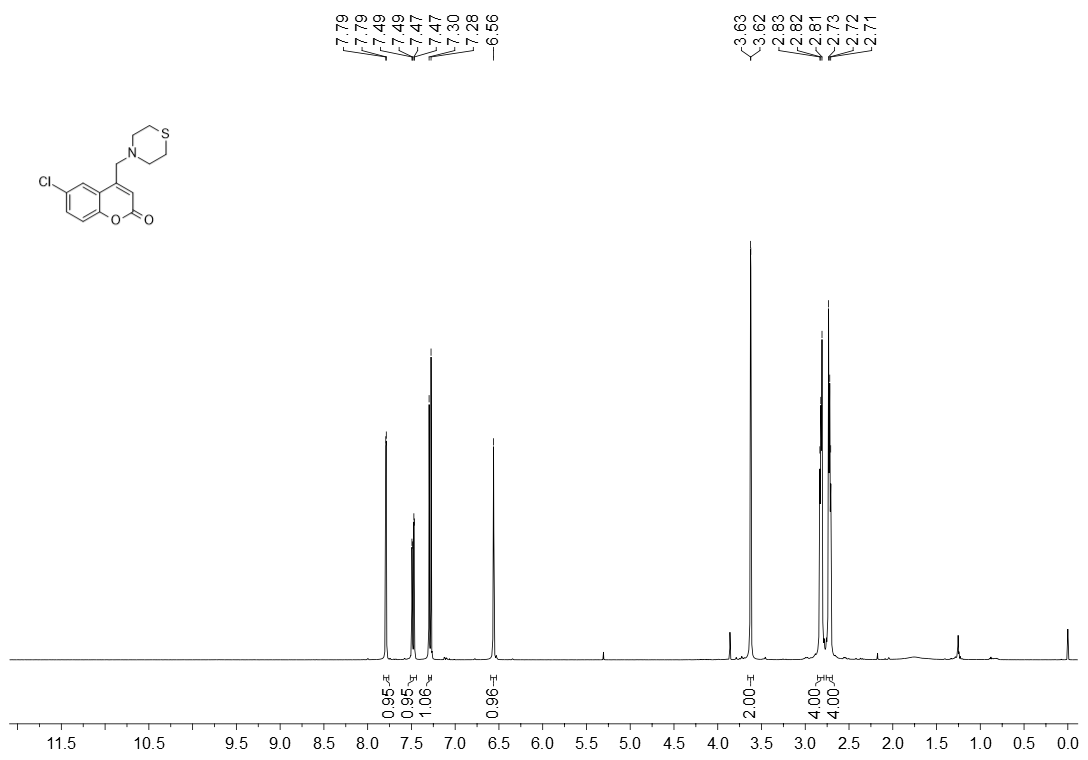


# **Figure S22**. ^1^H NMR spectrum (400 MHz, CDCl_3_) of compound **9l.**


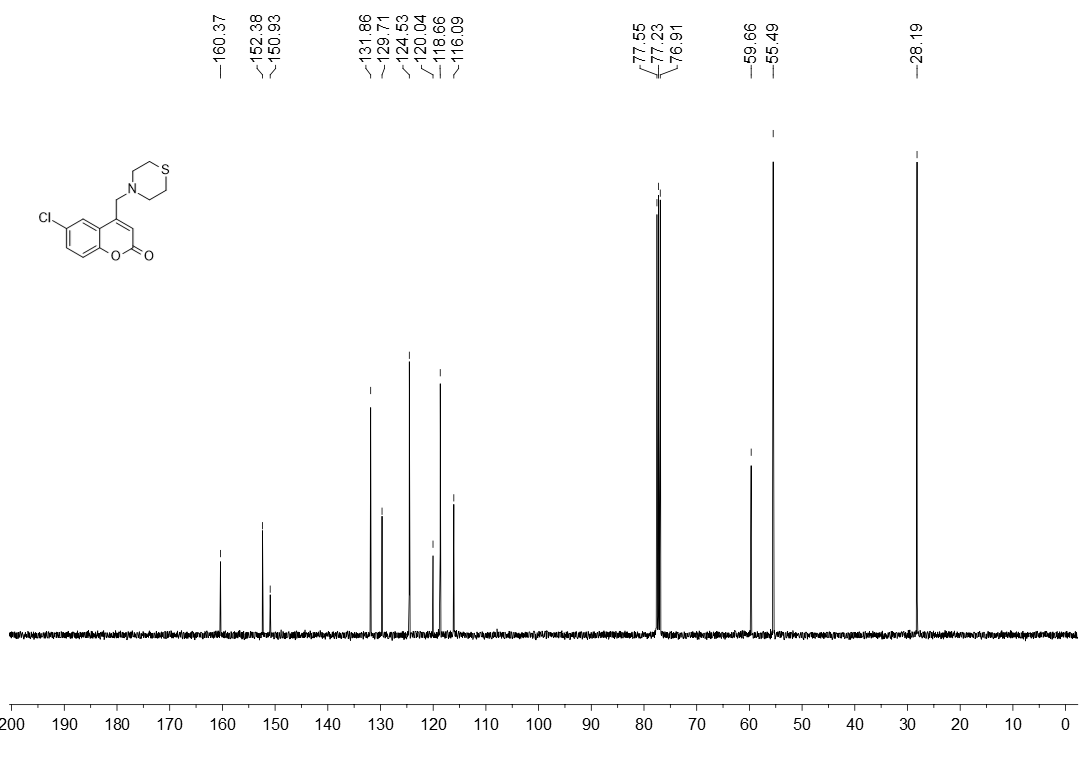


# **Figure S23**. ^13^C NMR spectrum (100 MHz, CDCl_3_) of compound **9l.**


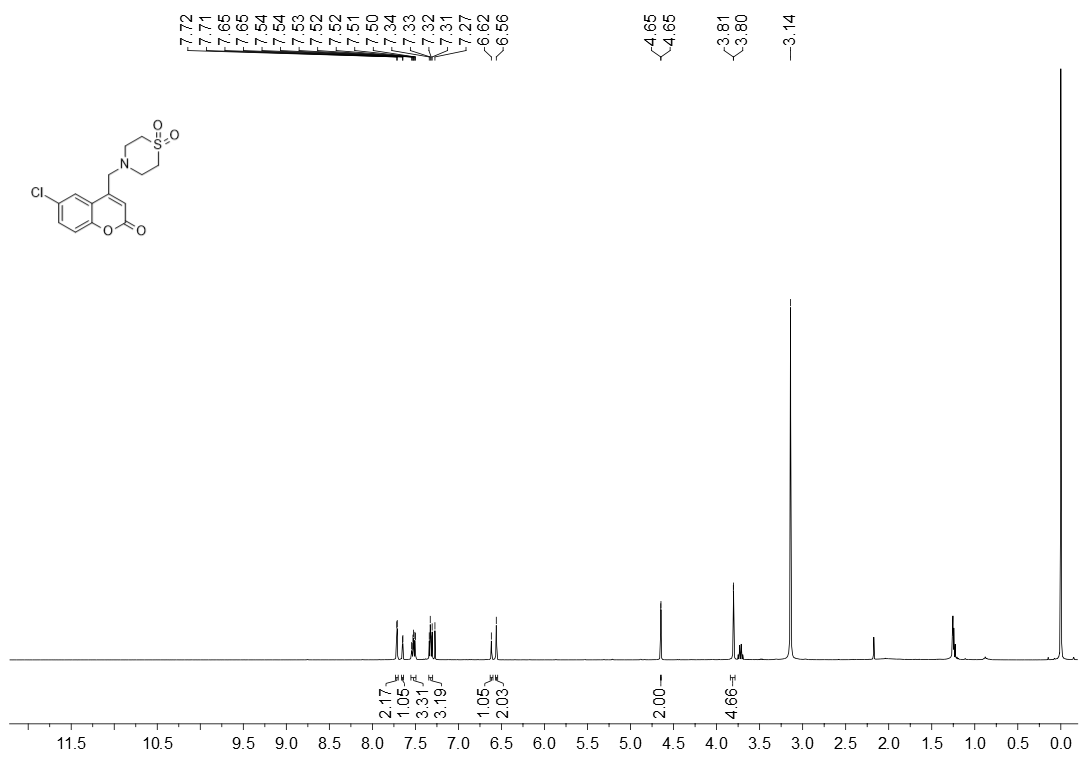


# **Figure S24**. ^1^H NMR spectrum (400 MHz, CDCl_3_) of compound **9m.**


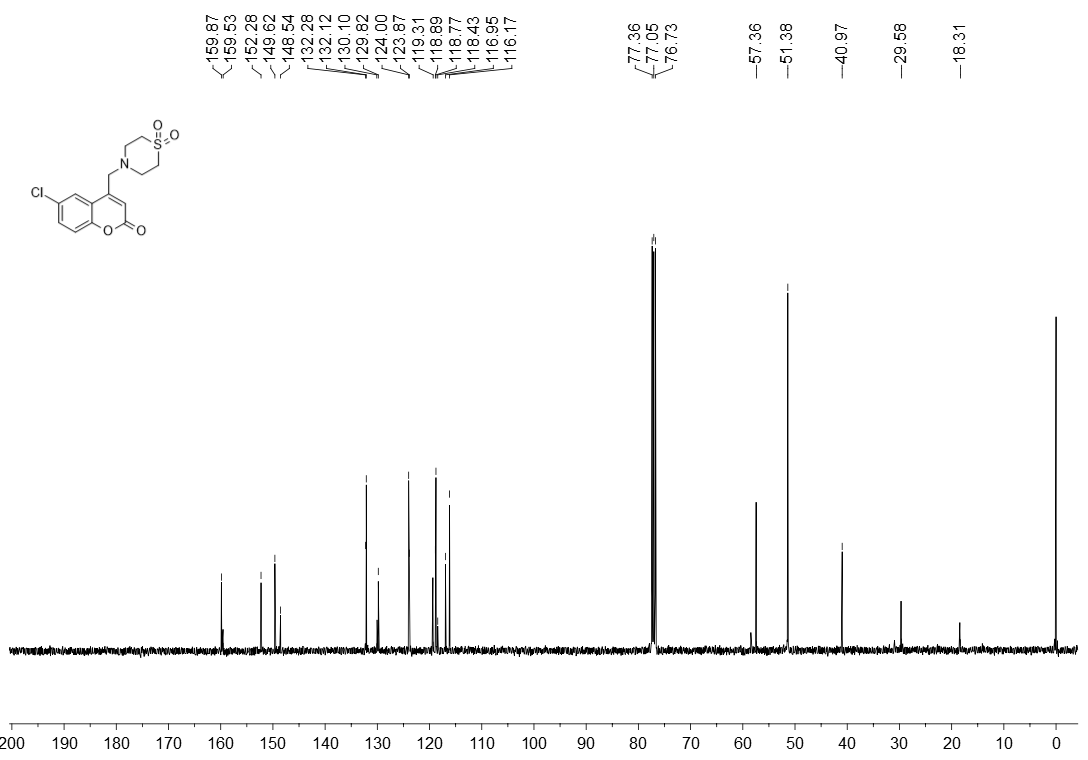


# **Figure S25**. ^13^C NMR spectrum (100 MHz, CDCl_3_) of compound **9m.**

#
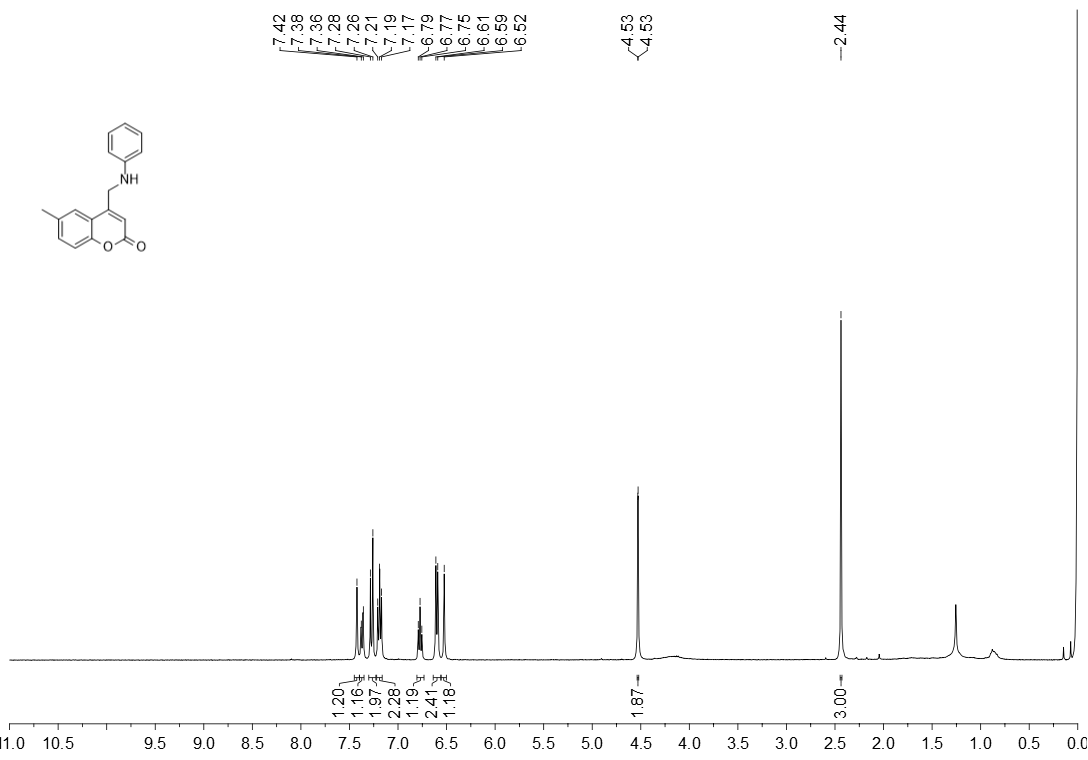


# **Figure S26**. ^1^H NMR spectrum (300 MHz, CDCl_3_) of compound **10a.**


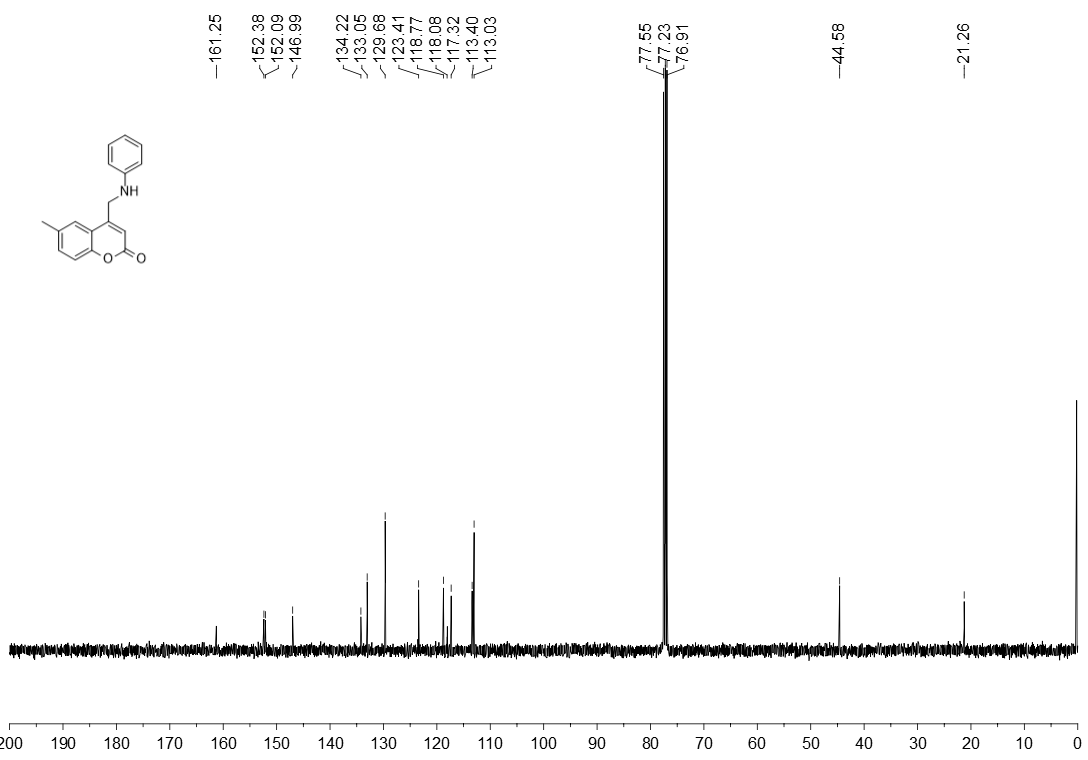


# **Figure S27**. ^13^C NMR spectrum (75 MHz, CDCl_3_) of compound **10a.**


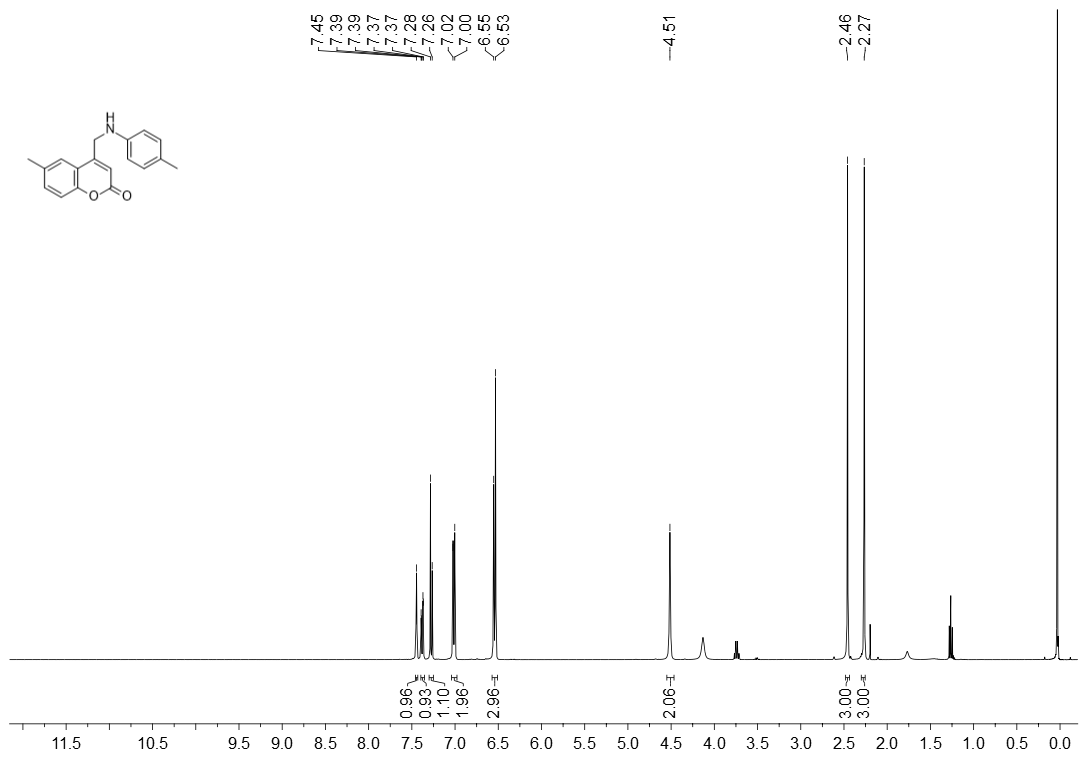


# **Figure S28**. ^1^H NMR spectrum (400 MHz, CDCl_3_) of compound **10b.**


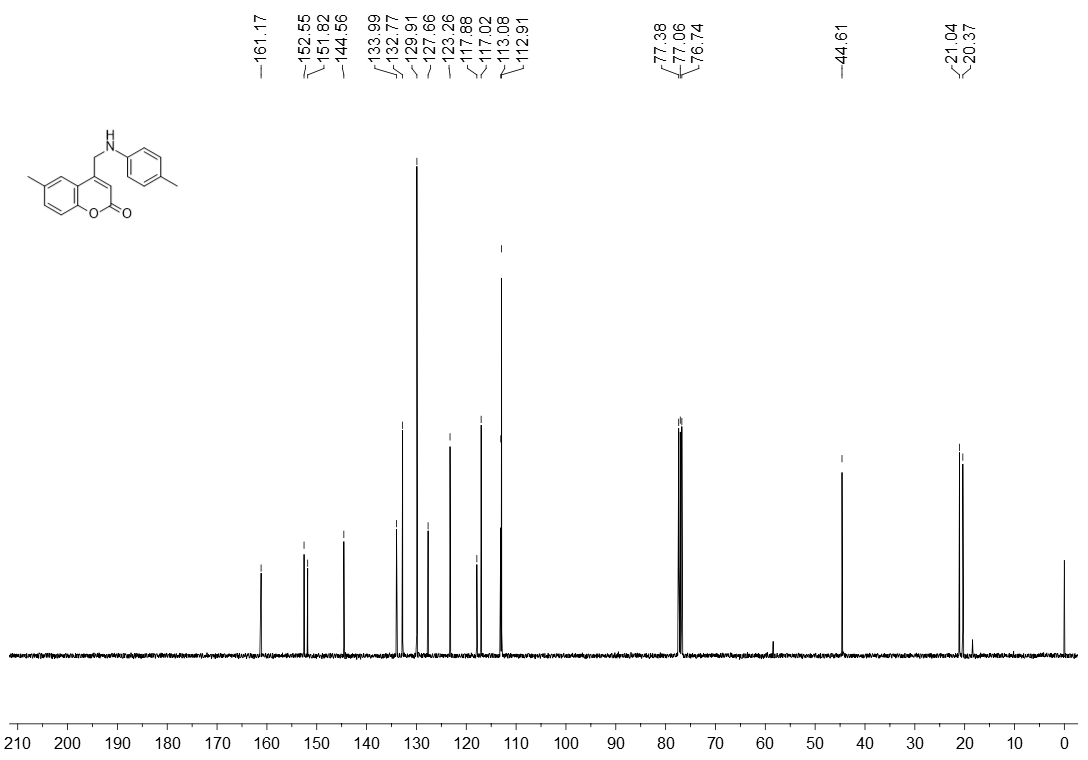


# **Figure S29**. ^13^C NMR spectrum (100 MHz, CDCl_3_) of compound **10b.**


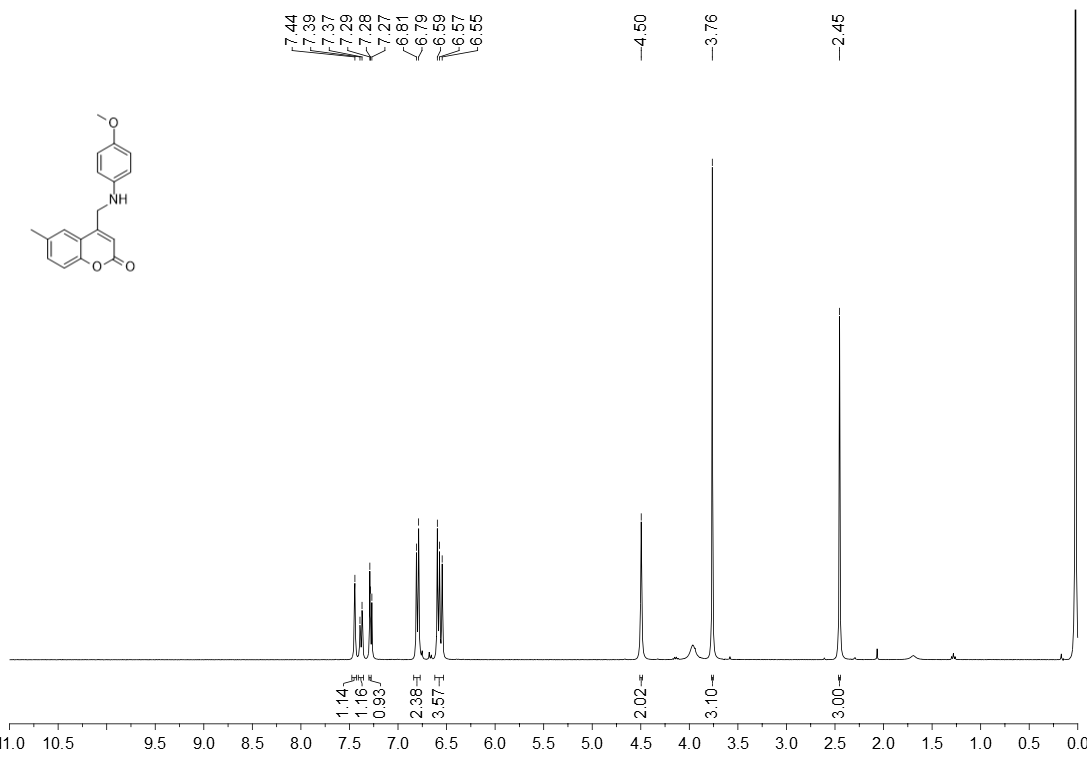


# **Figure S30**. ^1^H NMR spectrum (300 MHz, CDCl_3_) of compound **10e**


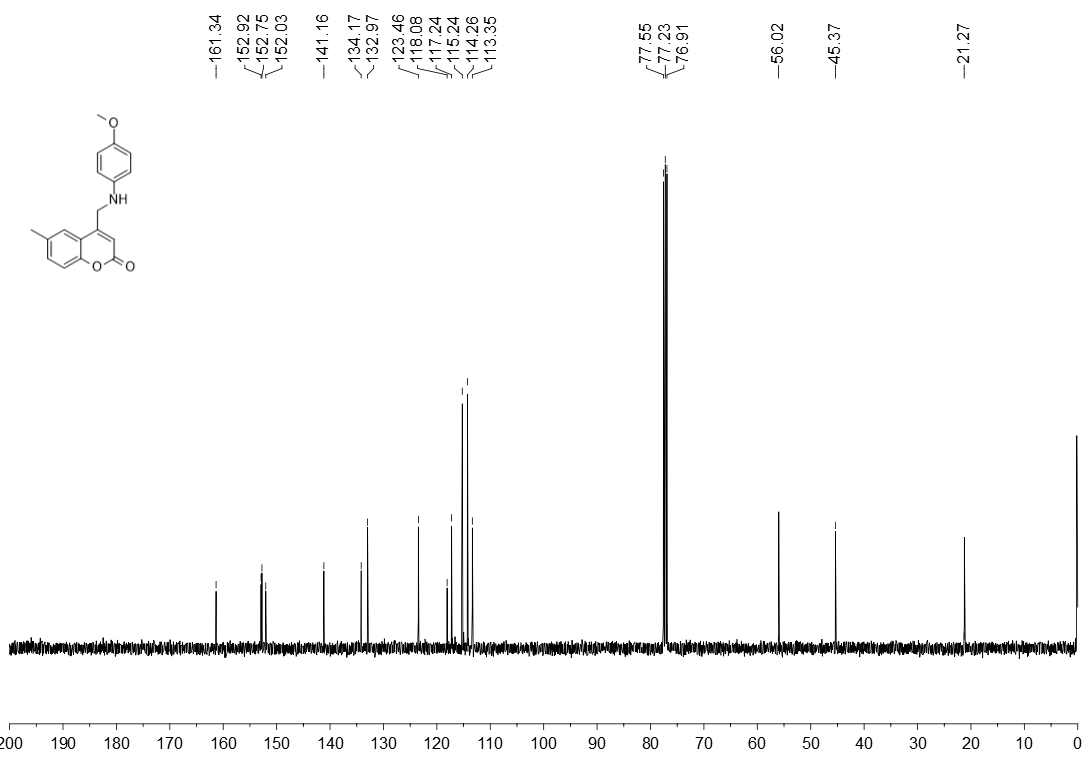


# **Figure S31**. ^13^C NMR spectrum (75 MHz, CDCl_3_) of compound **10e.**


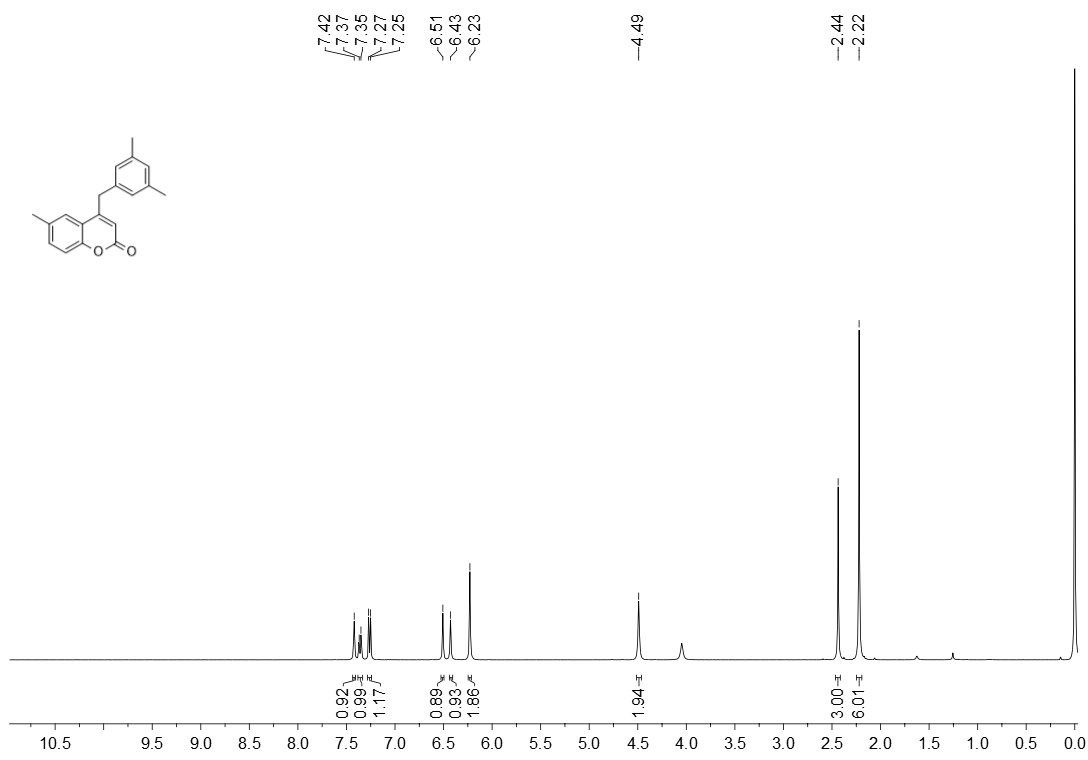


# **Figure S32**. ^1^H NMR spectrum (300 MHz, CDCl_3_) of compound **10f**


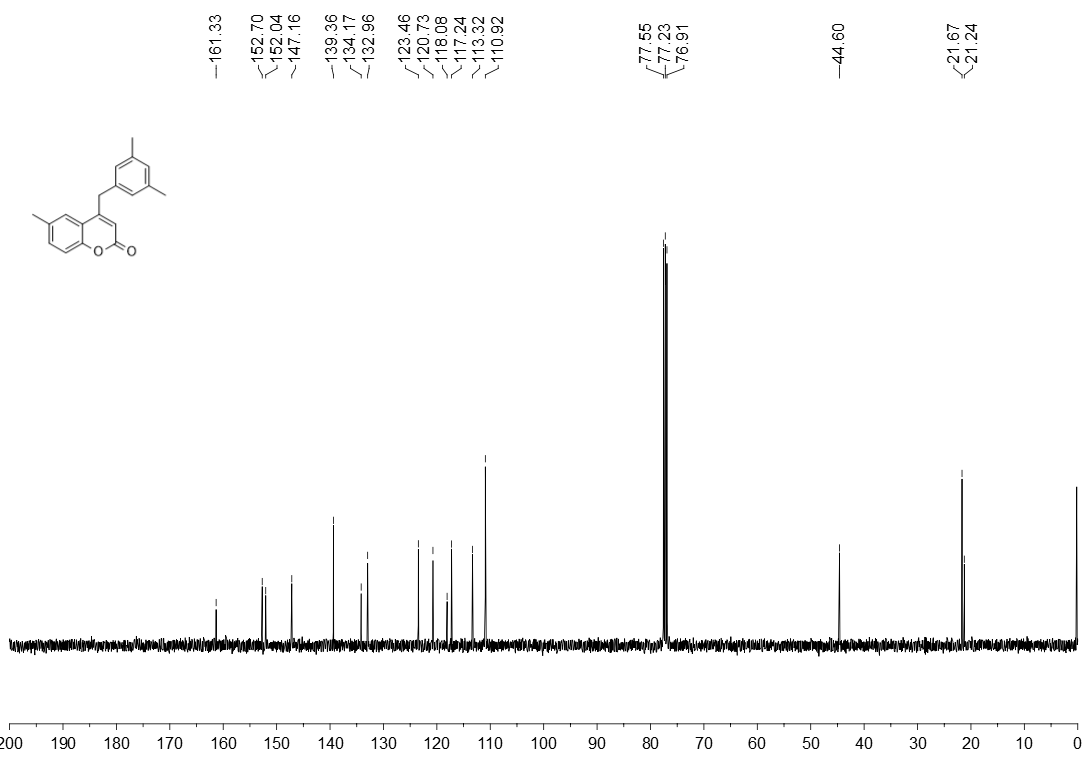


# **Figure S33**. ^13^C NMR spectrum (75 MHz, CDCl_3_) of compound **10f.**

#
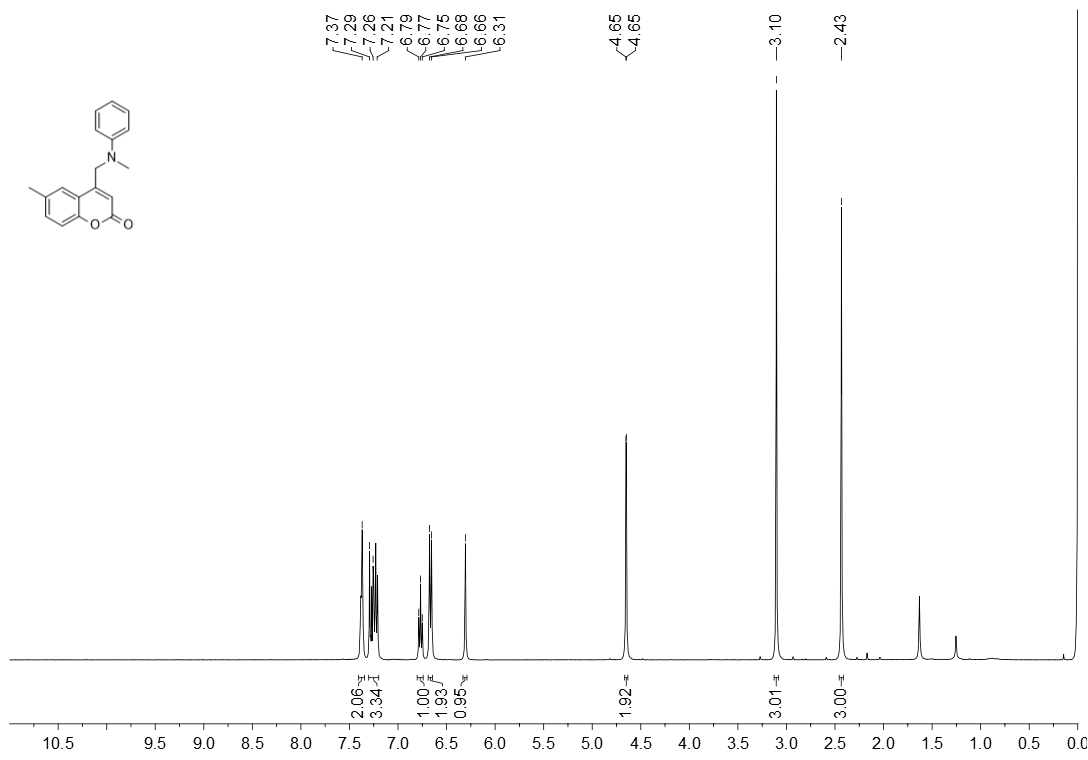


# **Figure S34**. ^1^H NMR spectrum (300 MHz, CDCl_3_) of compound **10g**


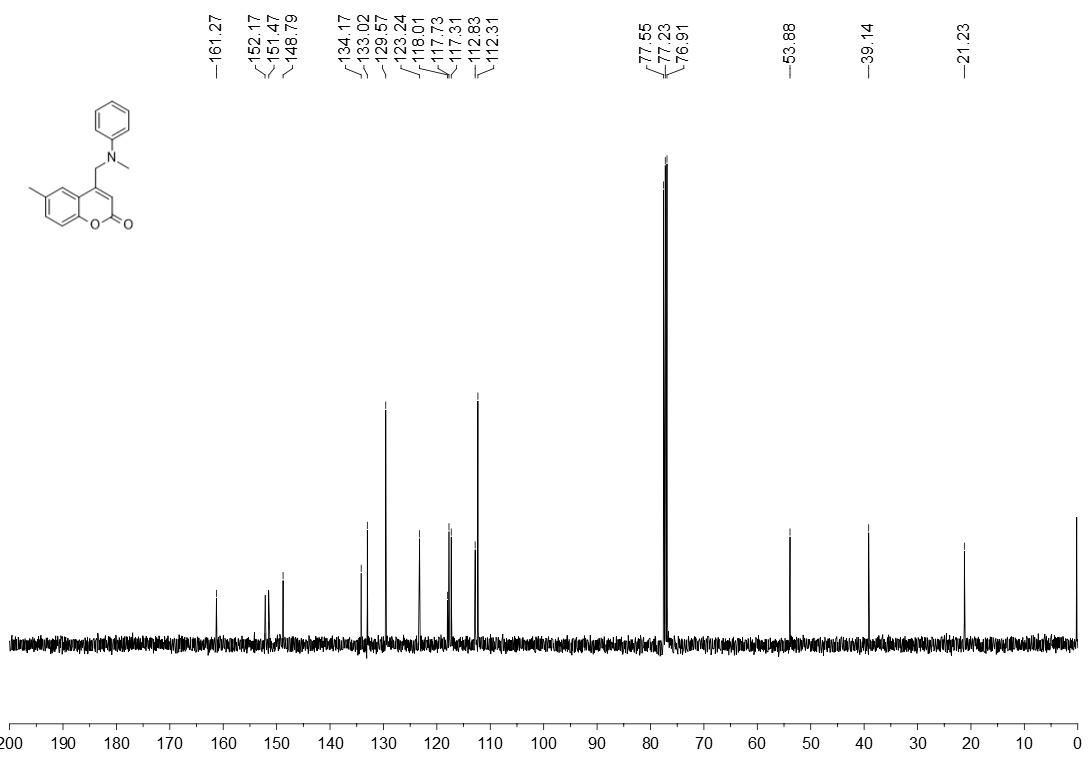


# **Figure S35**. ^13^C NMR spectrum (75 MHz, CDCl_3_) of compound **10g.**

#
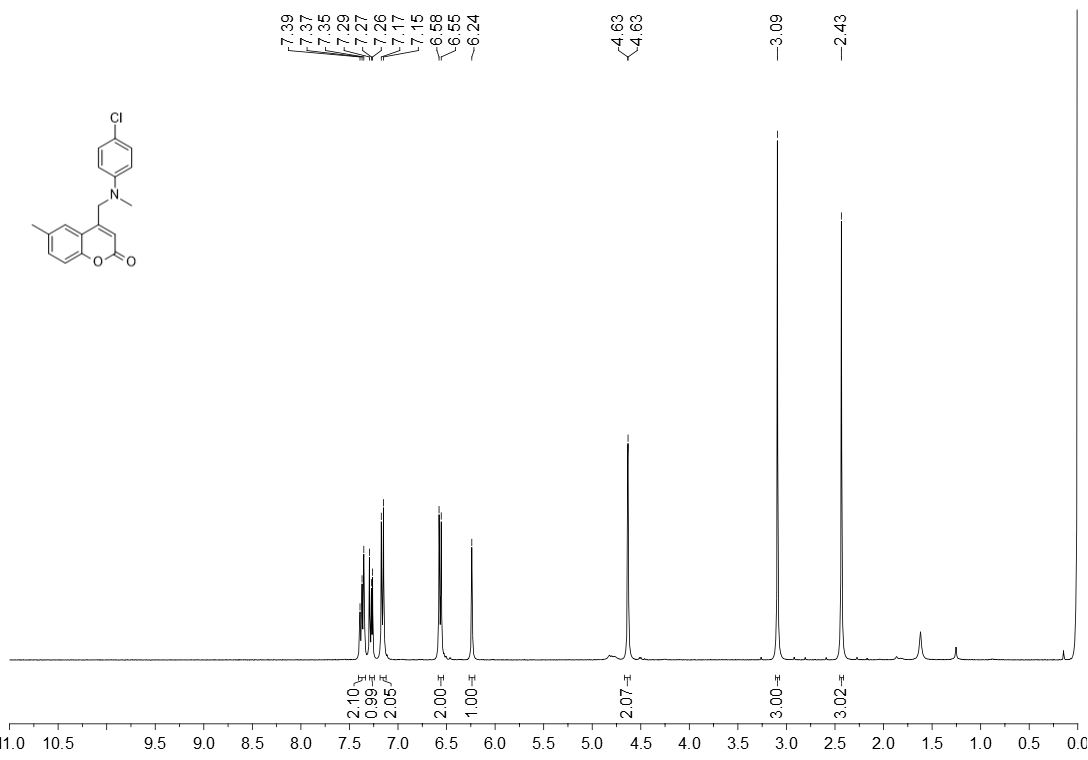


# **Figure S36**. ^1^H NMR spectrum (300 MHz, CDCl_3_) of compound **10h**


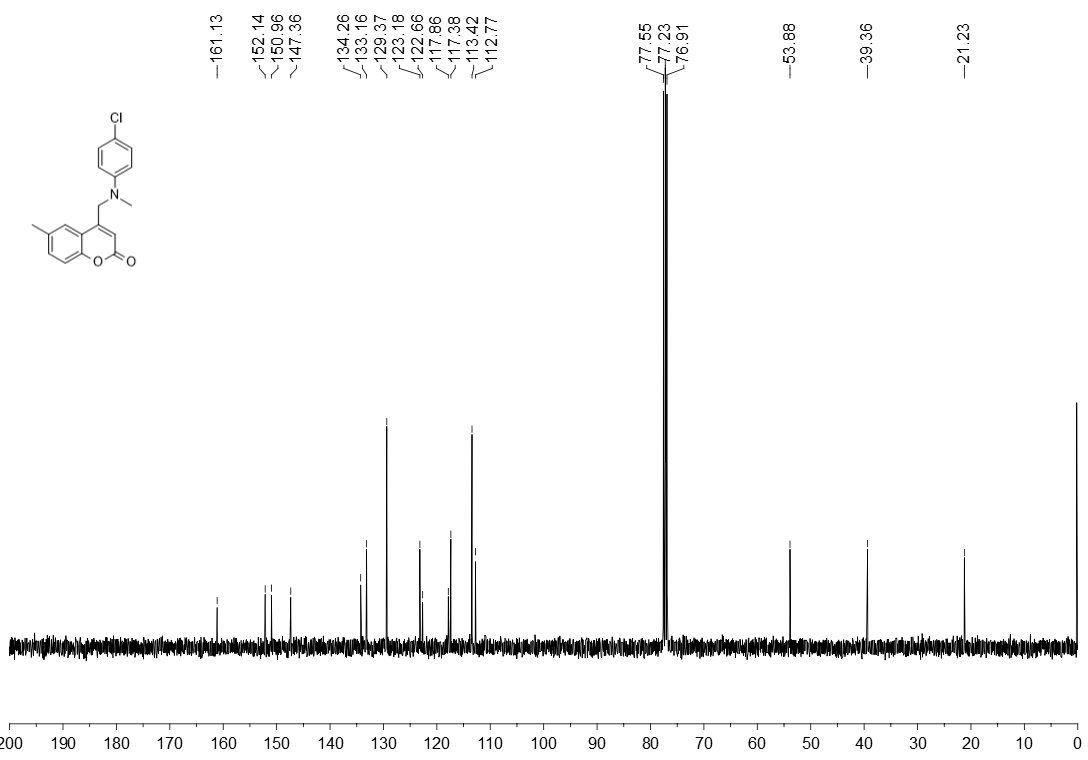


# **Figure S37**. ^13^C NMR spectrum (75 MHz, CDCl_3_) of compound **10h.**

#
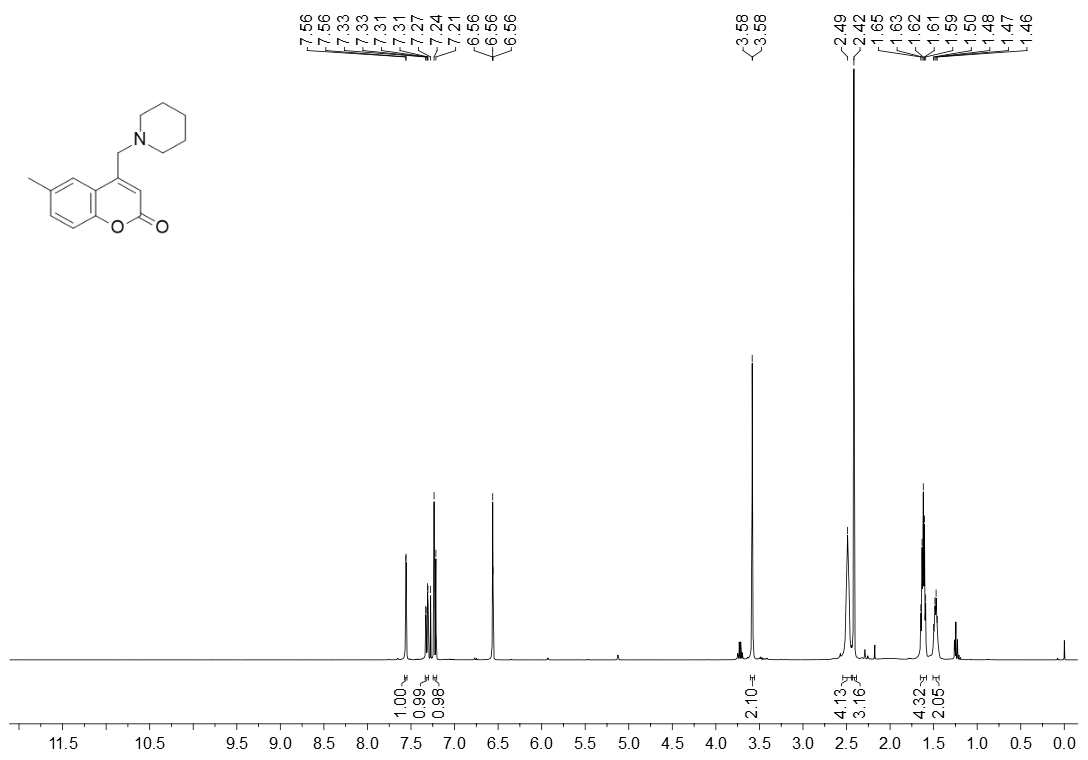


# **Figure S38**. ^1^H NMR spectrum (400 MHz, CDCl_3_) of compound **10i**


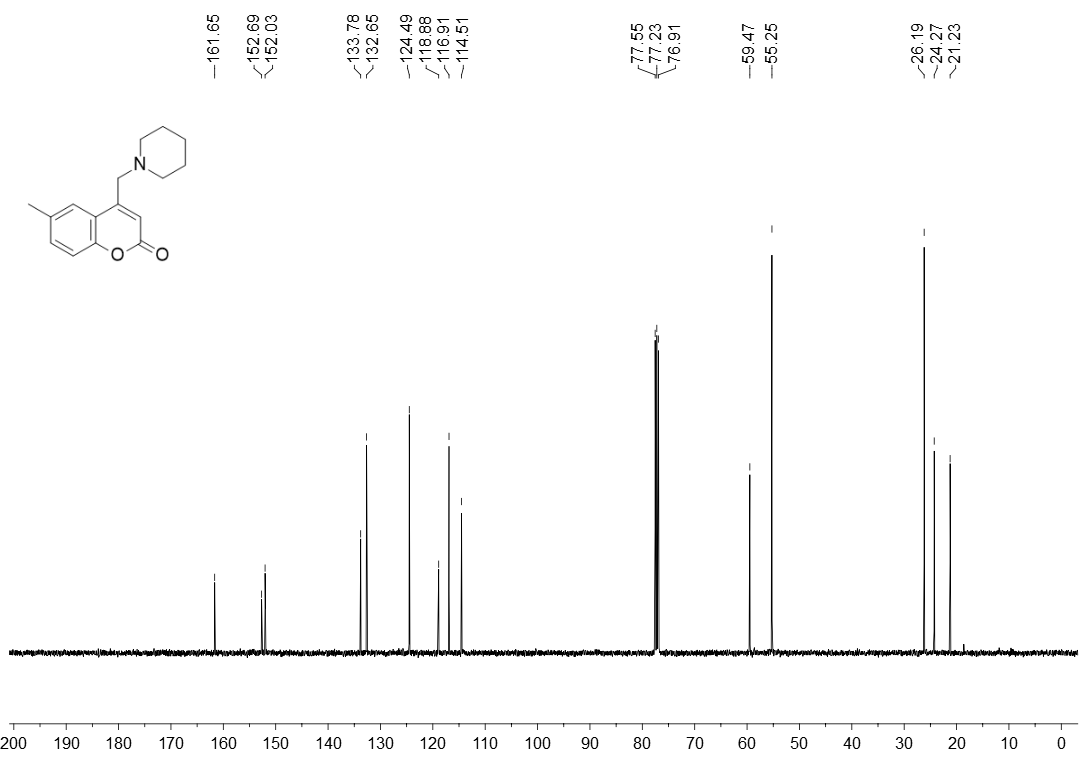


# **Figure S39**. ^13^C NMR spectrum (100 MHz, CDCl_3_) of compound **10i.**


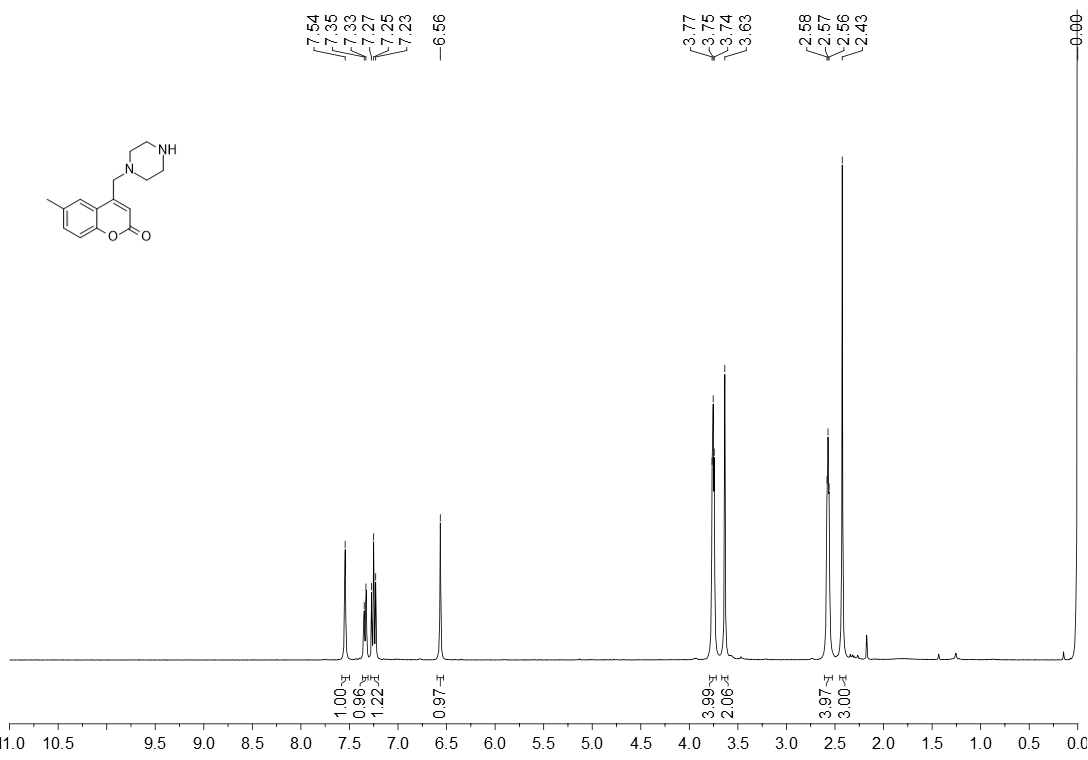


# **Figure S40**. ^1^H NMR spectrum (300 MHz, CDCl_3_) of compound **10j**


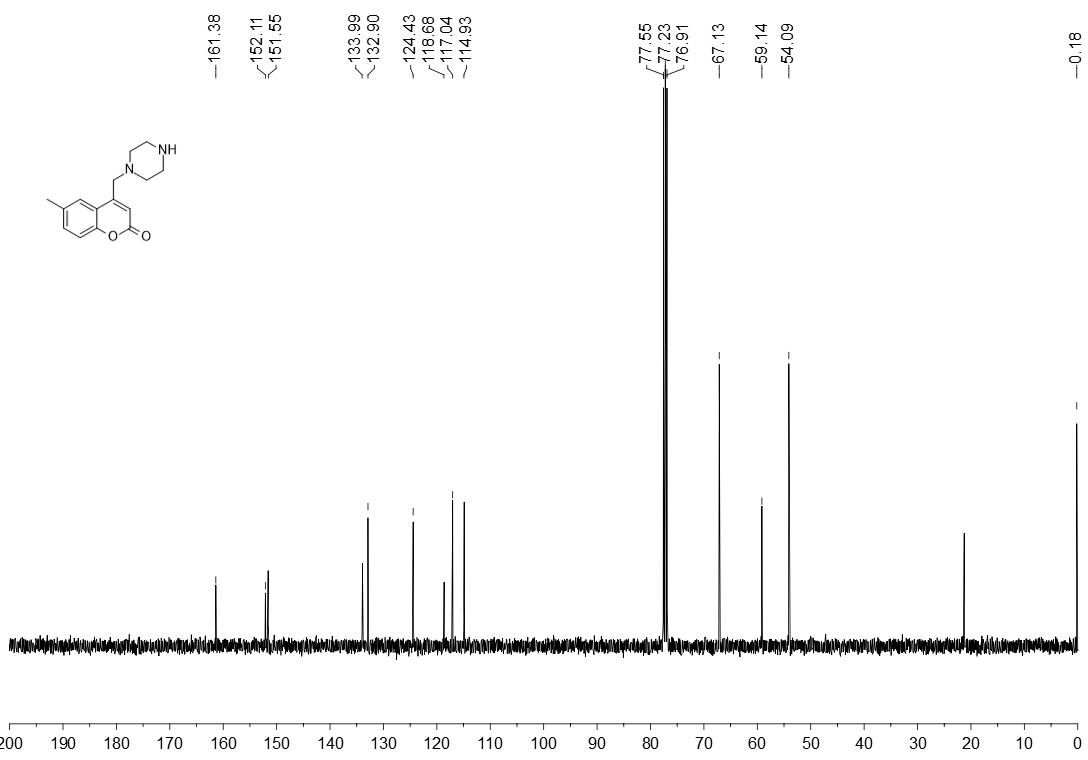


# **Figure S41**. ^13^C NMR spectrum (75 MHz, CDCl_3_) of compound **10j.**


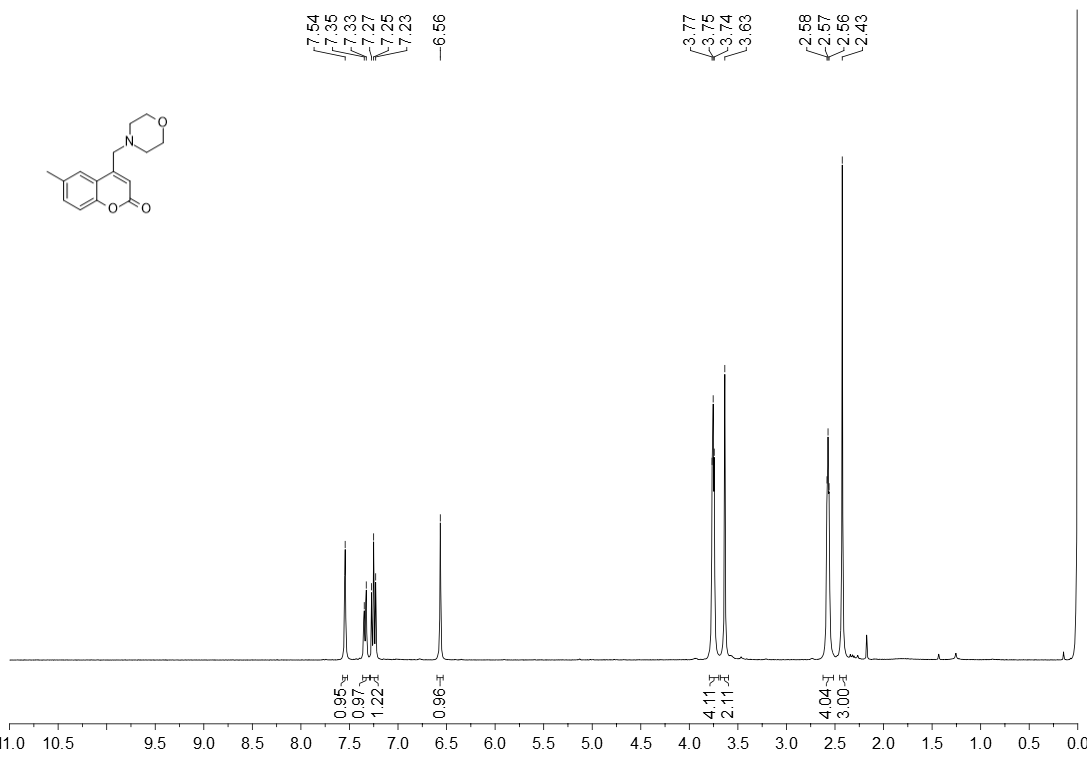


# **Figure S42**. ^1^H NMR spectrum (300 MHz, CDCl_3_) of compound **10k**


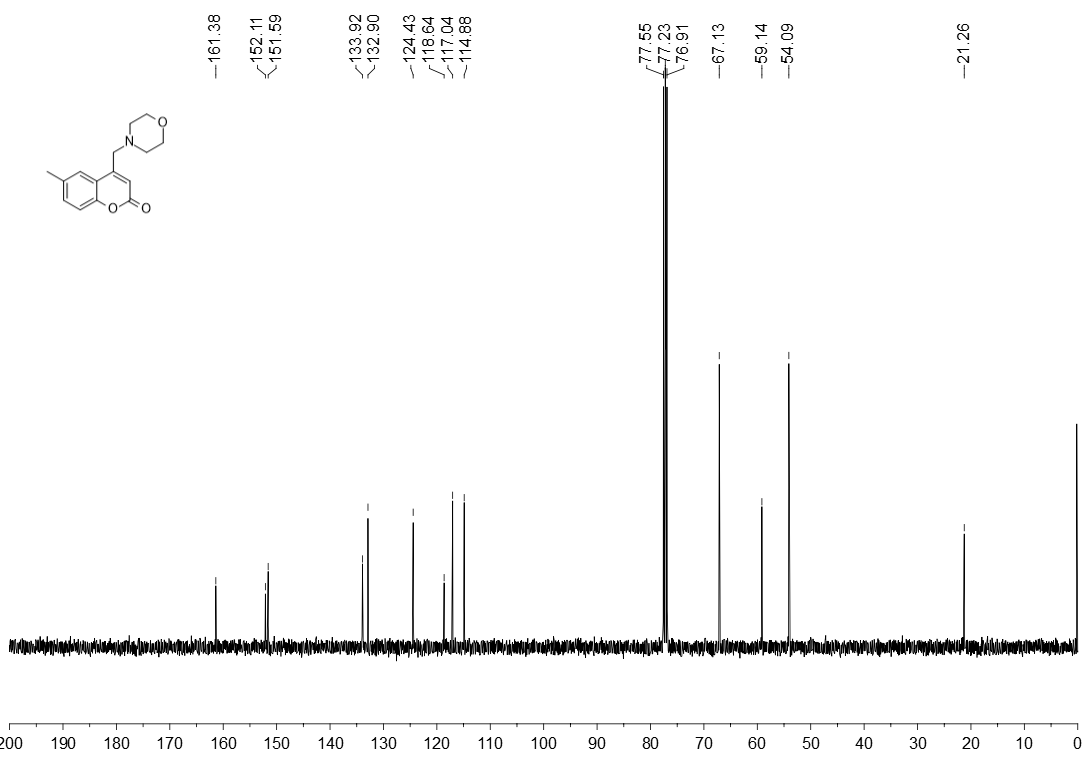


# **Figure S43**. ^13^C NMR spectrum (75 MHz, CDCl_3_) of compound **10k.**


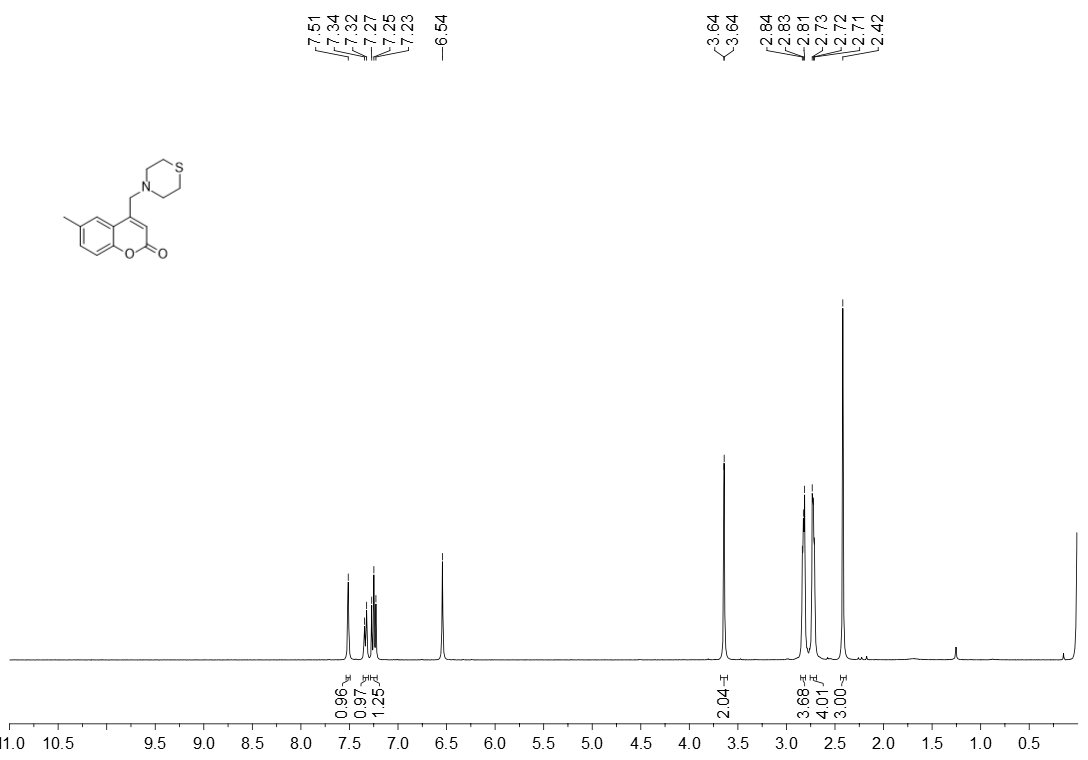


# **Figure S44**. ^1^H NMR spectrum (300 MHz, CDCl_3_) of compound **10l**


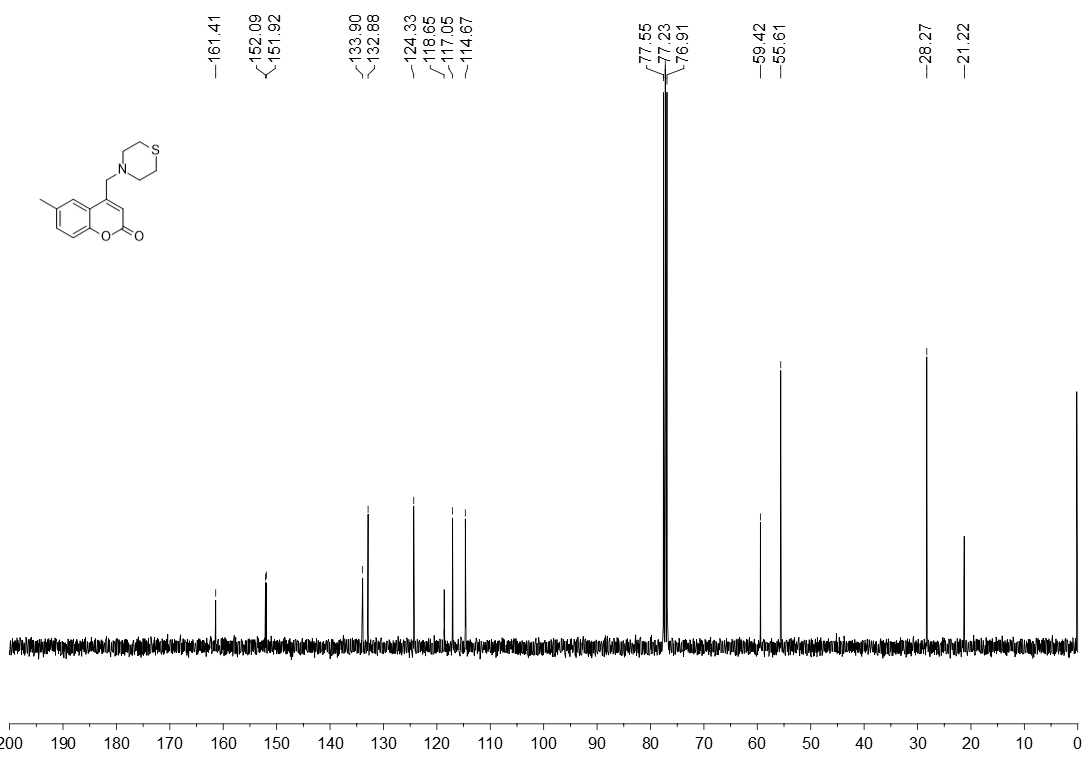


# **Figure S45**. ^13^C NMR spectrum (75 MHz, CDCl_3_) of compound **10l.**


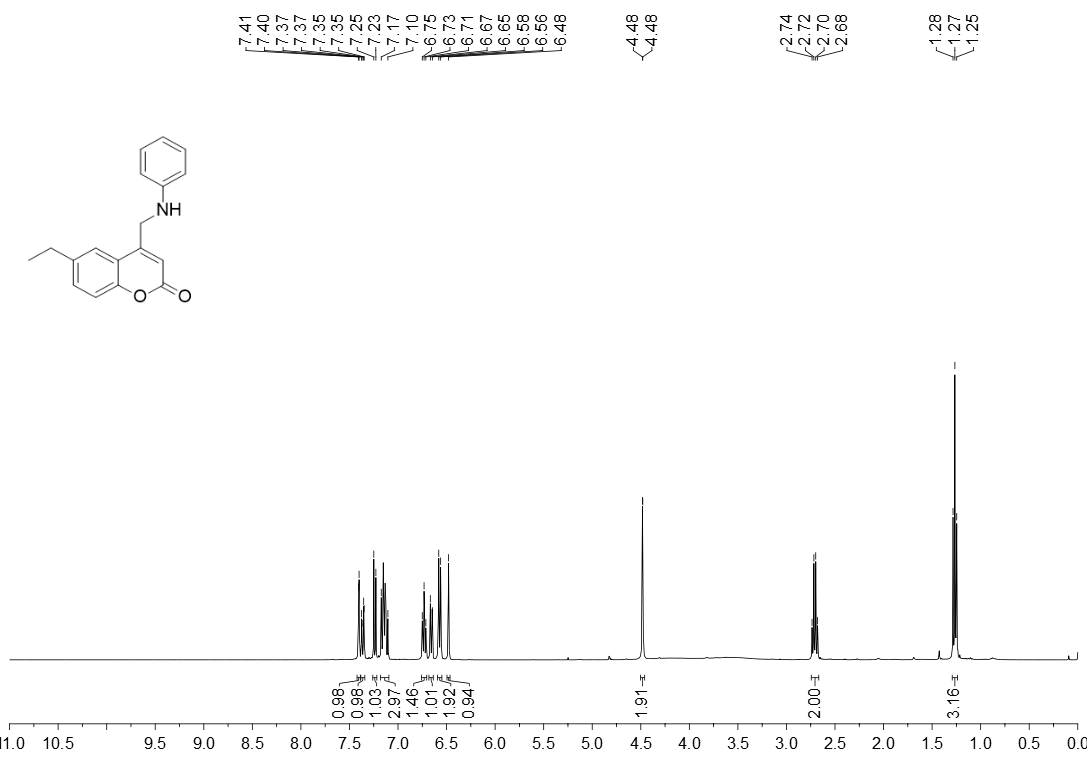


**Figure S46**. ^1^H NMR spectrum (300 MHz, CDCl_3_) of compound **11a**


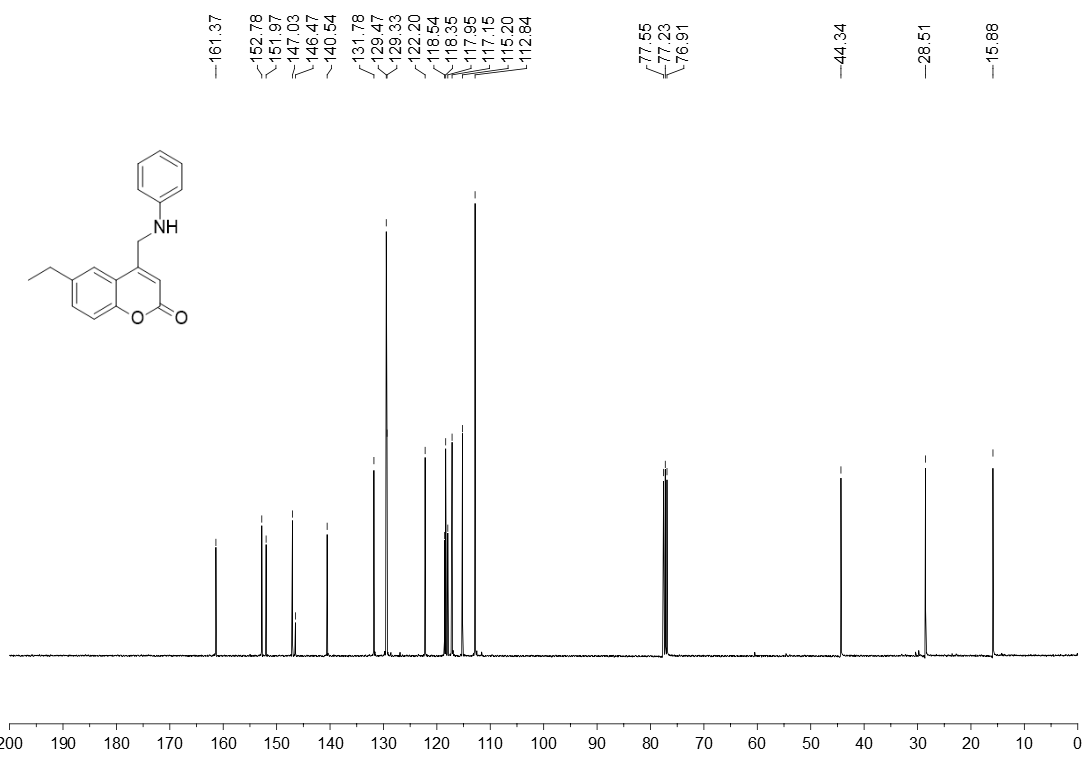


# **Figure S47**. ^13^C NMR spectrum (75 MHz, CDCl_3_) of compound **11a.**


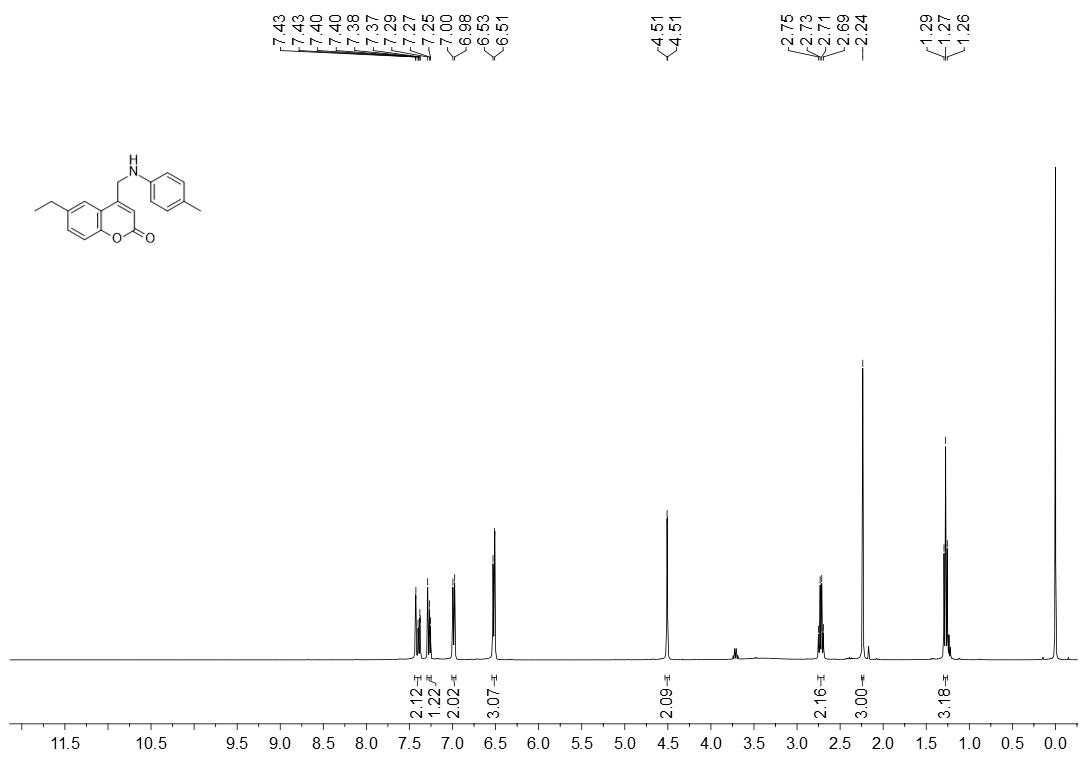


**Figure S48.** ^1^H NMR spectrum (400 MHz, CDCl_3_) of compound **11b**


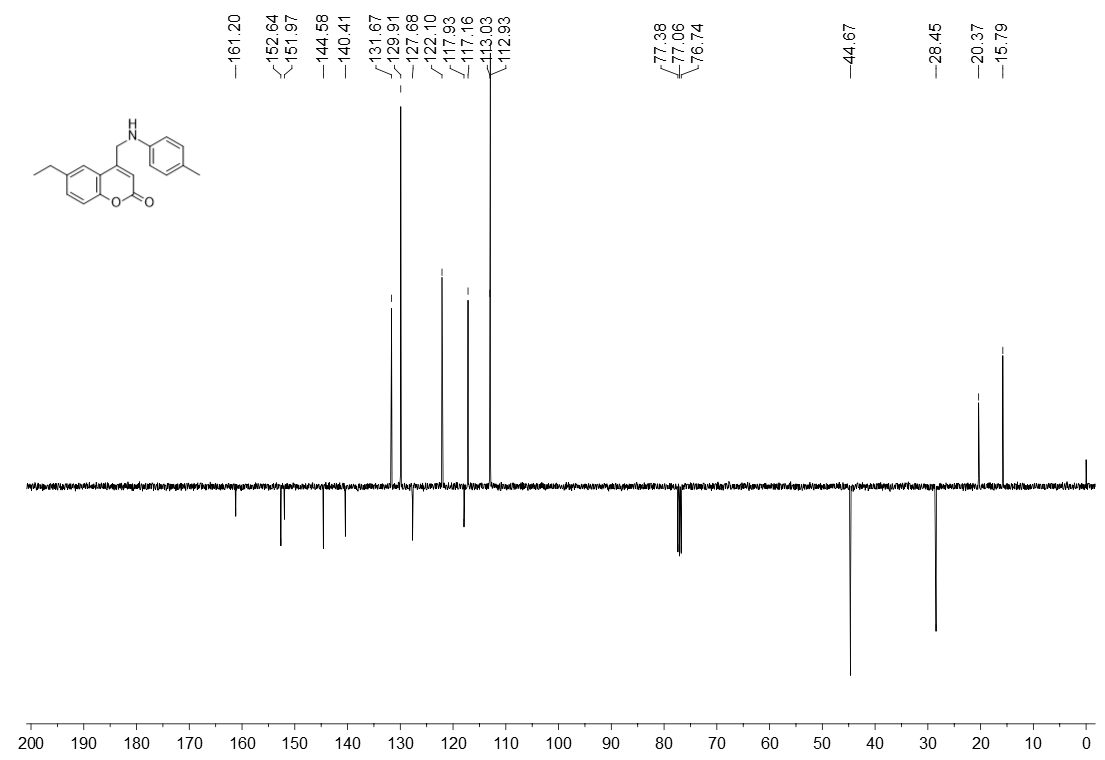


# **Figure S49**. ^13^C APT NMR spectrum (100 MHz, CDCl_3_) of compound **11b.**


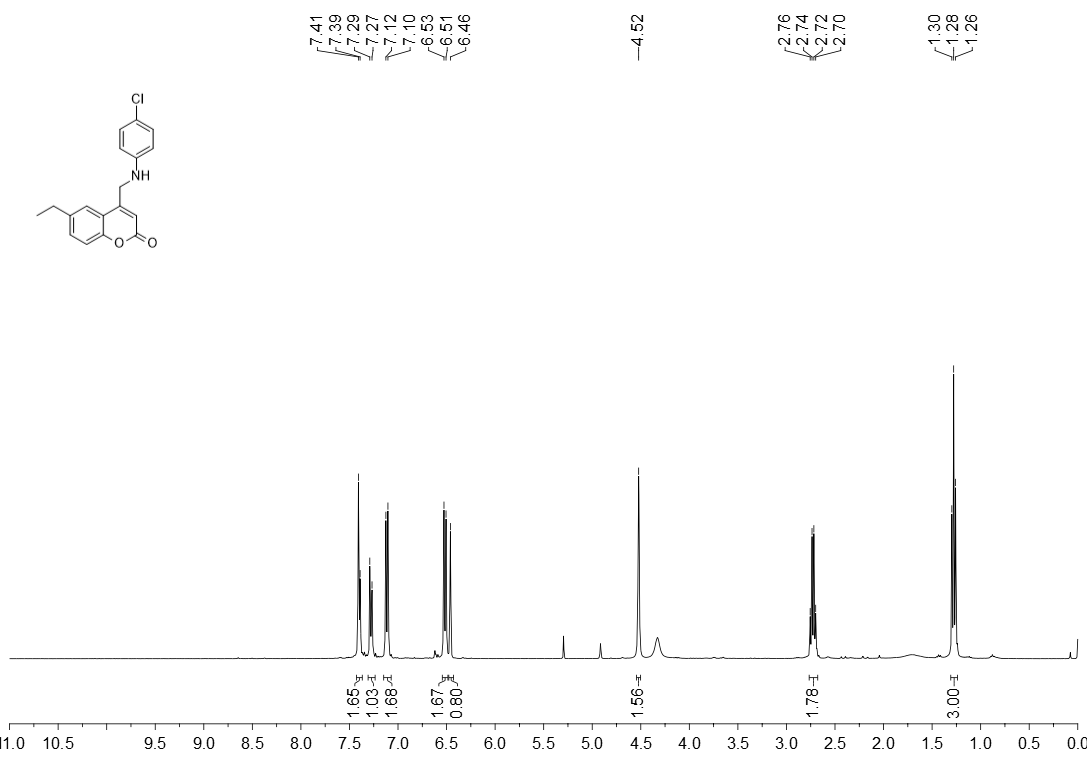


**Figure S50.** ^1^H NMR spectrum (300 MHz, CDCl_3_) of compound **11c**


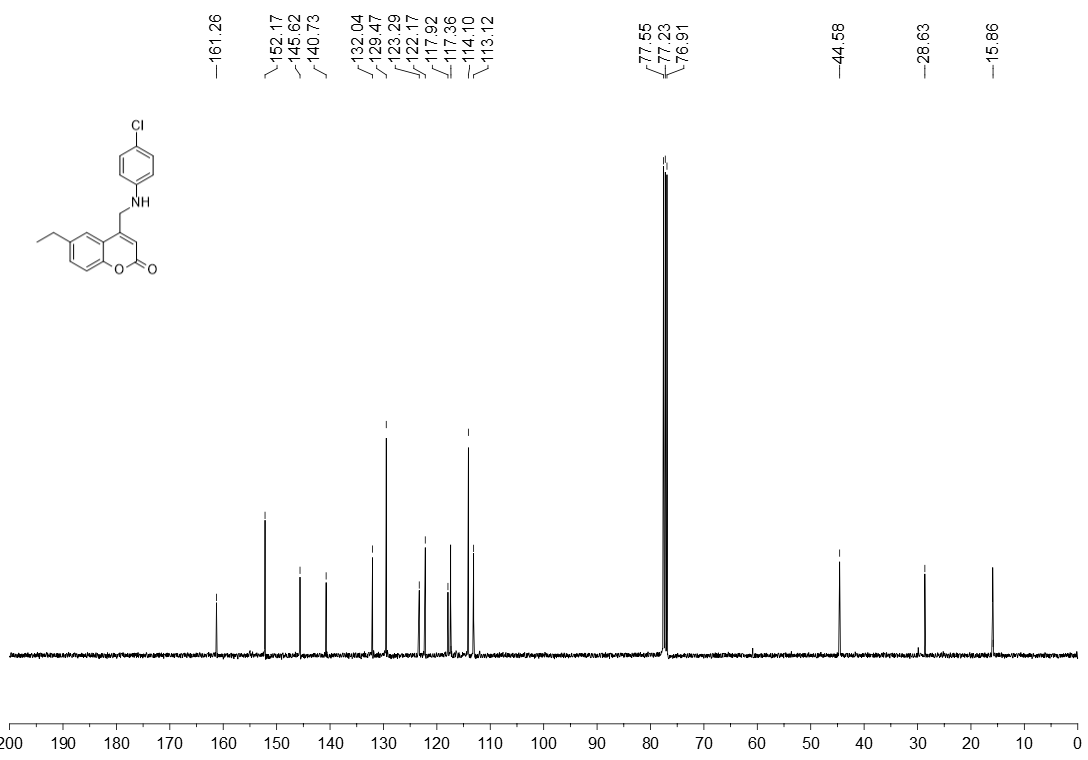


# **Figure S51**. ^13^C NMR spectrum (75 MHz, CDCl_3_) of compound **11c.**


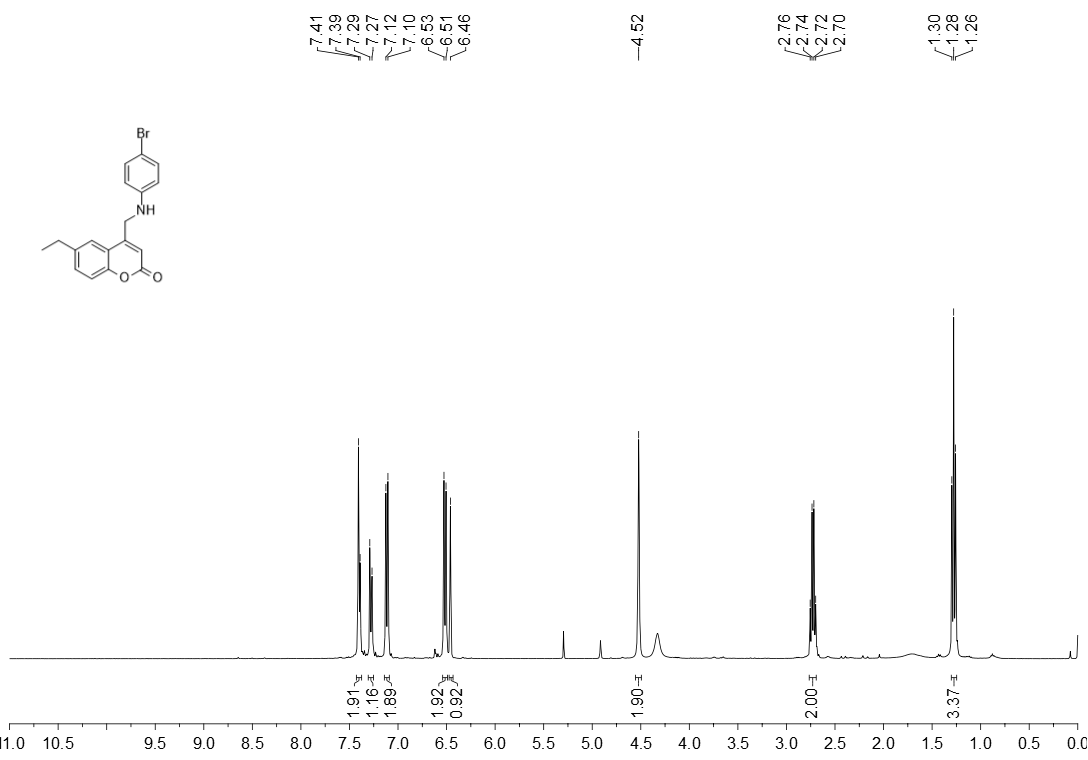


**Figure S52.** ^1^H NMR spectrum (300 MHz, CDCl_3_) of compound **11d**


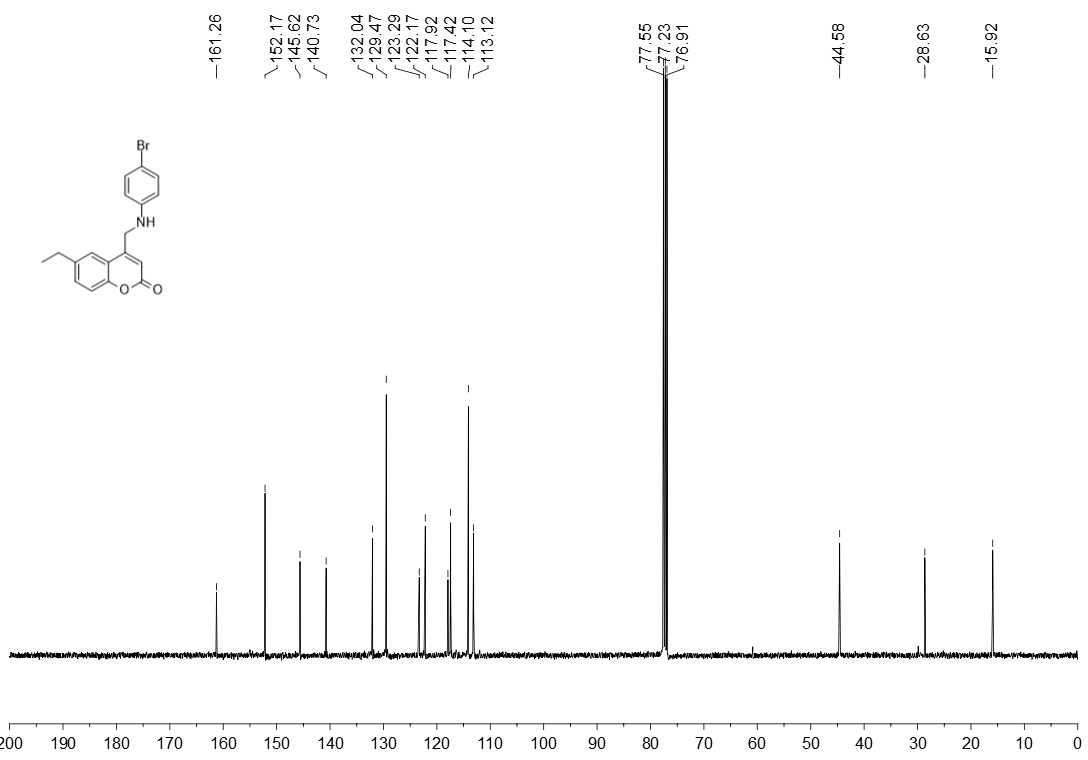


# **Figure S53**. ^13^C NMR spectrum (75 MHz, CDCl_3_) of compound **11d.**


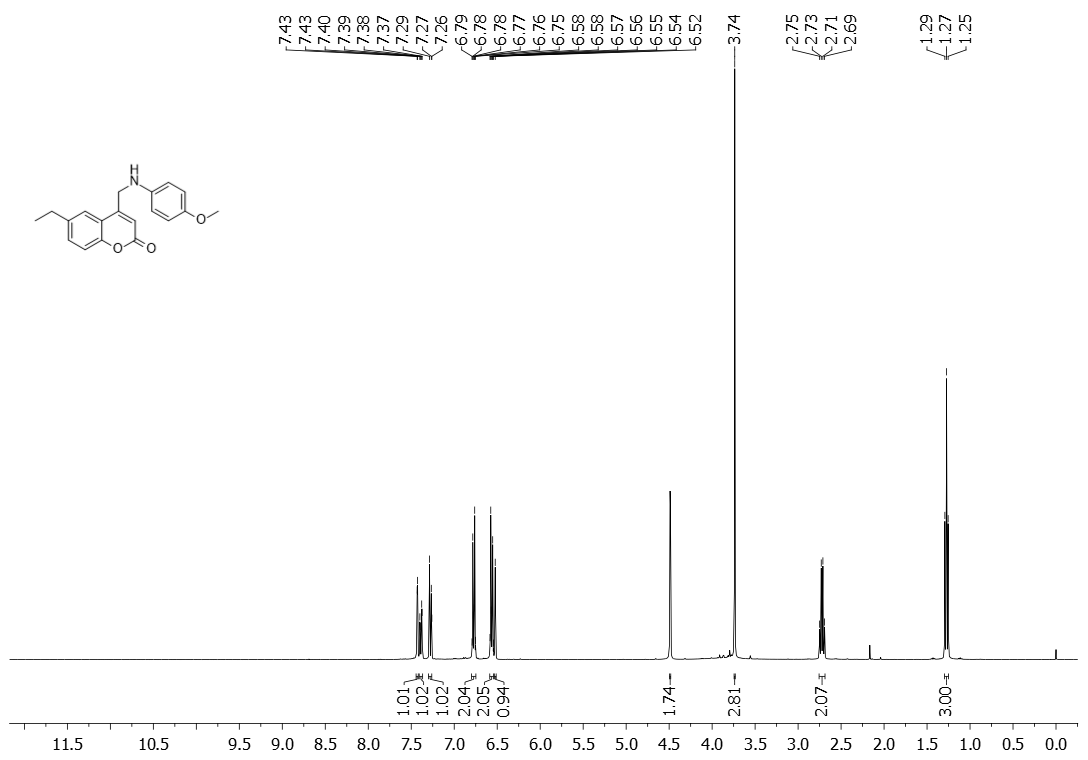


**Figure S54.** ^1^H NMR spectrum (400 MHz, CDCl_3_) of compound **11e**


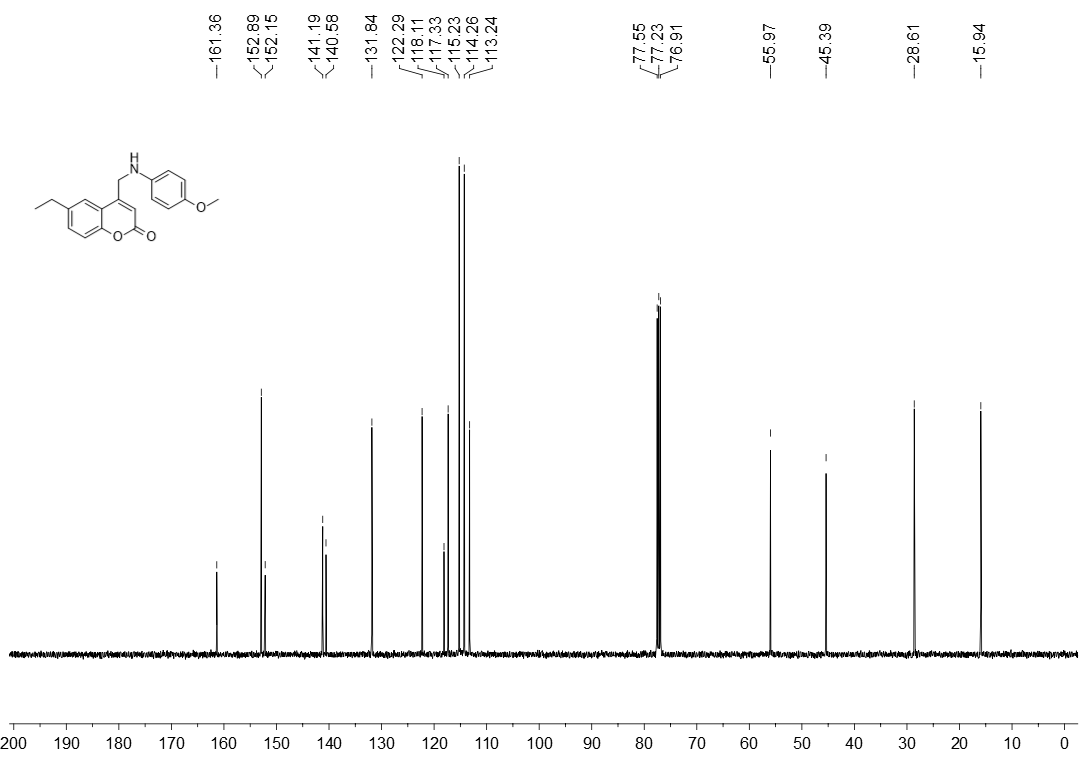


# **Figure S55**. ^13^C NMR spectrum (100 MHz, CDCl_3_) of compound **11e.**


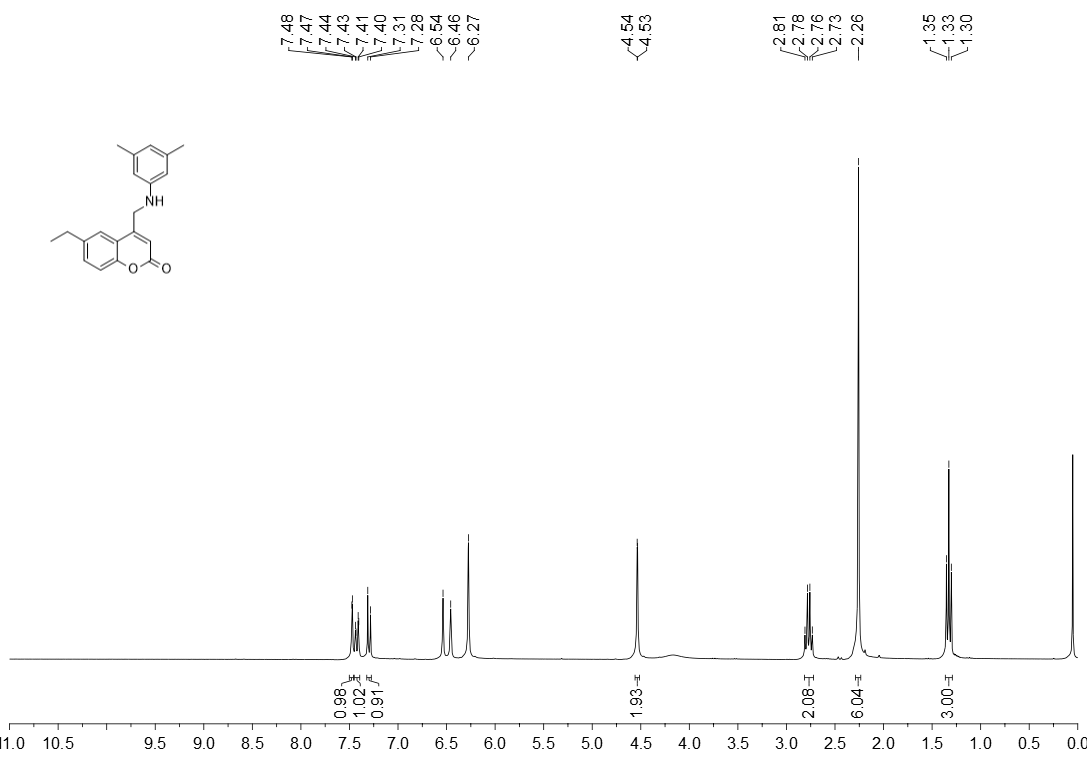


**Figure S56.** ^1^H NMR spectrum (300 MHz, CDCl_3_) of compound **11f**


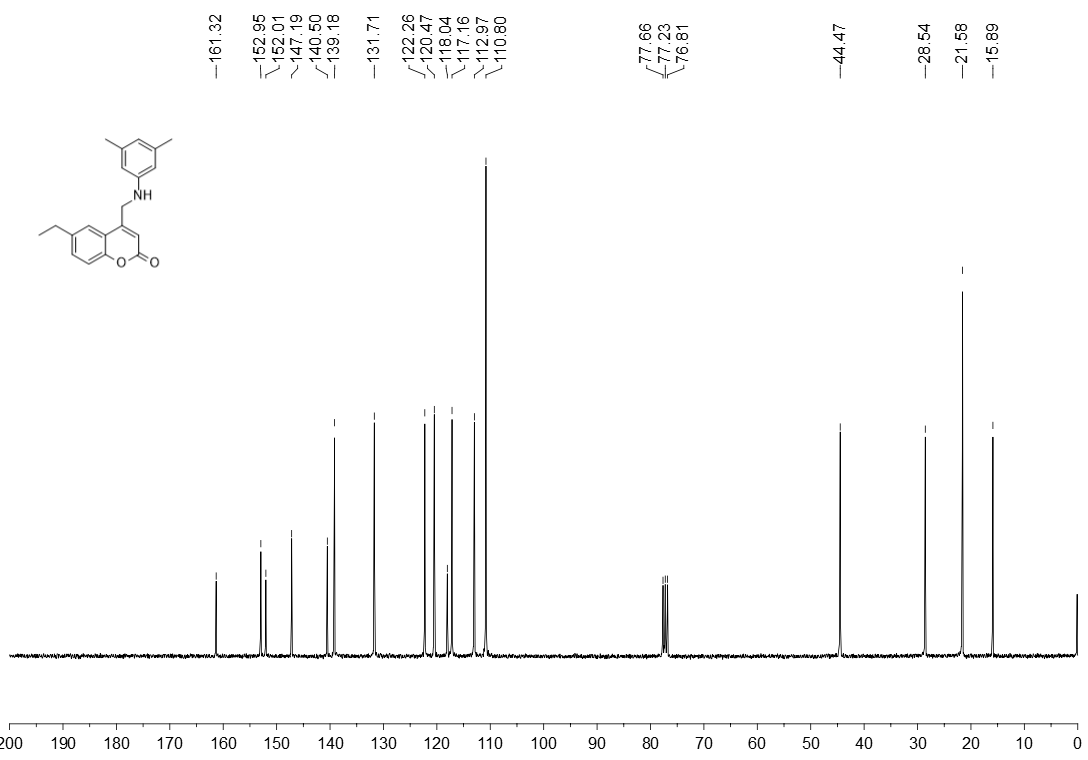


# **Figure S57**. ^13^C NMR spectrum (75 MHz, CDCl_3_) of compound **11f.**


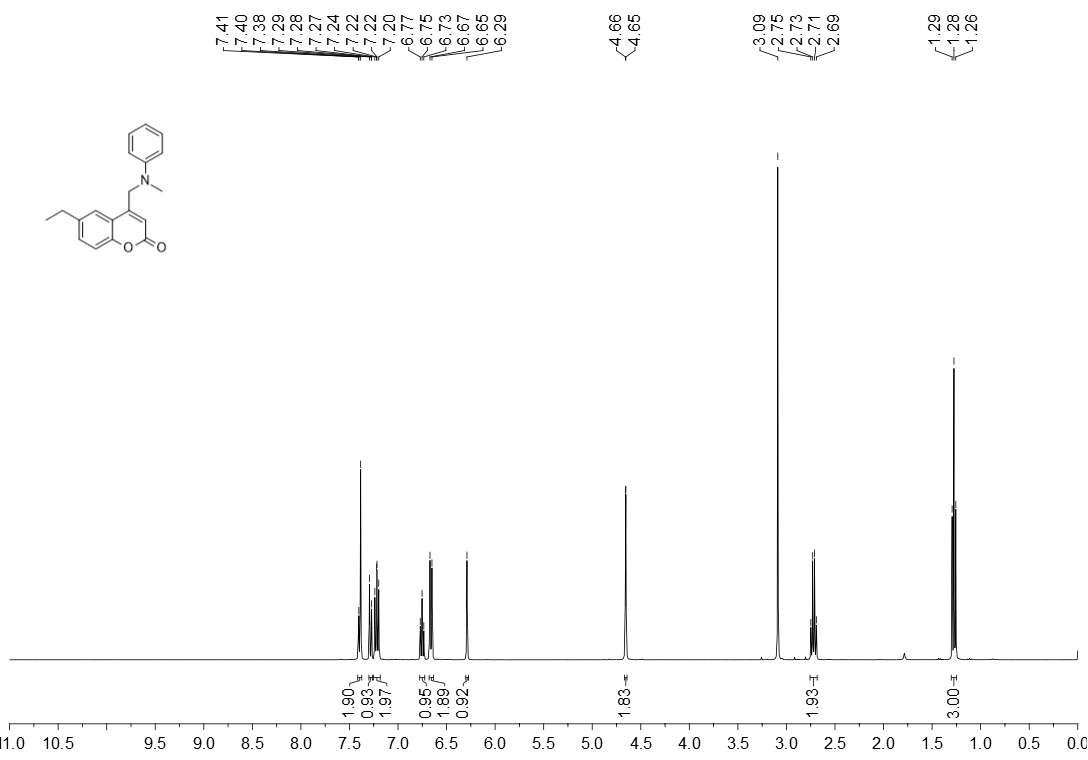


**Figure S58.** ^1^H NMR spectrum (300 MHz, CDCl_3_) of compound **11g**


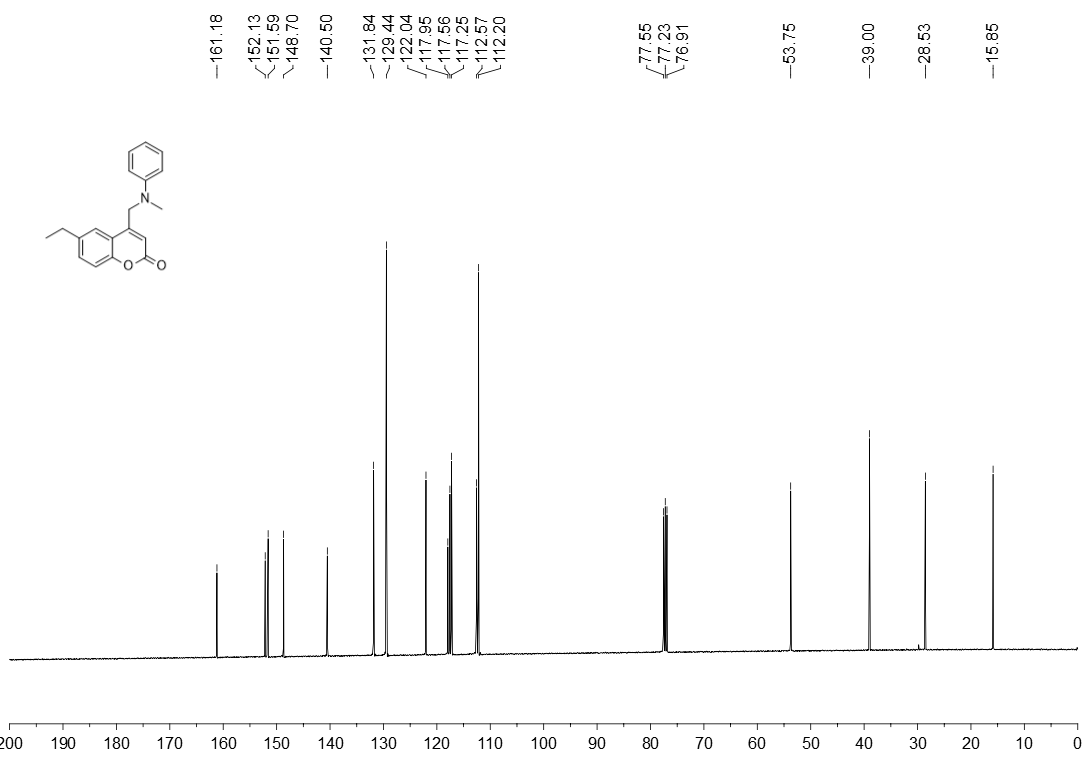


# **Figure S59**. ^13^C NMR spectrum (75 MHz, CDCl_3_) of compound **11g.**

#
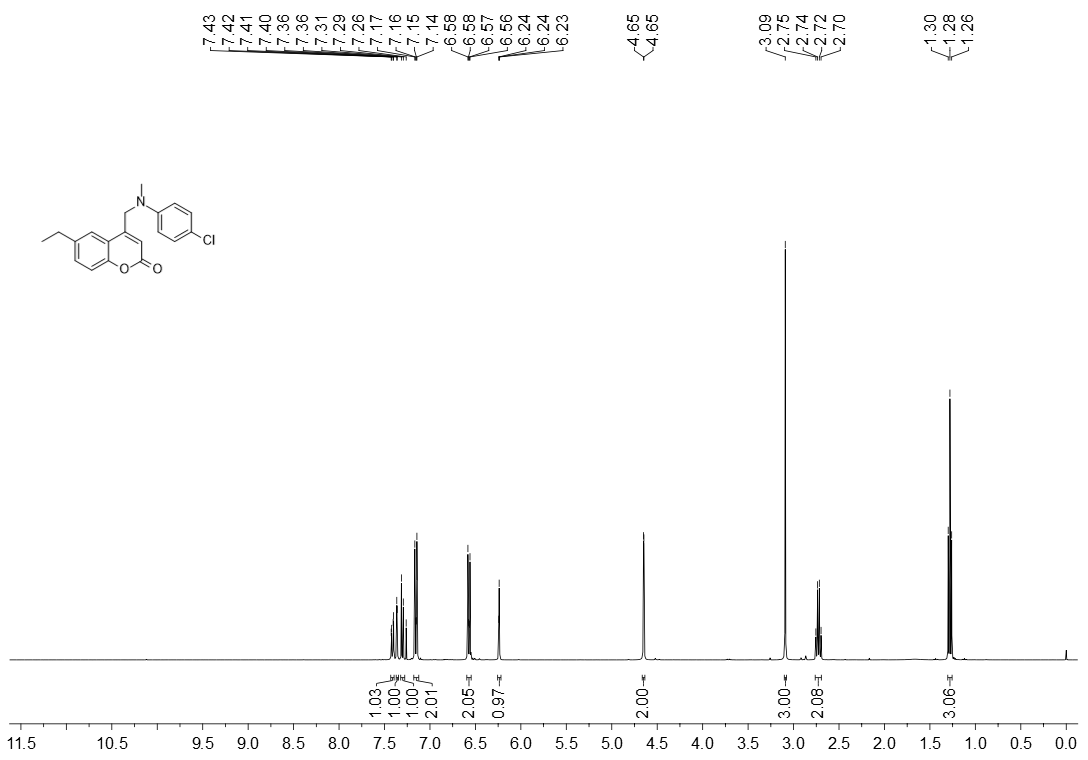


**Figure S60.** ^1^H NMR spectrum (400 MHz, CDCl_3_) of compound **11h**


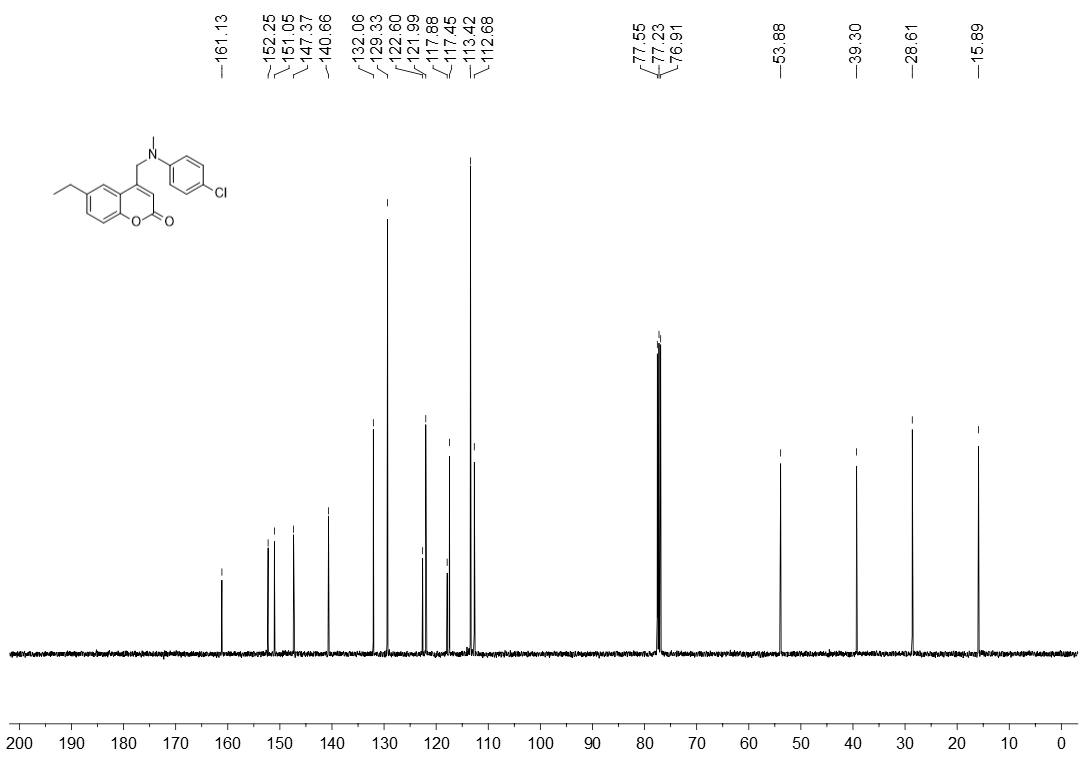


# **Figure S61**. ^13^C NMR spectrum (100 MHz, CDCl_3_) of compound **11h.**


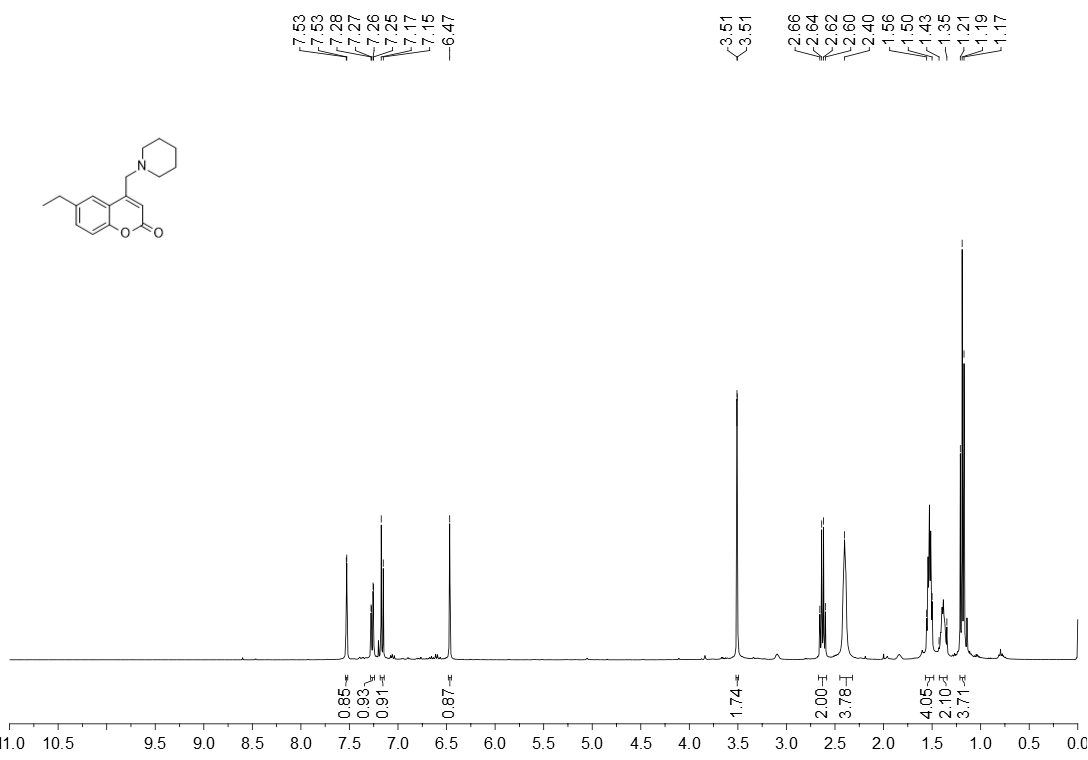


**Figure S62.** ^1^H NMR spectrum (300 MHz, CDCl_3_) of compound **11i**


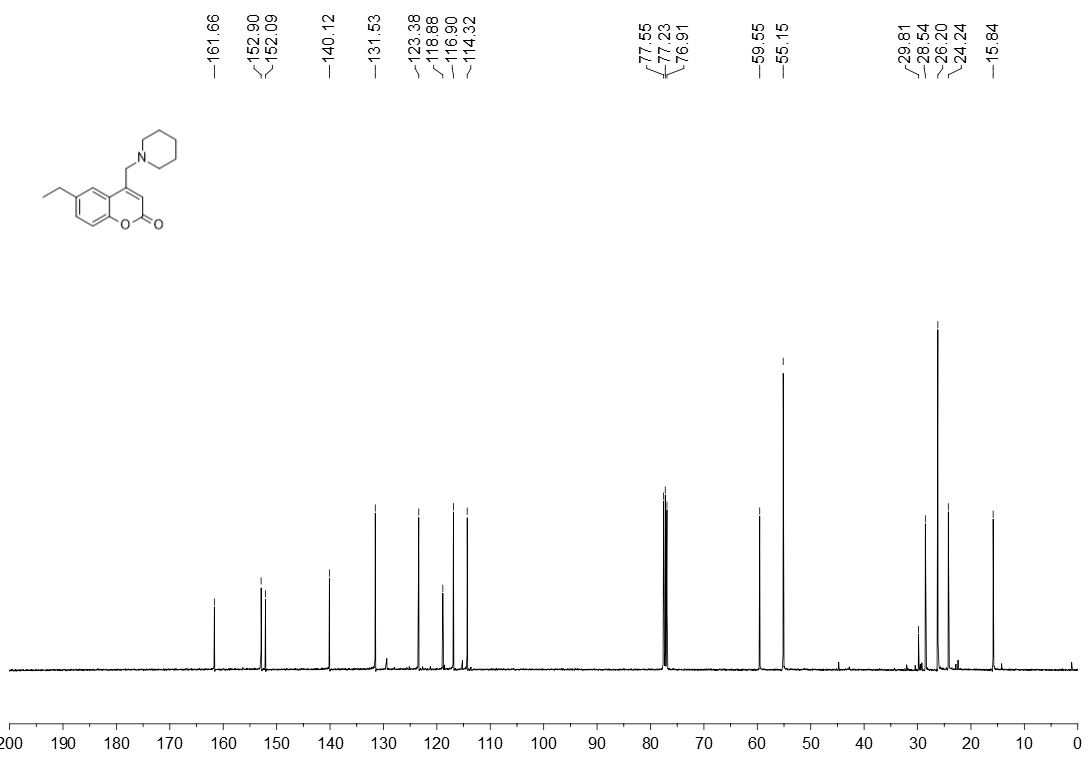


# **Figure S63**. ^13^C NMR spectrum (75 MHz, CDCl_3_) of compound **11i.**


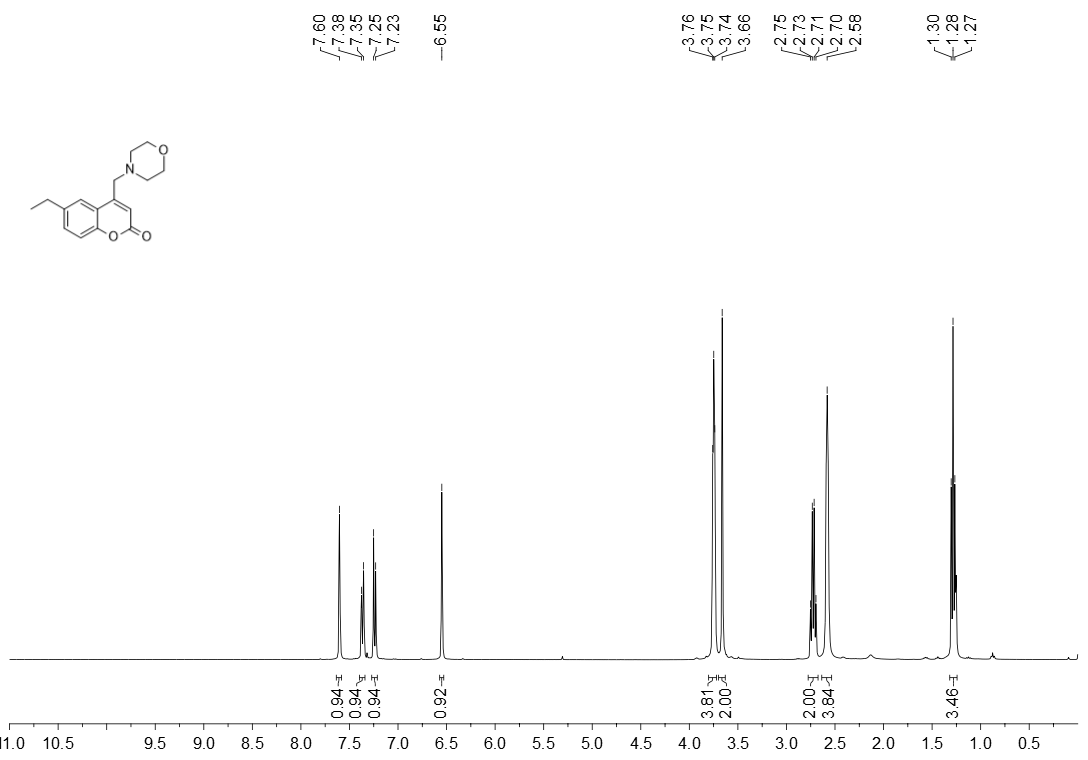


**Figure S64.** ^1^H NMR spectrum (300 MHz, CDCl_3_) of compound **11k**


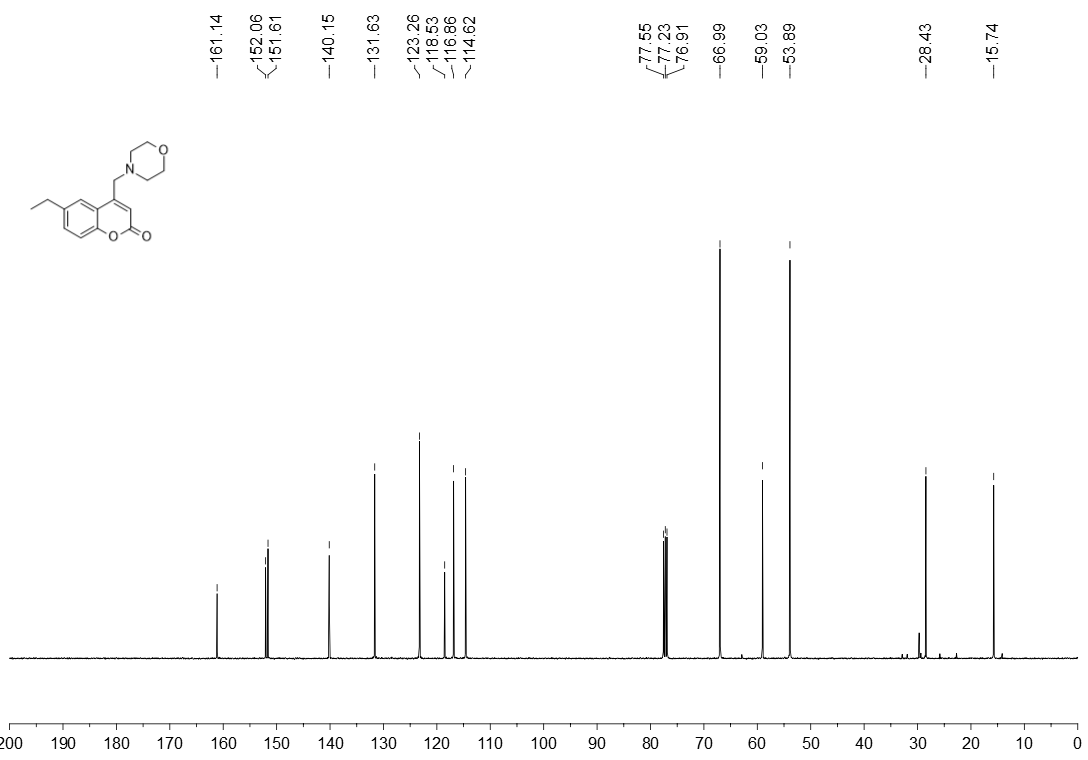


# **Figure S65**. ^13^C NMR spectrum (75 MHz, CDCl_3_) of compound **11k.**


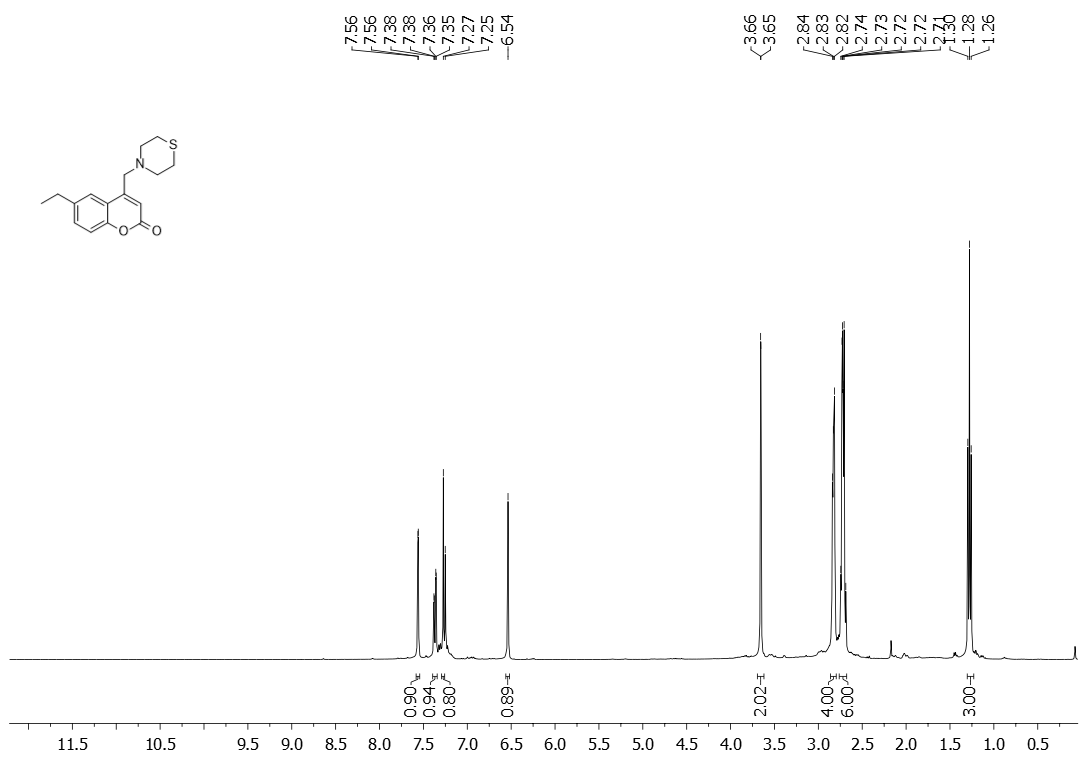


**Figure S66.** ^1^H NMR spectrum (300 MHz, CDCl_3_) of compound **11l**


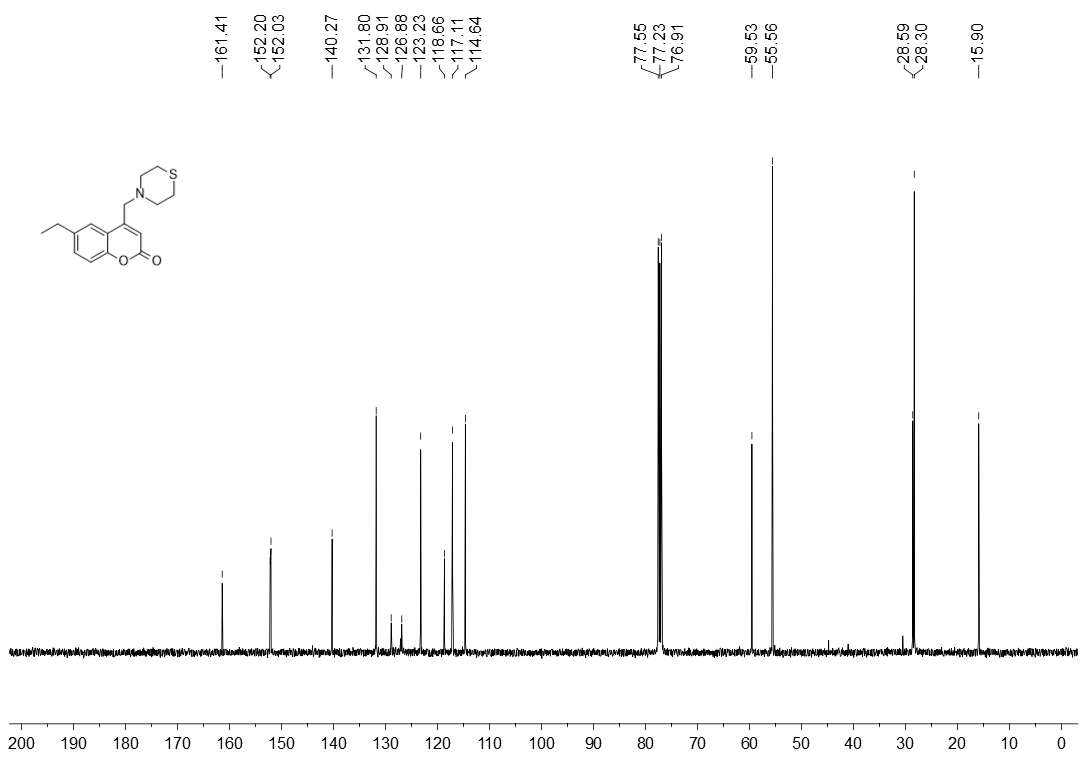


# **Figure S67**. ^13^C NMR spectrum (75 MHz, CDCl_3_) of compound **11l.**


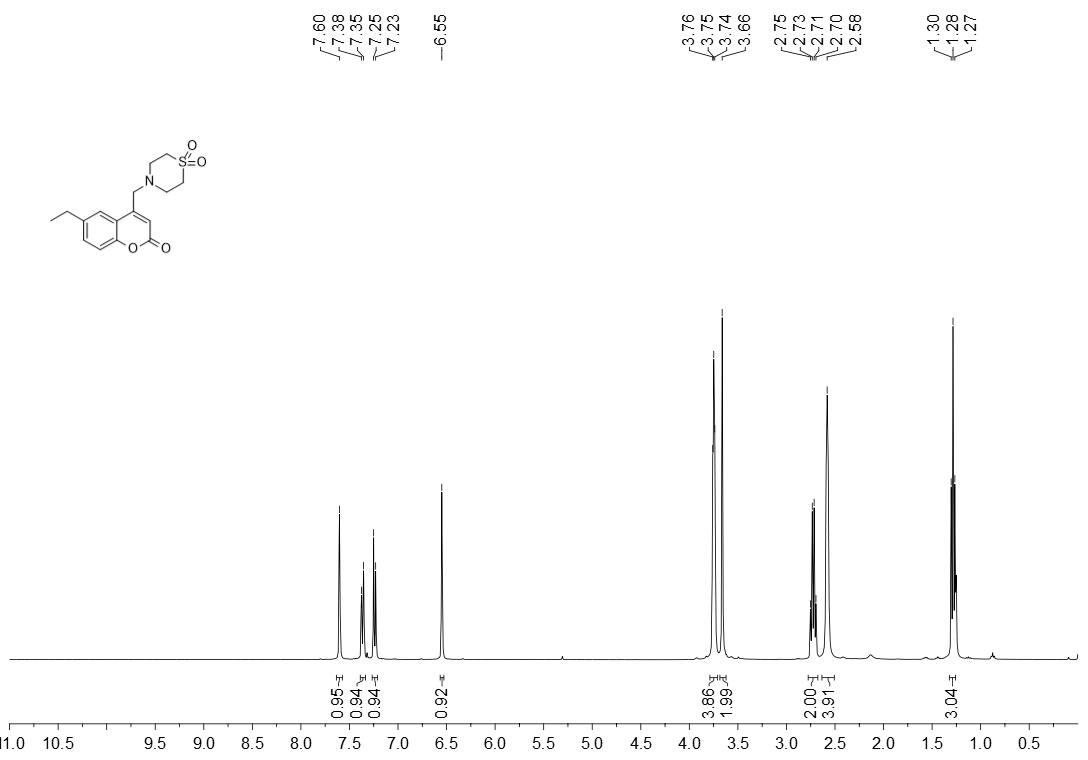


**Figure S68.** ^1^H NMR spectrum (300 MHz, CDCl_3_) of compound **11m**


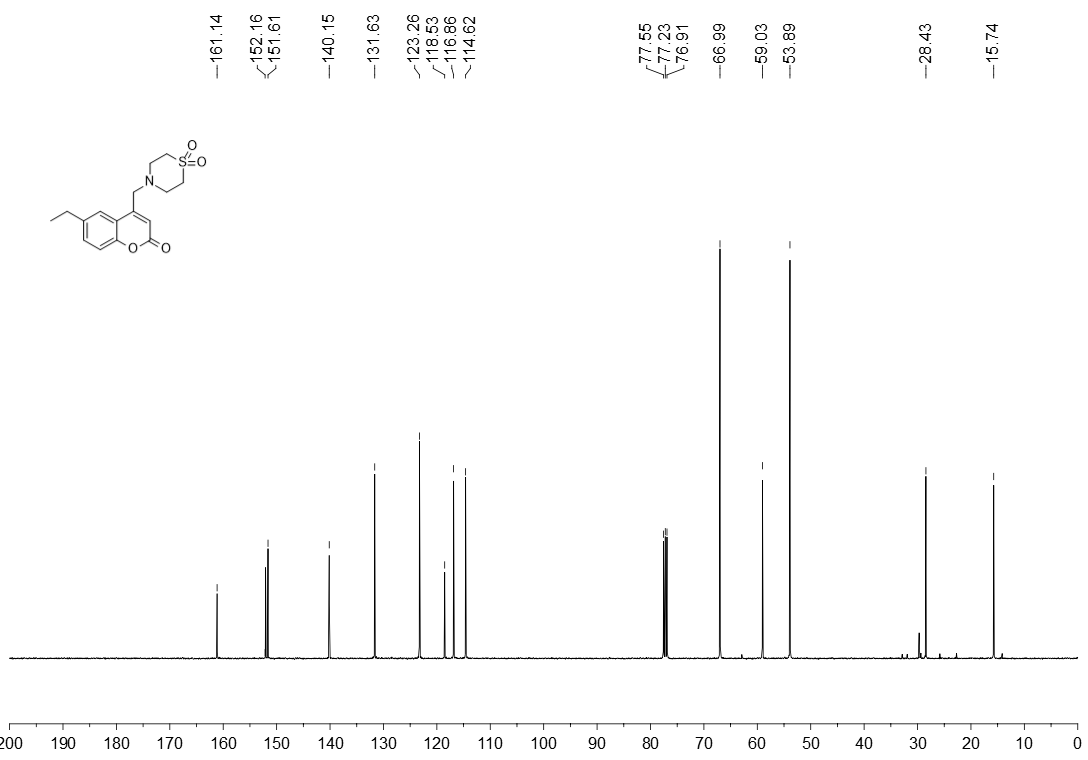


# **Figure S69**. ^13^C NMR spectrum (75 MHz, CDCl_3_) of compound **11m.**


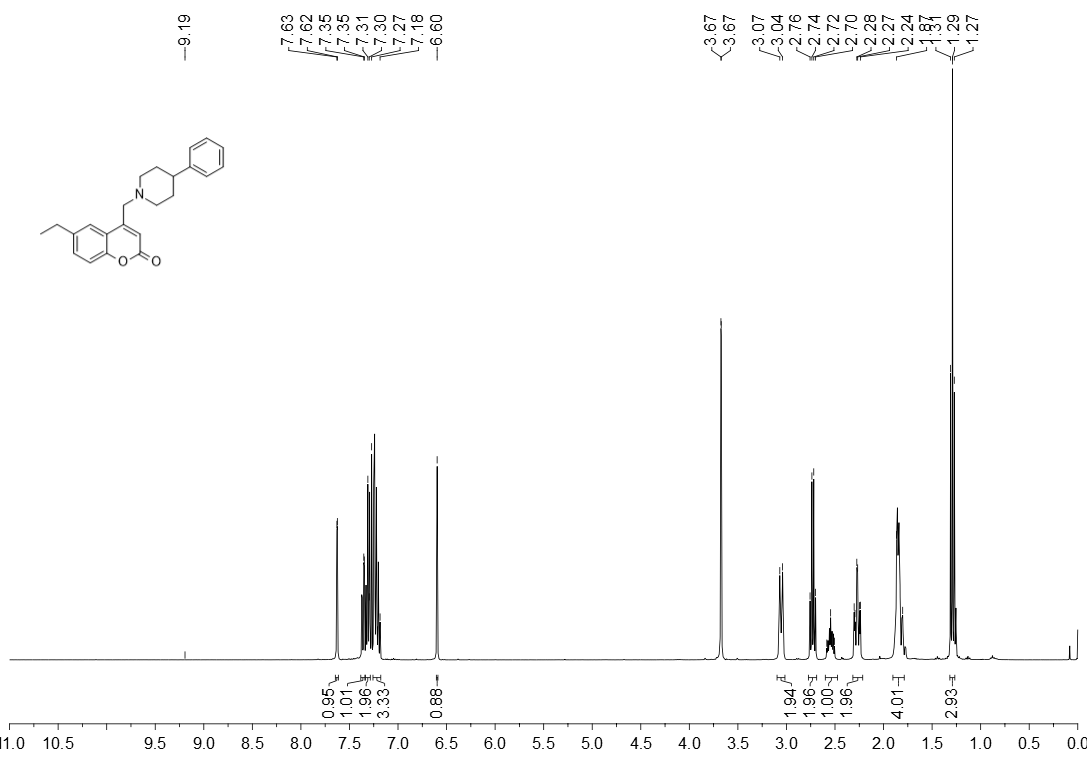


**Figure S70.** ^1^H NMR spectrum (300 MHz, CDCl_3_) of compound **11n**


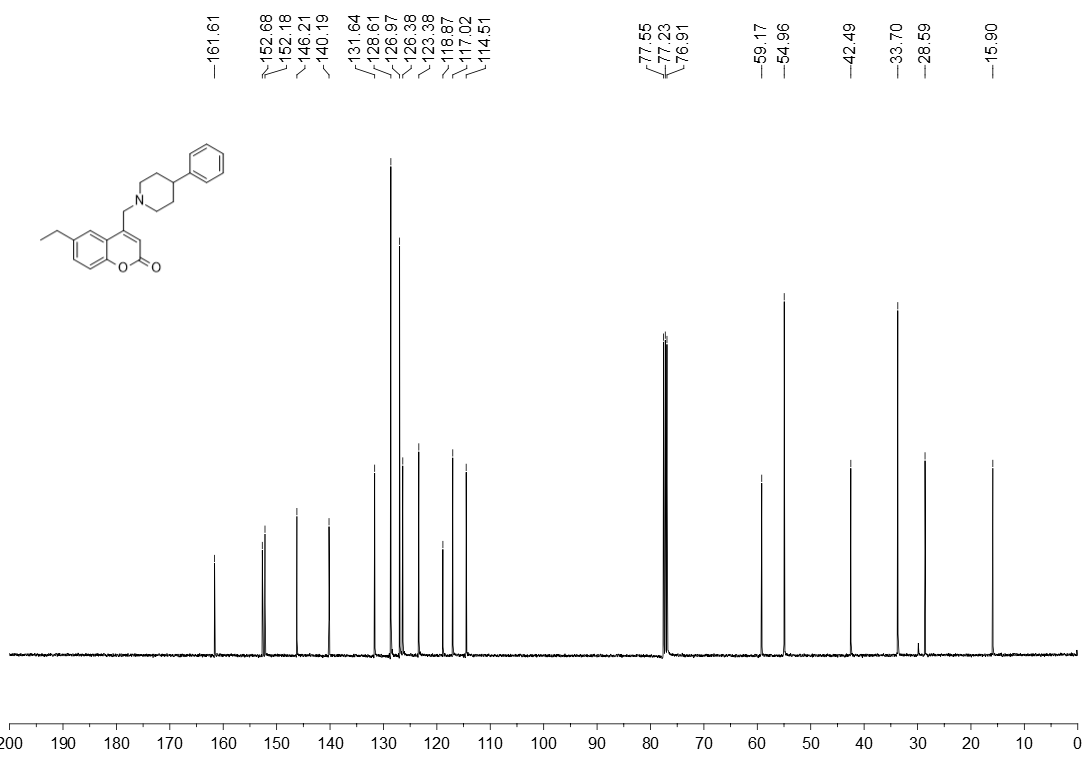


# **Figure S71**. ^13^C NMR spectrum (75 MHz, CDCl_3_) of compound **11n.**
